# Hologenome analysis reveals dual symbiosis in the deep-sea hydrothermal vent snail *Gigantopelta aegis*

**DOI:** 10.1038/s41467-021-21450-7

**Published:** 2021-02-19

**Authors:** Yi Lan, Jin Sun, Chong Chen, Yanan Sun, Yadong Zhou, Yi Yang, Weipeng Zhang, Runsheng Li, Kun Zhou, Wai Chuen Wong, Yick Hang Kwan, Aifang Cheng, Salim Bougouffa, Cindy Lee Van Dover, Jian-Wen Qiu, Pei-Yuan Qian

**Affiliations:** 1 Department of Ocean Science, Division of Life Science and Hong Kong Branch of the Southern Marine Science and Engineering Guangdong Laboratory (Guangzhou), The Hong Kong University of Science and Technology, Hong Kong, China; 2 Southern Marine Science and Engineering Guangdong Laboratory (Guangzhou), Guangzhou, China; 3 X-STAR, Japan Agency for Marine-Earth Science and Technology (JAMSTEC), Yokosuka, Kanagawa Prefecture Japan; 4 Key Laboratory of Marine Ecosystem Dynamics, Second Institute of Oceanography, Ministry of Natural Resources, Hangzhou, China; 5 College of Marine Life Science, Ocean University of China, Qingdao, China; 6 Department of Infectious Diseases and Public Health, Jockey Club College of Veterinary Medicine and Life Sciences, City University of Hong Kong, Hong Kong, China; 7 Computational Bioscience Research Centre, King Abdullah University of Science and Technology, Thuwal, Saudi Arabia; 8 King Abdullah University of Science and Technology (KAUST), Core Labs, Thuwal, Saudi Arabia; 9 Division of Marine Science and Conservation, Nicholas School of the Environment, Duke University, Beaufort, NC United States; 10 Department of Biology and Hong Kong Branch of the Southern Marine Science and Engineering Guangdong Laboratory (Guangzhou), Hong Kong Baptist University, Hong Kong, China

**Keywords:** Genomics, Bacterial genomics, Symbiosis

## Abstract

Animals endemic to deep-sea hydrothermal vents often form obligatory symbioses with bacteria, maintained by intricate host–symbiont interactions. Most genomic studies on holobionts have not investigated both sides to similar depths. Here, we report dual symbiosis in the peltospirid snail *Gigantopelta aegis* with two gammaproteobacterial endosymbionts: a sulfur oxidiser and a methane oxidiser. We assemble high-quality genomes for all three parties, including a chromosome-level host genome. Hologenomic analyses reveal mutualism with nutritional complementarity and metabolic co-dependency, highly versatile in transporting and using chemical energy. *Gigantopelta aegis* likely remodels its immune system to facilitate dual symbiosis. Comparisons with *Chrysomallon squamiferum*, a confamilial snail with a single sulfur-oxidising gammaproteobacterial endosymbiont, show that their sulfur-oxidising endosymbionts are phylogenetically distant. This is consistent with previous findings that they evolved endosymbiosis convergently. Notably, the two sulfur-oxidisers share the same capabilities in biosynthesising nutrients lacking in the host genomes, potentially a key criterion in symbiont selection.

## Abstract (web summary)

Symbiotic partners are rarely studied in equal depth. By assembling new genomes, Lan *et al*. report a novel dual symbiosis in the snail *Gigantopelta aegis* with two evolutionarily distant gammaproteobacterial endosymbionts: one which oxidises sulfur, the other, methane in a metabolically mutualistic relationship.

## Introduction

Animals live with microorganisms in and around them, forming ecological units referred to as holobionts. Some metazoans, such as reef-forming corals, have established intricate obligatory symbiotic relationships with single-celled organisms that enable the holobiont to utilise available energy sources more effectively^1^. In deep-sea chemosynthetic environments such as hydrothermal vents, many endemic animals harbour endosymbiotic bacteria within their cells to access energy and nutrients released through microbial oxidation of reducing substances such as hydrogen sulfide, hydrogen, thiosulfate, and methane^2^. Most of these, such as the peltospirid Scaly-foot Snail *Chrysomallon squamiferum*, the giant vesicomyid clam *Turneroconcha magnifica*, as well as the siboglinid tubeworms *Riftia pachyptila* and *Paraescarpia echinospica*, have evolved obligate symbiotic relationships with one dominant phylotype of microbial endosymbiont^3–6^. By contrast, several species of annelids and molluscs, including the thyasirid clam ‘*Maorithyas*’ *hadalis*, the provannid snail genus *Alviniconcha*, some *Bathymodiolus* mussels, and the shallow-water oligochaete worm *Olavius*, are capable of hosting multiple co-occurring endosymbionts^7–11^. A number of studies have used genomic tools to explore the molecular mechanisms that enable and support chemosynthetic symbioses, but most focused on either the symbiont or the host^4,5,12^, while only a few studies discussed both sides (e.g. the tubeworms *R. pachyptila*^3^ and *P. echinospica*^6^ as well as the flatworm *Paracatenula*^13^). Adopting a total ‘hologenome’ approach in sequencing the host and the symbiont genomes combined with a transcriptomic approach that examines organ-specific gene expression enables us to disentangle the complex metabolic relationships of the partners, allowing them to flourish in extreme environments.

Among deep-sea vent molluscs housing endosymbionts, the two peltospirid genera (Neomphalida: Peltospiridae) *Gigantopelta*^14^ and *Chrysomallon*^15,16^ host symbionts inside a specialised organ within the gut (a modified oesophageal gland) rather than in the gill epithelial cells like all others, including *Bathymodiolus* mussels^17^, vesicomyid clams^5^, and provannids snails^8^. The endosymbionts housed in internal organs without direct contact with the vent fluid are likely to be reliant on the host for transporting substances such as oxygen, sulfide, and methane; although diffusion may also play a role in their delivery to the symbionts. The anatomical organisation of the endosymbionts in these two peltospirid snails are more similar to tubeworms than other known molluscan symbioses in vents^14,18^ and indeed, the endosymbionts housed in the internal trophosome of the giant tubeworm *R. pachyptila* rely on the host’s haemoglobins to transport oxygen and sulfide^19^.

Peltospiridae is a family of gastropods endemic to deep-sea hydrothermal vents^16^. It currently has 11 recognised genera, but only *Gigantopelta* and *Chrysomallon* are known to have enlarged oesophageal glands hosting endosymbionts for nutrition, while the rest are grazers, filter-feeders, or deposit feeders^20^. Snails in both genera appear to obtain their endosymbionts from the environment through horizontal transmission in each generation^4,21^. Previous multi-gene phylogenetic analyses have revealed that *Gigantopelta* and *Chrysomallon* are not the most closely related peltospirid genera, and the two are separated by a number of other much smaller, non-endosymbiotic genera such as *Peltospira*, *Nodopelta, Rhynchopelta*, and *Dracogyra*^14,20^. Despite their superficial similarity, *Gigantopelta* and *Chrysomallon* are also quite different in their internal anatomy^14^. A crucial difference between the two is that while *Chrysomallon* relies on endosymbionts for nutrition throughout its post-settlement life^4,16^, *Gigantopelta* initially adopts grazing for nutrients and only later (at around 5–7 mm shell length) rapidly shifts to hosting endosymbionts^21^. This process, dubbed ‘cryptometamorphosis’, occurs with a dramatic reorganisation of its digestive system where the oesophageal gland is greatly enlarged while the other parts of the digestive system effectively stop growing^21^. Thus, these two genera have evolved an endosymbiotic lifestyle independently and convergently, rather than from a common ancestor with symbionts^14^.

The fact that representatives of the two genera, *Gigantopelta aegis* and the Scaly-foot Snail *C. squamiferum*, co-occur in great abundance in the Longqi vent field on the ultra-slow spreading Southwest Indian Ridge makes them ideal candidates to compare molecular adaptations in the two holobiont systems. From previous studies, we know that *C. squamiferum* hosts only one phylotype of sulfur-oxidising Gammaproteobacteria whose genome has been sequenced^4^. This genome shows that the symbionts of *C. squamiferum* are capable of sulfur oxidation, carbon fixation, and nutrient biosynthesis^4^. The whole genome of *C. squamiferum* was recently sequenced and assembled to chromosome-scale, but the study focused on the origin of its unique biomineralisation, leaving adaptations to symbiosis mostly unexamined^22^. Previously, 16S rRNA sequencing of the closely related *Gigantopelta chessoia* only revealed a single sulfur-oxidising Gammaproteobacteria symbiont^23^, although transmission electron microscopy (TEM) micrographs of the oesophageal gland hinted a second, rarer, likely methane-oxidising symbiont^14^.

Here, we reveal that *G. aegis* exhibits dual endosymbiosis housing two Gammaproteobacteria endosymbionts, one sulfur-oxidising and one methane-oxidising, in its oesophageal gland. We use high-quality genome assemblies of both the *G. aegis* snail host and its two physiologically distinct endosymbionts to elucidate interactions and co-operations among this ‘triptych’ of parties in this holobiont. Furthermore, we compare the hologenome of *G. aegis* with that of *C. squamiferum* to understand how *G. aegis* benefits from maintaining symbiosis with more than one endosymbiont.

## Results and discussion

### Features of a holobiont with three parties

**Fig. 1:**
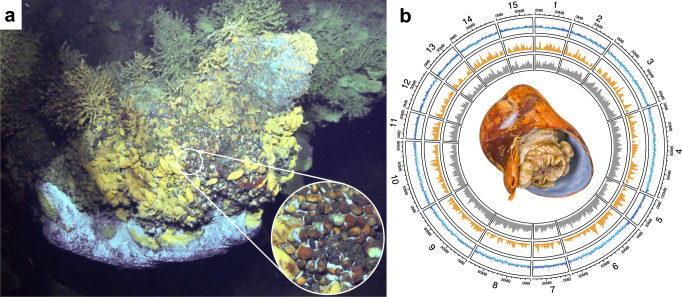
The genome landscape of the snail *Gigantopelta aegis* living in Tiamat chimney. **a** Two chemosymbiotic peltospirid snails, *Gigantopelta aegis* and the Scaly-foot Snail *Chrysomallon squamiferum*, occurring in great abundance side-by-side at Tiamat chimney in the Longqi vent field on the ultra-slow spreading Southwest Indian Ridge (*G. aegis* in dark orange; *C. squamiferum* in black). **b** A circos plot shows key features of 15 pseudo-chromosomal linkage groups of the *G. aegis* genome showing an adult snail at the centre, the inner circle (grey) indicating the gene density of each pseudo-chromosome, the second circle (orange) indicating the gene density of highly expressed genes in the oesophageal gland (*n* = 4), and the outer circle (blue) showing the GC content of the deviation from the average 37.22%. The outer bars show the chromosome length. A sliding window of 100 kb in a step of 50 kb was applied in the calculation of the GC content.

Members of the two peltospirid genera, *Gigantopelta aegis* and the Scaly-foot Snail *Chrysomallon squamiferum*, occur side-by-side at Tiamat chimney in the Longqi vent field (Fig. 1a). We obtained high-quality genome assemblies of all three parties in the *G. aegis* holobiont using an adult snail for in-depth analyses of its symbiosis. The host genome is 1.15 Gb in size, comprising 15 pseudo-chromosomes with 94.6% genome completeness (Fig. 1b, Supplementary Table 1, and Supplementary Note 1). This is one of the few chromosome-level genome assemblies currently available in Gastropoda, or even across Mollusca. The genome size of *G. aegis* is more than double that of *C. squamiferum* (~444.4 Mb)^22^ mainly due to the larger proportion of repetitive regions (Supplementary Fig. 1 and Supplementary Table 2). A total of 21,438 genes (Supplementary Data 1) were predicted from the *G. aegis* genome, of which 1782 genes were highly expressed in the symbiont-hosting oesophageal gland (Supplementary Data 2). No particular chromosomal distribution bias was observed among these genes (Fig. 1b).

**Fig. 2:**
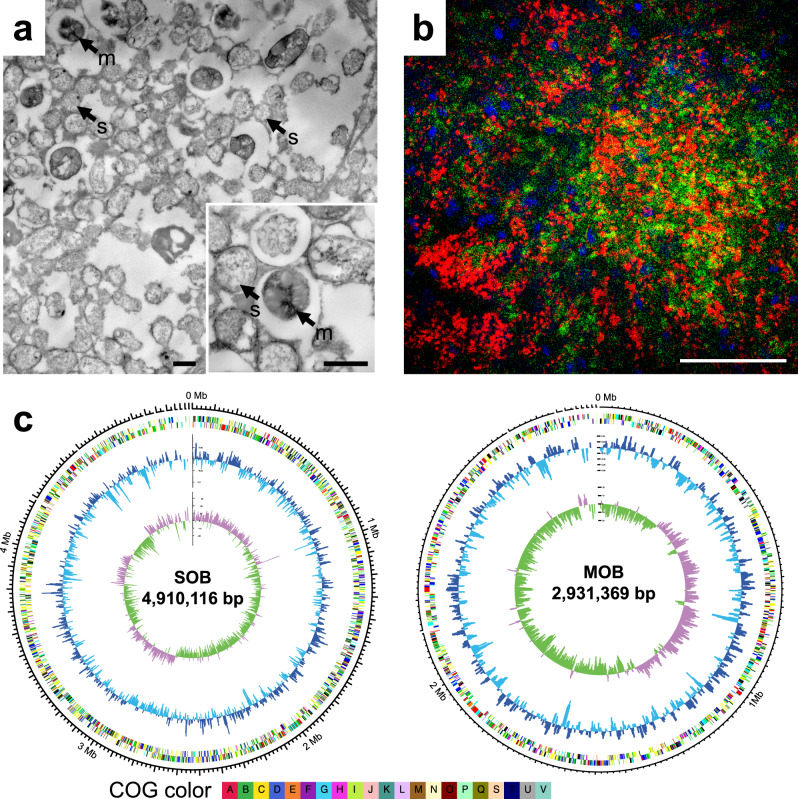
Dual symbionts of *Gigantopelta aegis* snail. **a** Transmission electron microscopy (TEM) images of intracellular endosymbionts showing two distinct morphological types: one endosymbiont with intracellular stacked membranes (black arrows; m: methane-oxidising symbiont) and another without (black arrows; s: sulfur-oxidising symbiont; scale bar: 1 µm). **b** Fluorescence in situ hybridisation (FISH) image yielding signals of sulfur-oxidising endosymbionts (SOB; Cy3: green) and methane-oxidising endosymbionts (MOB; Cy5: red) on transverse sections of the oesophageal gland from *Gigantopelta aegis* (scale bar: 50 µm). Host nuclear DNA with DAPI staining is blue. More detailed TEM and FISH images are shown in Supplementary Fig. 2. **c** Key genome features of both the SOB and the MOB of *G. aegis* with genome sizes in the centre, the inner circle showing the GC skew (minus value: green value, plus: purple), the second circle showing the GC content of the deviation from the average (SOB: 39.93%; MOB: 45.26%; lower than average GC content: light blue, higher than average: dark blue), and the outer circle showing the COG annotation categories with different colours. The details of COG annotation are shown in Supplementary Data 3 and 4. A sliding window of 5 kb in a step of 2.5 kb was applied in the calculation of both the GC skew and the GC content. TEM experiments were applied on three individuals with more than three thin sections each. FISH experiments were repeated independently for twice and at least ten sections of the samples were used for each time. These experiments were repeated with similar results. The description of COG annotation categories are provided in Supplementary Fig. 21.

TEM micrographs of the oesophageal gland from *G. aegis* confirmed the existence of intracellular endosymbionts densely packed inside bacteriocytes (Fig. 2a and Supplementary Fig. 2). Two types of endosymbionts with distinct morphological features were found within a single bacteriocyte (Fig. 2a). Sequences of the 16S ribosomal RNA from the oesophageal gland of three *G. aegis* individuals were consistent in revealing the two types of symbionts belonging to Gammaproteobacteria, with the more abundant one being a sulfur-oxidising bacteria (SOB hereafter) and the less common one being a methane-oxidising bacteria (MOB hereafter) in the genus *Methylomarinum* of the family Methylococcaceae (Supplementary Fig. 3). The SOB clustered with uncultured bacteria phylogenetically (Supplementary Fig. 4), and thus its taxonomic affinity remains unclear. Of the two symbionts identified from TEM, one exhibited stacked intracellular membranes (Fig. 2a), a key characteristic of Type I methanotrophs^17^, while the other lacked these membranes. Fluorescence in situ hybridisation (FISH) carried out on transverse sections of the oesophageal gland from *G. aegis* yielded positive signals of the specific probes targeting the SOB and the MOB, respectively (Fig. 2b and Supplementary Fig. 2). These results confirmed that *G. aegis* is a holobiont with three active parties: the host and two physiologically distinct gammaproteobacterial endosymbionts. Genome binning results showed that the SOB, the MOB, and the host could be separated with high fidelity by their GC content and sequencing coverage (Supplementary Fig. 5). The TEM results and the sequencing coverage indicate that both endosymbionts are abundant in the oesophageal gland, with the SOB being approximate seven times more common than the MOB in this individual (Supplementary Table 3). However, the relative abundance of the two symbionts possibly vary depending on the concentrations and availability of reducing chemicals in the ambient environment across spatial or temporal scales^24,25^, as observed in *Bathymodiolus* mussels which possess two phylotypes of symbionts^26,27^.

The assembled genomes of the SOB (98.55% genome completeness; 11 scaffolds of 4.91 Mb; 5518 genes) and the MOB (99.25% genome completeness; 10 scaffolds of 2.93 Mb; 3102 genes) (Supplementary Table 3 and Supplementary Data 3 and 4) exhibited different genomic features in terms of GC content (SOB: 39.93%; MOB: 45.26%), GC skew, and annotated Clusters of Orthologous Groups (COG) (Fig. 2c; for details of assembly and functional annotation see Supplementary Note 2). CheckM analysis confirmed the high quality of the two symbiont genomes, as the potential contamination was low (3.25% in SOB and 1.67% in MOB; Supplementary Table 3). In the metaproteomic analyses of the three parties, 704 proteins were identified in the host, 462 proteins were identified in the SOB, and 119 proteins were identified in the MOB (Supplementary Data 5), providing additional protein-based evidence to trace the metabolism of the *G. aegis* holobiont.

### Evolution of symbiosis in deep-sea peltospirid snails

**Fig. 3:**
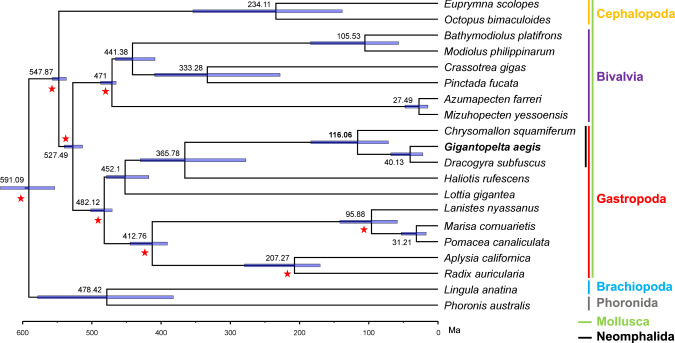
Divergent time of 20 lophotrochozoan taxa. Time-calibrated phylogeny showing the estimated divergence time of 20 lophotrochozoan taxa, with a focus on 18 molluscs. *Phoronis australis* (Phoronida) and *Lingula anatina* (Brachiopoda) served as outgroups for the molluscs. A total of 529 single-copy orthologues were used. The purple horizontal lines indicate the 95% confidence intervals of the divergence times. Full information on genome resources, fossil records, and geographic events used in the time calibrated nodes (red stars) are provided in Supplementary Note 3. The colour labelling scheme of taxa: Cephalopoda: orange; Bivalvia: purple; Gastropoda: red; Brachiopoda: blue; Phoronida: grey; Mollusca: green; and Neomphalida: black. Ma: millions of years ago.

Dated and fossil-calibrated phylogenetic reconstruction based on 529 single-copy genes suggest that the two peltospirid snails, *G. aegis* and *C. squamiferum*, diverged 116.06 million years ago (Ma; 95% confidence intervals: 71.4–183.6 Ma) (Fig. 3 and Supplementary Note 3), which was consistent with a previous study that the ancestor of peltospirids extends to the Early Cretaceous, and the order Neomphalida likely had a mid-Jurassic origin^14^. These early fossils of peltospirids from Early Cretaceous seep deposits had small body size and unlikely to have been symbiotic^28^. As such, the fossil history also supports the findings that *Gigantopelta* and *Chrysomallon* probably evolved symbiosis separately after the current radiation of Neomphalida and Peltospiridae began.

**Fig. 4:**
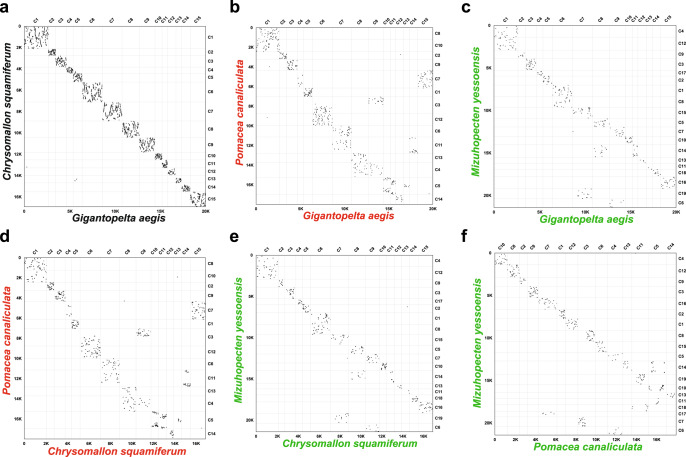
Chromosome-scale synteny among four Mollusca genomes. Chromosome-scale synteny is shown in dot plots with comparisons among the genomes of *Gigantopelta aegis* (15 chromosomes), *Chrysomallon squamiferum* (15 chromosomes), *Pomacea canaliculata* (14 chromosomes), and *Mizuhopecten yessoensis* (19 chromosomes). These plots include the comparison **a** between *G. aegis* and *C. squamiferum* (comparison within Neomphalida: black), **b** between *G. aegis* and *P. canaliculata* (comparison within Gastropoda: red), **c** between *G. aegis* and *M. yessoensis* (comparison within Mollusca: green); **d** between *C. squamiferum* and *P. canaliculata* (comparison within Gastropoda: red), **e** between *C. squamiferum* and *M. yessoensis* (comparison within Mollusca: green), and **f** between *P. canaliculata* and *M. yessoensis* (comparison within Mollusca: green). Each dot represents a synteny block. The numbers on the left and the bottom are the accumulation of the genes in the chromosomes. The colour scheme follows the taxon indicator colours in Fig. 3. C: chromosome. Source data of **a**–**f** are provided as a Source Data file.

The genomes of both *G. aegis* and *C. squamiferum* snails had 11 *hox* gene clusters in the same order (Supplementary Fig. 6), 13 protein-coding genes of the mitogenome in the same order (Supplementary Fig. 7) and highly consistent macro-synteny patterns (Fig. 4). These results indicate that majority of the gene order and synteny were conserved within the family Peltospiridae, and such clear correspondences exemplify the high quality of both genome assemblies. In addition, the genomic rearrangements revealed by micro-synteny blocks were exclusively intra-chromosomal, rather than inter-chromosomal (Fig. 4). Given the small number of chromosome-level genome assemblies within molluscs, we are unable to test if such a high macro-synteny conservation is the norm within the group for a similar divergence time. Nevertheless, the macro-synteny patterns in these two species are also notably conserved compared with the caenogastropod *Pomacea canaliculata* and the bivalve *Mizuhopecten yessoensis*, distantly related to peltospirid snails within Mollusca^29,30^. In most molluscan lineages, intra-chromosomal rearrangements likely underpin the evolution of genetic regulatory networks and related functions, but the underlying mechanism warrants further studies. Among other molluscs with chromosome-level assembly, the oyster *Crassostrea gigas* uniquely has a much reduced genome size and highly derived karyotype^30^, but it is likely an exception rather than the norm among molluscs.

A third, much smaller, peltospirid snail, *Dracogyra subfuscus*, co-occurs with *G. aegis* and *C. squamiferum* in the Longqi vent field, but anatomical investigation showed that it neither has an enlarged oesophageal gland nor relies on symbionts for nutrition^20^. Previous phylogeny showed that it is one of many non-symbiotic peltospirid genera separating *Chrysomallon* and *Gigantopelta*, and the sister genus of *Gigantopelta*^20^. We assembled a draft genome of *D. subfuscus* (Supplementary Note 7) and conducted genome binning (Supplementary Fig. 8) to substantiate these claims^20^. Our results confirmed that *D. subfuscus* is non-symbiotic and much more closely related to *G. aegis* with an average divergent time of 40.13 Ma (Fig. 3) than *C. squamiferum*. These results were in line with previous findings suggesting that *G. aegis* and *C. squamiferum* evolved symbiosis through independent and convergent evolution^14,20^. Although some *Bathymodiolus* mussels also house dual symbionts of sulfur-oxidising symbionts and methane-oxidising symbionts^31^, *Bathymodiolus* mussels likely had a common symbiotic ancestor before their radiation in the deep sea^32,33^.

**Fig. 5:**
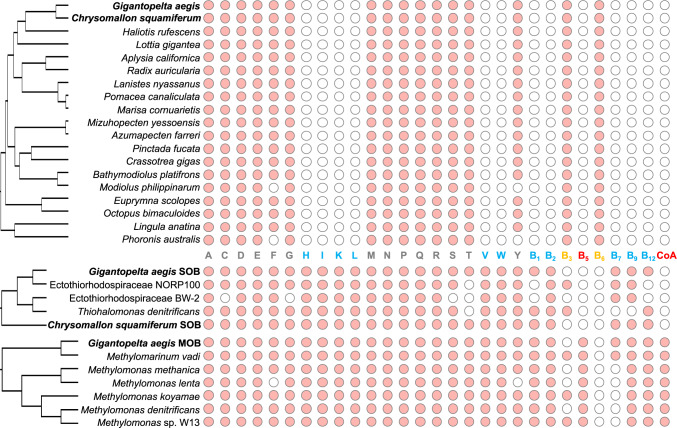
Biosynthesis capabilities of nutrients in *Gigantopelta aegis* holobiont and its relatives. Presence (pink circle) and absence (white circle) of biosynthesis capabilities of 20 amino acids, 8 vitamins and coenzyme A in *Gigantopelta aegis* and 18 other molluscan taxa, as well as *G. aegis* symbionts and their free-living relatives. A: alanine, C: cysteine, D: aspartate, E: glutamate, F: phenylalanine, G: glycine, H: histidine, I: isoleucine, K: lysine, L: leucine, M: methionine, N: asparagine, P: proline, Q: glutamine, R: arginine, S: serine, T: threonine, V: valine, W: tryptophan, Y: tyrosine, B_1_: vitamin B_1_, B_2_: vitamin B_2_, B_3_: vitamin B_3_, B_5_: vitamin B_5_, B_6_: vitamin B_6_, B_7_: vitamin B_7_, B_9_: vitamin B_9_, B_12_: vitamin B_12_, CoA: coenzyme A. The colour labelling scheme of nutrients: blue (H, I, K, L, V, W, B_1_, B_2,_ B_7_, B_9_, and B_12_) represents nutrients that can be synthesised by both the sulfur-oxidising endosymbionts (SOB) and the methane-oxidising endosymbionts (MOB) but host cannot; orange (B_3_ and B_6_) represents nutrients that can only be synthesised by the host; red (B_5_ and CoA) represents that the nutrients that can only be synthesised by the MOB; grey represents the nutrients can be synthesised by host, SOB, and MOB.

Despite the phylogenetic divergence between the sulfur-oxidising endosymbionts in the two endosymbiotic peltospirid snails *G. aegis* and *C. squamiferum* (Supplementary Fig. 9), the two SOBs were similar in their gene contents encoding for nutritional metabolism, and the same goes for the two host snails (Fig. 5). Phylogenetic divergence of hosts and their SOBs and the convergence of host and SOB gene contents relating to nutritional metabolism were consistent with the working hypothesis that the two peltospirid snails established their symbiotic relationships through convergent evolutionary pathways. Selection of the SOB symbionts appears to have favoured the retention of biosynthesis of specific nutrients that the hosts are unable to synthesise.

### Genetic control of symbiont acquisition

**Fig. 6:**
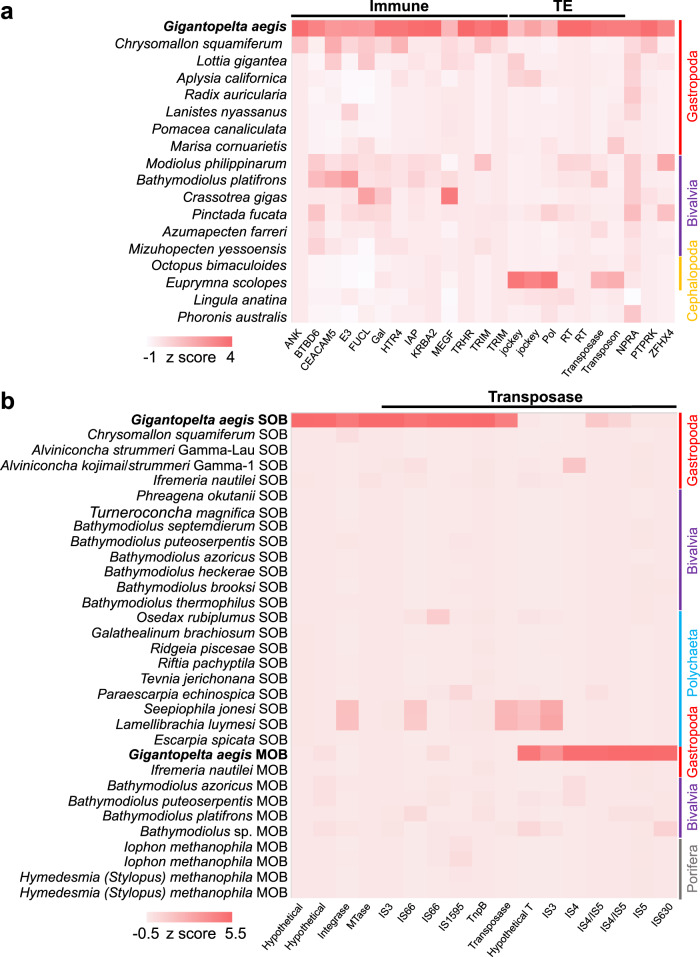
Heat maps showing the expanded gene families in *Gigantopelta aegis* and its two endosymbionts. **a** Compared with other 15 molluscan taxa (systematic affinity at the class level shown on the right side), *Phoronis australis* (Phoronida), and *Lingula anatina* (Brachiopoda) with available genomes (used in the molecular clock analysis), the gene families expanded in *G. aegis* mainly include immunity-related gene families (Immune), including ankyrin repeat protein (ANK), BTB/POZ domain-containing protein 6 (BTBD6), carcinoembryonic antigen-related cell adhesion molecule 5 (CEACAM5), E3 ubiquitin-protein ligase (E3), fucolectin (FUCL), galectin (Gal), 5-hydroxytryptamine receptor 4 (HTR4), inhibitor of apoptosis protein (IAP), KRAB-A domain-containing protein 2 (KRBA2), multiple epidermal growth factor-like domain protein (MEGF), thyrotropin-releasing hormone receptor (TRHR), and tripartite motif-containing protein (TRIM), as well as transposable elements (TE), including RNA-directed DNA polymerase from mobile element jockey (jockey), retrovirus-related Pol polyprotein from transposon (Pol), reverse transcriptase (RT), transposases, and transposons. **b** Both the sulfur-oxidising endosymbionts (SOB) and the methane-oxidising endosymbionts (MOB) exhibit transposase expansion. References are 30 symbionts in Gammaproteobacteria (see Supplementary Table 13) from invertebrate taxa living in deep-sea chemosynthetic environments. The systematic affinity of the host taxa is shown on the right side. The colour represents the normalised gene numbers. Gene family abbreviations: NPRA: atrial natriuretic peptide receptor, PTPRK: receptor-type tyrosine-protein phosphatase kappa, ZFHX4: zinc finger homeobox protein 4, Hypothetical: hypothetical protein, Integrase: integrase catalytic region, MTase: N-6 DNA methylase, IS3: IS3 family transposase, IS4: IS4 family transposase, IS4/IS5: IS4/IS5 family transposase, IS5: IS5 family transposase, IS66: IS66 family transposase, IS1595: IS1595 family transposase, IS630: IS630 family transposase, TnpB: TnpB transposase, Hypothetical T: hypothetical transposase. The colour labelling scheme of taxa: Cephalopoda: orange; Bivalvia: purple; Gastropoda: red; Polychaeta: blue; and Porifera: grey. Source data of **a** and **b** are provided as a Source Data file.

The host’s immune response is critical for symbiont infection and colonisation. In terms of the gene families shared among *G. aegis* and 18 other lophotrochozoan genomes (Supplementary Fig. 10), several immunity-related gene families were expanded in *G. aegis* (Supplementary Table 4 and Supplementary Note 4). For example, the gene families fucolectin (FUCL) and galectin (Gal) were prominently expanded in *G. aegis* (Fig. 6a); these lectins have been reported as immune-recognition molecules^34,35^. FUCLs can bind with carbohydrate recognition domains on the bacterial surface in pearl oysters^34^, and Gals can target microbial non-self glycans and act as recognition receptors in the innate immune response^35^.

**Fig. 7:**
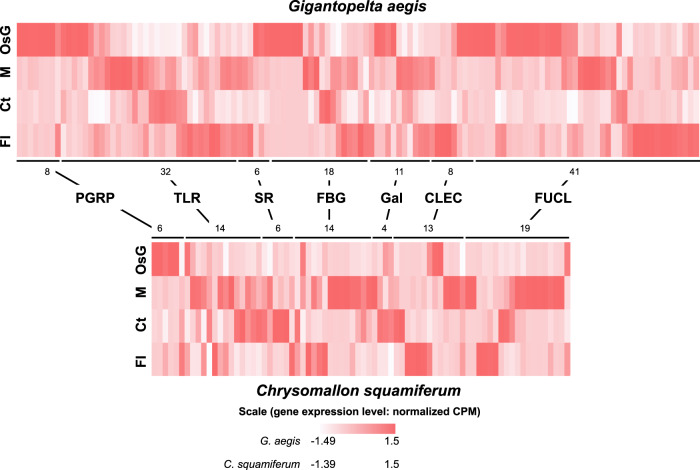
Heat maps showing gene abundances and expression levels of pattern recognition receptors in *Gigantopelta aegis* and *Chrysomallon squamiferum*. Two heat maps showing gene abundances and expression levels of pattern recognition receptors, including peptidoglycan recognition protein (PGRP), toll-like receptor (TLR), scavenger receptor (SR), fibrinogen-related protein (FBG), galectin (Gal), C-type lectin (CLEC), and fucolectin (FUCL), in *Gigantopelta aegis* (*n* = 4) and *Chrysomallon squamiferum* (*n* = 3)^17^ (OsG: oesophageal gland; M: mantle; Ct: ctenidium; FI: internal tissue of foot). The numbers near the lines indicate the gene numbers of the gene families. The colour represents the gene expression level (normalised CPM value). Red: high expression level. Light: low expression level; *n* means biologically independent animals. Source data are provided in a Source Data file.

Compared with *C. squamiferum*, *G. aegis* harbours a much greater abundance of pattern recognition receptors, including peptidoglycan recognition proteins (PGRPs), toll-like receptors (TLRs), fibrinogen-related proteins (FBGs), and C-type lectins (CLECs) (Fig. 7). These receptors are thought to help other chemosymbiotic host animals, such as siboglinid tubeworms and bathymodiolin mussels, respond to the microbes appropriately, as well as assist in the acquisition and maintenance of their symbiont populations^36^. Pattern recognition receptors highly expressed in the oesophageal glands when compared with other organs are likely related to the recognition of the endosymbionts. From gene expression patterns, all PGRPs are highly expressed in the oesophageal glands when compared with other organs in both peltospirid snails, suggesting that PGRPs play an important role in the establishment of symbiosis in both snails. A total of 26 genes in *G. aegis* and 53 genes in *C. squamiferum* involved in the lysosome pathway were highly expressed in the oesophageal gland compared with other tissues (Supplementary Figs. 11 and  12). The abundance and high expression of PGRPs and lysosome genes (Fig. 7 and Supplementary Figs. 11 and  12) may help the hosts in regulating the symbiont population^36^, possibly via the lysosomal digestion process to unlock nutrients in the symbiont biomass.

Many pattern recognition receptors other than PGRPs, particularly those belonging to the TLR gene family, are more highly expressed in the oesophageal gland of *G. aegis* than of *C. squamiferum* (Fig. 7). The TLR proteins serve as key molecular linkages between the host and bacteria in bacterial invasion and host recognition, and they are expanded in the deep-sea tubeworm *Lamellibrachia luymesi*^37^. These observations indicate that TLR proteins play an important role in symbiosis establishment across different deep-sea animals. Although *G. aegis* occupies the same habitat as *C. squamiferum* in the Longqi vent field, these additional pattern recognition receptors may have enabled *G. aegis* to host two distinct endosymbionts. The host-specific selectivity for different lineages of gammaproteobacterial endosymbionts in peltospirids is again in line with previous evidence indicating that they have independent origins of endosymbioses^14^.

There is also genomic evidence that the symbionts of *G. aegis* play a role in the invasion of the host. The peptidoglycan-associated lipoprotein (Pal), OmpA family proteins, and OmpH family proteins are widespread in environmental and symbiotic Gram-negative bacteria living in both marine and non-marine environments according to the NCBI protein database. Pal was expressed in the transcriptomes and proteomes of the *G. aegis* SOB (Supplementary Data 3, Supplementary Data 5), which can be recognised by TLR protein families of the host and further trigger an immune signalling cascade of the host in pathogenesis^38^. Furthermore, both the SOB and the MOB of *G. aegis* contained OmpA family proteins and OmpH family proteins (Supplementary Data 3 and 4), which could interact with TLR and scavenger receptors to help the bacteria invade and adapt to the intracellular environment^39^. These symbiont attributes are likely helpful in establishing an endosymbiotic lifestyle.

### Energy resources

**Fig. 8:**
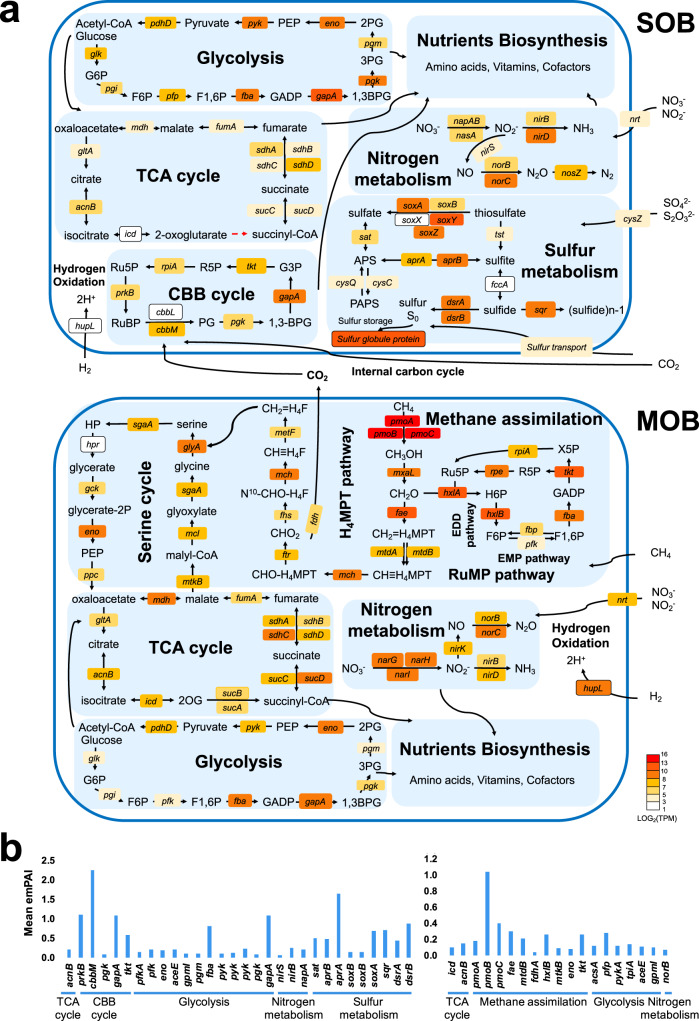
Central metabolism of the dual symbionts of *Gigantopelta aegis*. **a** Central metabolism pathways in the sulfur-oxidising endosymbiont (SOB) and the methane-oxidising endosymbiont (MOB). The boxes represent genes involved in the respective process, and they are colour coded according to the log-transformed normalised transcripts per kilobase million (TPM) value (*n* = 3) showing the gene expression level in metatranscriptome analysis (colour from red to light white represents high expression level to low expression level; dash arrow indicates that the gene is missing in the genome). **b** Protein abundances involved in the central metabolism of the SOB (left) and the MOB (right). Protein abundances are reported as mean emPAI values (*n* = 3) assessed in metaproteome analysis; *n* means biologically independent animals. The description of abbreviations are provided in Supplementary Note 8.

Previous reports showed that the concentrations of reduced components (i.e. CH_4_, sulfur substances, and hydrogen) in the endmember fluid from the Longqi field on the Southwest Indian Ridge are comparable or higher than those in the vents along the Central Indian Ridge^24,40,41^. Although methane is enriched in Longqi compared with other vents on the Central Indian Ridge^41^, further biogenic methane input from microbes living below and around the community may be sufficient to sustain the MOB. The host snail has a hypertrophied blood vascular system, which has been suggested to be an anatomical adaptation to transport essential dissolved compounds from the vent fluid together with oxygen from the surrounding seawater, to the SOB and MOB, although no transporters have been identified^14^. Among the transporters of *G. aegis* identified herein (Supplementary Data 6), those transporting oxygen (Supplementary Fig. 13) including one myoglobin, five neuroglobins, and one haemocyanin were highly expressed in the oesophageal gland, but whether they help transport hydrogen sulfide and methane remains unclear. The SOB can utilise thiosulfate, sulfite, sulfide, nitrate, nitrite, oxygen, carbon oxide, and hydrogen, while the MOB can utilise methane, nitrate, nitrite, oxygen, and hydrogen. These two symbionts form a dual symbiosis system that is highly versatile in utilising chemical substances (Fig. 8a). The central metabolism, associated gene expression levels, and protein abundance levels of the symbionts are summarised in Fig. 8.

Our genome data indicate that sulfur metabolism in the SOB of *G. aegis* (Fig. 8a) is similar to that of the SOBs of *C. squamiferum*, the provannid snails *Alviniconcha* *kojimai* and *A. strummeri* (the ‘Gamma-1’ symbiont), and the *P. echinospica* tubeworm^4,6,8^. The proteins of *soxA* (35th in proteome), *dsrB* (21th in proteome), *sqr* (34th in proteome), *aprA* (6th in proteome), *aprB* (63th in proteome), and *sat* (57th in proteome) (Fig. 8) show high expression levels, indicating their active engagement in oxidising thiosulfate, sulfite, and sulfide in the environment and in detoxifying sulfide for the holobiont.

Similar to many sulfur-oxidising endosymbionts of vent molluscs^4,5,31^, the SOB of *G. aegis* relies on the complete Calvin–Benson–Bassham (CBB) cycle to fix CO_2_ using a form II ribulose-bisphosphate carboxylase (the protein of *cbbM* gene: 2nd in proteome) recovered with the highest protein expression level and lacks a complete rTCA cycle (Fig. 8). Phosphoribulokinase (the protein of *prkB* gene: 15th in proteome), type I glyceraldehyde-3-phosphate dehydrogenase (the protein of *gapA* gene: 16th in proteome), and transketolase (the protein of *tkt* gene: 43th in proteome) play important roles in the CBB cycle for carbon fixation and exhibit high protein expression levels in the SOB (Fig. 8).

The MOB of *G. aegis* has neither a complete CBB cycle nor an rTCA cycle pathway for CO_2_ fixation. It possesses the methane monooxygenase operon (*pmoA* gene: 5th in transcriptome, 50th in proteome; *pmoB* gene: 6th in transcriptome, 1st in proteome; *pmoC* gene: 1st in transcriptome, 10th in transcriptome) within the top 1% gene expression (Fig. 8b and Supplementary Fig. 14), confirming that methane oxidation is among the most prominent metabolic processes in the MOB. In the tetrahydromethanopterin pathway, the high expression level of the formaldehyde activating enzyme (*fae* gene: 65th in transcriptome, 20th in proteome) indicates an active generation of CO_2_ via formaldehyde oxidation. The generated CO_2_ could be redirected to the CBB cycle of the SOB to form an internal carbon cycle among the symbionts, as detected in the internal symbiotic organs (trophosomes) of vent siboglinid tubeworms^42^. This phenomenon would allow the symbionts of *G. aegis* to maximise carbon efficiency within the trophosome-like oesophageal glands when in the absence of direct interaction with CO_2_-rich seawater and vent fluids.

Aerobic methanotrophs are classified into two groups based on their formaldehyde assimilation pathways: Type I (part of Gammaproteobacteria) using a ribulose monophosphate (RuMP) cycle and Type II (part of Alphaproteobacteria) using a serine cycle. Methane-oxidising symbionts usually belong to Type I and only use the RuMP cycle; examples include the MOBs of deep-sea *Bathymodiolus* mussels, snails, and sponges (refer to genomes in Supplementary Fig. 9). However, the MOB of *G. aegis* surprisingly possesses a complete serine cycle in addition to a complete RuMP cycle (Fig. 8a). This can mitigate the accumulation and toxicity of formaldehyde when formaldehyde is in excess for the RuMP pathway. In the RuMP cycle, high expression of 3-hexulose-6-phosphate synthase (*hxlA* gene: 20th in transcriptome) and 6-phospho-3-hexuloisomerase (*hxlB* gene: 25th in proteome) in the Entner–Doudoroff pathway indicate an important role for formaldehyde oxidation (Fig. 8). Furthermore, the MOB genome possesses an additional Embden–Meyerhof–Parnas pathway for glycolysis (Fig. 8a), absent in other MOBs of chemosynthetic holobionts reported to date (referred in Supplementary Fig. 9). The co-occurrence of multiple methane assimilation pathways was also present in its free-living relatives within the same family Methylomonadaceae, such as *Methylomonas methanica*, *Methylomicrobium alcaliphilum*, and *Methylomicrobium buryatense* (Fig. 8b and Supplementary Table 5). These results indicate that the *G. aegis* MOB and its free-living relatives are highly efficient and versatile in assimilating carbon sources from methane.

Sulfur-oxidising symbionts of *Bathymodiolus* mussels, provannid snails in the genera *Alviniconcha* and *Ifremeria*, siboglinid tubeworms, as well as the MOBs of *Bathymodiolus* mussels and *Ifremeria* snails possess group 1 NiFe hydrogenase (*hyaABC*)/uptake hydrogenase *hupL* (Supplementary Table 6), a key gene for hydrogen oxidation^43^, further confirming that these symbionts are capable of using hydrogen as an additional energy source^4,6,8,31^. In the present study, the *hupL* gene was also identified in the genomes of both the SOB and the MOB of *G. aegis* (Fig. 8 and Supplementary Data 3 and 4). The *hupL* of both *G. aegis* symbionts were expressed in the transcriptome but at lower abundance compared with genes responsible for sulfur oxidation and methane oxidation, and they were not found in the metaproteome data possibly due to low abundance. Nevertheless, these results still indicate that both the SOB and the MOB of *G. aegis* can utilise hydrogen. This study further corroborate the existing hypothesis that the capability of utilising hydrogen for energy is widespread in the symbionts of macrobenthos living in deep-sea chemosynthesis-based ecosystems^43^.

### Respiration

Both the SOB and the MOB housed in the oesophageal gland of *G. aegis* can use oxygen and nitrate as electron acceptors for respiration. They are both aerobic and possess succinate dehydrogenase, cytochrome *c* reductase, and cytochrome oxidase to utilise oxygen (Supplementary Data 3 and 4). Their genomes also encode genes for nitrate reduction, such as nitrate reductase (*napAB* and *nasA* in the SOB and *narGHI* in the MOB), nitrite reductase (*nirBDS* in the SOB and *nirBDK* in the MOB), and nitric oxide reductase (*norBC* in both symbionts) (Fig. 8). The symbionts of *C. squamiferum*, deep-sea siboglinid tubeworms, bathymodilin mussels, vesicomyid clams, provannid snails, and sponges also have the capacity for nitrate respiration (Supplementary Table 7). As the *G. aegis* symbionts rely on the host’s blood vessels for oxygen supply while input from the hydrothermal vent fluctuates due to the constant interplay between the oxygen-poor vent fluid and the oxygen-rich seawater, there may be periods when the oxygen supply runs low in the oesophageal gland. Haemocyanin, haemoglobin, and globin-like genes which play a role in oxygen binding and transport have lower gene expression in the oesophageal gland and higher expression in the foot and mantle, while five sialin genes used for transporting nitrate were highly expressed in the oesophageal gland (Supplementary Fig. 13). Therefore, these symbionts may have developed abilities to avoid oxygen competition with the host, especially under potential periodic hypoxic conditions.

### Holobiont nutritional interdependence

Unlike the SOB of *C. squamiferum* which has a complete TCA cycle^4^, the SOB of *G. aegis* lacks the 2-oxoglutarate dehydrogenase gene in the TCA cycle while their homologous genes are present and highly expressed in the MOB. The SOB of *G. aegis* has a C4-dicarboxylate TRAP transport system (DstPQM) that allows the uptake of four-carbon compounds involved in the TCA cycle^8^, indicating that the SOB likely replenishes the missing intermediates of the TCA cycle from one of the other symbiotic partners.

Both the *G. aegis* and *C. squamiferum* host genomes lack capability to biosynthesise six amino acids (isoleucine, valine, leucine, lysine, histidine, and tryptophan), six vitamins (vitamin B_1_, vitamin B_2_, vitamin B_5_, vitamin B_7_, vitamin B_9_, and vitamin B_12_), and coenzyme A (Fig. 5). Other molluscs with high-quality genomes available, except the shallow water mussel *Modiolus philippinarum*, exhibited the same biosynthetic capacity of amino acids (Fig. 5). Supplementary Fig. 15 shows the phylogenetic relationship of the symbionts and their free-living relatives with sequenced genomes. The nutrient metabolism of both the SOB and the MOB are likely to be different from that of their free-living relatives (Fig. 5).

Most nutrients that the hosts cannot biosynthesise themselves, except vitamin B_5_ and coenzyme A, can be obtained from their SOBs through the host’s lysis of symbiont cells, as indicated by the high expression of lysosome genes in the oesophageal glands (Supplementary Figs. 11 and 12). The MOB of *G. aegis* has a complete pathway to synthesise vitamin B_5_ and coenzyme A, whereas the SOBs of the two endosymbiotic peltospirids lack a key gene, namely, 2-dehydropantoate 2-reductase (*panE*), in the biosynthesis of pantothenate and coenzyme A (Supplementary Fig. 16). Although the gene may be missing in the technical genome assembly or gene prediction, considering the high-quality of these two assembled genomes with high sequencing coverage of long reads, it is highly likely that this gene is truly missing from the symbiont genomes. Therefore, we speculate two possibilities: (1) the SOB, particularly of *C. squamiferum*, has a replacement of 2-dehydropantoate 2-reductase or replenishment for the biosynthesis of pantothenate and coenzyme A as observed in a pea amphid holobiont^44^, or (2) in the *G. aegis* holobiont, the MOB serves as the major source for providing pantothenate and coenzyme A to both the SOB and the host. In return, the host can provide its symbionts with vitamin B_3_ and vitamin B_6_ through the symbionts’ ATP-binding cassette transporters of amino acids. These host-supplied nutrients are critical for fulfilling the nutritional demands of the endosymbionts housed in an internal organ that lacks direct contact with the outside world.

### Transposase expansion in three partners of the *G. aegis* holobiont

Enrichment of transposable elements (TEs) provides beneficial genomic plasticity for adaptation to an intracellular, symbiotic lifestyle by regulating nearby genes^45^. In the results from the gene family analysis, the expansion of TEs was found in all three parties comprising the *G. aegis* holobiont (Fig. 6). For the host snail, these expansions corresponded to 342 transposase genes (1.6% of total host genes) and 510 DNA transposons (2.4% of total host genes); for the SOB, these expansions corresponded to 704 transposase genes (12.8% of total SOB genes); and for the MOB, these expansions corresponded to 345 transposase genes (11.1% of total MOB genes). Previous studies^45,46^ reported that transposase genes were particularly enriched in bacterial lineages that have recently transitioned to a host-associated lifestyle, indicating that enrichment of transposase genes may have a beneficial effect for the symbionts of *G. aegis* in becoming endosymbionts. Moreover, numerous transposase genes were unusually inserted into the flagellar operons and chemotaxis operons that play important roles in the host infection of both the SOB and the MOB of *G. aegis*. These genes may contribute to the regulation of the symbionts’ motility for infection and colonisation in the host environment. To our knowledge, the expansion of transposase genes occurring across multiple symbiotic partners within the same holobiont has not been reported in any marine invertebrate holobiont.

Overall, we revealed that the deep-sea vent peltospirid snail *G. aegis* exhibits a dual symbiotic lifestyle, housing a more abundant sulfur-oxidising Gammaproteobacteria and a less abundant methane-oxidising Gammaproteobacteria (Type I methanotroph). Furthermore, we provide a high-quality hologenome including a chromosome-level assembly for the snail host and an in-depth analysis of the genomic interdependencies among the ‘triptych’ of symbiotic partners in this holobiont, finding an intimate mutualistic relationship with complementarity in nutrition and metabolic co-dependency. The two endosymbionts live in bacteriocytes of the oesophageal gland, an organ lacking direct interaction with the ambient environment. Both symbionts and the host are highly versatile with regard to the transportation and utilisation of chemical energy, increasing the efficiency of carbon fixation by forming an internal carbon cycle between the two symbionts. Although *G. aegis* and another chemosymbiotic peltospirid snail, the Scaly-foot Snail *C. squamiferum*, occupy the same habitat in the Longqi vent field, *C. squamiferum* has a single sulfur-oxidising symbiont. Our data support previous findings that the two peltospirid snails evolved symbiosis independently and convergently, but their phylogenetically distinct sulfur-oxidising endosymbionts have the same capabilities in the biosynthesis of specific nutrients. As such, nutrient biosynthesis capacity may be a key constraint in the selection of symbionts by these peltospirids.

## Methods

### Deep-sea sampling

*Gigantopelta aegis* snails were collected by the manned submersible HOV *Jiaolong* on-board the R/V *Xiangyanghong 9* cruise 35II from the Longqi hydrothermal vent field (37.7839°S, 49.6502°E; 2761 m depth) on the Southwest Indian Ridge in January 2015. Snails were immediately flash-frozen in liquid nitrogen once recovered on the ship. They were then transferred to −80 °C until DNA and RNA extraction. Specimens of *G. aegis* for Hi-C sequencing and FISH experiments were also collected from Longqi (‘Tiamat’ chimney) during Leg 3 of the COMRA R/V *Dayang Yihao* expedition 52 in April 2019. Tissue from the foot was immediately dissected from one individual, cut up, washed in phosphate-buffered saline buffer, and stored at −80 °C until Hi-C library preparation. The oesophageal gland tissue was dissected from the same individual and fixed in 4% paraformaldehyde overnight, followed by dehydration through an ethanol series (20%, 40%, 60%, and 80%) for 15 min each and stored at −80 °C until FISH experiments. Samples for TEM were immediately fixed in 10% buffered formalin after recovery on board of the research vessel. The identity of the specimens was confirmed by both mitochondrial *COI* sequences and morphology. Detailed information on how each individual was used is provided in Supplementary Table 8.

### TEM

The oesophageal gland tissue was dehydrated using a series of graded acetone and then transferred into Epon resin (Sigma-Aldrich) for embedding. An ultramicrotome (Reichert Ultracut S, Leica) was used to slice ultrathin (70 nm) sections that were used for staining in 2% aqueous uranyl acetate with lead stain solution (0.3% lead acetate and 0.3% lead nitrate, Sigma-Aldrich). The presence of intracellular symbionts was confirmed using a Tecnai 20 Transmission Electron Microscopy (FEI) at an acceleration voltage of 120 kV. For more detailed methods for thin-section preparation, see ref. ^14^.

### FISH

Oesophageal gland tissues of *G. aegis* were dehydrated in 100% ethanol and embedded in Shandon Cryomatrix Frozen Embedding Medium (6769006; Thermo Fisher Scientific). After full embedding, they were quickly frozen in dry ice. Cryostat sections with thickness of 10 μm (CryoStar NX70; Thermo Fisher Scientific) were cut and mounted on glass slides. Two 16S rRNA-based probes were designed to target the endosymbionts specifically. The design of FISH probes for the SOB and the MOB of *G. aegis* was based on their respective 16S rRNA gene sequences. To ensure that the 20 bp probe was unique and specific, the 16S rRNA gene sequence was searched against online standard databases of nucleotide collection in NCBI. The alignments among the 16S rRNA genes of symbionts and their five best hit sequences with highest sequence similarity were manually checked. The aligned region with the largest difference was manually chosen for probe designs. The uniqueness of the probe sequences was further confirmed by searching against the NCBI nt databases. After confirming the specificity of the 20 bp sequence regions of the SOB and the MOB, 20 bp oligonucleotide probes with 100% match to the regions were synthesised, and their 3′ ends were labelled with fluorescent dye Cy3 and Cy5, respectively. The Cy3-labelled SOB1 probe (5′-AGCATATTAAACTTGTACCC-3′) was used to target the sulfur-oxidising endosymbiont, and the Cy5-labelled MOB1 probe (5′-CGTGTGTTTTCCTCCCTTCT-3′) was used to bind the methane-oxidising endosymbiont (Supplementary Table 9). The sections were rehydrated in a decreasing ethanol series (95%, 80%, and 70%) for 15 min each and hybridised at 46 °C with 50 ng/ml of each probe in a hybridisation buffer (0.9 M NaCl, 0.02 M Tris-HCl, 0.01% sodium dodecyl sulfate, and 20% formamide) for 3 h. The slides were washed in a wash buffer (0.1 M NaCl, 0.02 M Tris-HCl, 0.01% sodium dodecyl sulfate, and 5 mM EDTA) at 48 °C for 15 min. Two drops of 4′,6-diamidino-2-phenylindole (DAPI) were added on each slide, followed by incubation at room temperature for 3 min. After washing and air drying, the slides were mounted by coverslips with SlowFade™ Diamond Antifade Mountant medium (Invitrogen, Carlsbad, CA, USA). Images were obtained using a confocal microscope (Leica Microsystems, Wetzlar, Germany) with a 3D model and post-processed by LAS X software version 3.0.13 (Leica Microsystems, Wetzlar, Germany).

### DNA extraction and Illumina sequencing of *G. aegis* hologenome

The MagAttract High-Molecular-Weight DNA Kit (QIAGEN, Hilden, Netherlands) was used to extract high-molecular-weight genomic DNA from the oesophageal gland and the foot separately for sequencing of the genome of the symbionts and the host, respectively, following manufacturer’s protocols. The extracted DNA was further purified by a Genomic DNA Clean & Concentrator^TM^-10 kit (ZYMO Research, Irvine, CA, USA). The DNA was finally eluted in 10 mM Tris-HCl buffer (pH 8.5). The quality of the genomic DNA was evaluated using a BioDrop μLITE (BioDrop, Cambridge, UK) with the OD 260/280 of 1.8 and the OD 260/230 of 2.0–2.2. The DNA concentration was assessed using a Qubit^TM^ 3 Fluorometer (Thermo Fisher Scientific, Singapore). The sizes of DNA fragments were assessed by pulsed-field gel electrophoresis. Approximately 1 µg of DNA of each tissue, including foot and oesophageal gland, was used for the short-insert library (350 and 500 bp) in an Illumina NovaSeq 6000 platform to generate 171 and 49 Gb paired-end reads with a length of 150 bp, respectively.

### Oxford Nanopore Technologies (ONT) library preparation and MinION sequencing

The DNA of the oesophageal gland was used to prepare the long-read library (Oxford Nanopore MinION, UK) for genome sequencing of endosymbionts. To construct the MinION library, a NEBNext Ultra II End-Repair/dA-tailing Module (NEBE7546) kit was used to perform DNA repair and end-prep. A Ligation Sequencing Kit (SQK-LSK109) was used to perform adaptor ligation and clean-up with the large DNA fragments. A Flow Cell Priming Kit (EXP-FLP001) was used to prepare the priming buffer to prime the MinION flow cells (FLO-MIN106D R9 version). The Library Loading Bead Kit R9 version (EXP-LLB001) was used to assist in loading the libraries into the flow cells. Sequencing began immediately in the MinION^TM^ portable single-molecule nanopore sequencer (Oxford Nanopore Technologies Limited, UK) using the software MinKNOW version 3.1.8 (Oxford Nanopore Technologies Limited, UK). The read event data were base-called by Oxford Nanopore basecaller Guppy version 2.1.3 (ref. ^47^) with default settings.

#### Single-molecule real-time library construction and PacBio sequel sequencing

For genome sequencing of the host animal, the purified DNA of the foot from one individual was utilised to construct PacBio single-molecule real-time (SMRT) library (Pacific Biosciences, USA). BluePippin (Sage Science, USA) was utilised to select long DNA fragments with sizes between 8 kb and 50 kb for PacBio library construction. Universal hairpin adaptors were used to ligate the DNA fragments. The adaptor dimers and hairpin dimers were both removed using magnetic beads in PacBio’s MagBead kit. Exonucleases were further used to remove the failed ligation DNA fragments. The purified library was sequenced by binding the sequencing polymerase in the SMRT sequencing cells and generating long subreads with a length distribution (Supplementary Fig. 17).

### Hi-C sequencing

To construct the Hi-C library, frozen foot tissue of *G. aegis* was thawed slowly on ice and suspended in 45 ml of 37% formaldehyde in serum-free Dulbecco’s modified Eagle’s medium for chromatin cross-linking. After incubation at room temperature for 5 min, glycine was added to quench formaldehyde, followed by incubation at room temperature for another 5 min and then on ice for over 15 min. The cells were further lysed in pre-chilled lysis buffer (10 mM NaCl, 0.2% IGEPAL CA-630, 10 mM Tris-HCl, and 1× protease inhibitor solution) using a Dounce homogeniser. The chromatin was digested by Mbo I restriction enzyme, marked with biotin and ligated^48^. The Hi-C library was sequenced on a NovaSeq 6000 platform (Illumina, USA) and generated reads with a length of 150 bp. The genome sequencing strategy of the host is summarised in Supplementary Table 10.

### RNA extraction, metatranscriptome sequencing, and eukaryotic transcriptome sequencing

The total RNA of oesophageal glands dissected from three individuals of *G. aegis* were separately extracted using the TRIzol reagent (Invitrogen, Carlsbad, CA, USA). The quality and quantity of RNA were checked by agarose gel electrophoresis and BioDrop μLITE (BioDrop, Cambridge, UK), respectively. To enrich the mRNA of endosymbionts, the rRNA of both eukaryote and bacteria was removed by both the Ribo-Zero^TM^ Magnetic Kit (Human/Mouse/Rat) (Epicenter, USA) and the Ribo-Zero^TM^ Magnetic Kit (Bacteria) (Epicenter, USA) following the manufacturer’s protocols. The remaining RNA was used to construct cDNA libraries for metatranscriptome sequencing. The sequencing data are shown in Supplementary Table 11.

Four *G. aegis* individuals were dissected to obtain different organs (foot, mantle, ctenidium, oesophageal gland, etc.) for eukaryotic transcriptome sequencing (Supplementary Table 11). Total RNA of these samples was separately extracted using TRIzol reagent (Invitrogen, Carlsbad, CA, USA) following the manufacturer’s protocol. mRNA from each host tissue was enriched by Oligo-dT probes and further transferred to cDNA for eukaryotic library construction. All cDNA libraries were sequenced in a NovaSeq 6000 Illumina platform to generate paired-end reads with a length of 150 bp.

### Genome assembly and binning of symbionts

Adaptors and low-quality bases of raw Illumina sequencing reads of the oesophageal gland were trimmed using Trimmomatic version 0.36 (ref. ^49^) with the settings ILLUMINACLIP: Truseq3-PE-2.fa. To obtain the genome of symbionts, clean Illumina reads from the oesophageal gland were used for initial assembly in SPAdes version 3.13.0 (ref. ^50^) with the --meta setting as well as a series of kmers of 51, 61, 71, 81, and 91 bp. Illumina short reads belonging to the symbionts were grouped by genome binning based on the sequencing coverage and the GC content of the initial contigs^51,52^. The coverage of each contig was calculated using coverage_sum.pl and coverage_length.pl, implemented in Albertsen et al.^51^ and Tian et al.^52^, based on the mapping depth assessed via Bowtie2 version 2.3.5 (ref. ^53^) and SAMtools version 1.6 (ref. ^54^). The GC content of each contig was calculated by using calc.gc.pl^51,52^. The script metagenome.workflow.modified.R^51,52^ was used to extract the symbiont genomes. The grouped Illumina short reads and all ONT sequencing reads were reassembled together by Unicycler version 0.4.7 (ref. ^55^). The assembled contigs were grouped again via genome binning. Each group of contigs was further used in genome scaffolding by SSPACE-LongRead version 1.1 (ref. ^56^) with the ONT reads and mapped PacBio reads that were over 10 kb. CheckM version 1.0.13 (ref. ^57^) was used to assess the completeness and the contamination of the assembled bacterial genomes.

### Assembly and scaffolding of the host genome

Several assembly pipelines were used for genome assembly of the host, and the best contig assembly result was generated by Canu correction + wtdbg2 (Supplementary Table 12). PacBio subreads over 4 kb were corrected by Canu version 1.7.1 (ref. ^58^) with settings of genomeSize = 1.27 Gb, corMhapSensitivity = normal, corMinCoverage = 0, corMaxEvidenceErate = 0.15, correctedErrorRate = 0.065, minReadLength = 8000 and then used in genome assembly using wtdbg2 version 2.1 (ref. ^59^) with settings: -e 2 --tidy-reads 5000 -S 1 -k 15 -p 0 --rescue-low-cov-edges --aln-noskip. To improve the accuracy of the initially assembled contigs, two polishing rounds using Racon version 1.3.1 (ref. ^60^) were performed and followed by an additional round using Pilon version 1.22 (ref. ^61^) with Illumina short reads of the foot tissue mapped by Bowtie2 version 2.3.5 (ref. ^53^) in the --sensitive mode. Bacterial contamination in the host genome assembly was further filtered using MaxBin version 2.2.5 (ref. ^62^).

Raw Hi-C sequencing reads were first trimmed by Trimmomatic version 0.36 (ref. ^49^). To remove the invalid pairs of Hi-C reads without effective ligation, the reads were mapped to the corrected assembled contigs using Bowtie2 version 2.3.5 (ref. ^53^), and Hi-C contact maps were generated based on the mapped reads using HiC-Pro version 2.11.1 (ref. ^63^). Juicer version 1.5 (ref. ^64^) was used to filter and deduplicate. The remaining valid reads were used for contig scaffolding using the 3D de novo assembly (3D-DNA) pipeline version 180114 (ref. ^65^) in diploid mode. The scaffolds were manually corrected based on the Hi-C contact maps (Supplementary Fig. 18). The continuity and completeness of the assembled genome were evaluated with assemblathon_stats.pl (https://github.com/ucdavis-bioinformatics/assemblathon2-analysis/blob/master/assemblathon_stats.pl) and BUSCO version 3.0.2 (ref. ^66^) using the metazoa_odb10 database, respectively.

### Genes prediction and functional annotation

The coding sequences and proteins of symbionts genomes were predicted by Prodigal version 2.6.3 (ref. ^67^) with default settings. The host genome was first hard-masked in the repetitive regions and then trained with the boundary of the introns and exons to predict coding genes of *G. aegis* (for details see Supplementary Note 2).

Repeats in the *G. aegis* genome were de novo identified and classified using RepeatModeler version 1.0.11 (http://www.repeatmasker.org/RepeatModeler/) pipeline and soft-masked using RepeatMasker version 4.0.8 (http://www.repeatmasker.org/RMDownload.html) with the parameter -xsmall (for details see Supplementary Note 2).

To predict the gene models, we ran two rounds of the MAKER pipeline version 2.31.10 (ref. ^68^) on the soft-masked genome. To guide the prediction, we used de novo assembled transcripts, Metazoan protein sequences downloaded from the Swiss-Prot database, and protein sequences of *C. squamiferum*^22^ (for details see Supplementary Note 2).

The predicted protein sequences were searched against the NCBI Non-Redundant (NR) database using BLASTp with an *E*-value cutoff of 1e−5. The sequences were also used to search the Kyoto Encyclopedia of Genes and Genomes (KEGG) database via KEGG Automatic Annotation Server using bi-directional best hit methods of BLASTp version 2.2.31+ (ref. ^69^). The Pfam database^70^ with hmmscan version 3.2.1 (http://hmmer.org/) was used to identify the functional domains of proteins. The protein sequences of the host were further searched against the EuKaryotic Orthologous Groups database. The protein sequences of symbionts were annotated against the functional COG using eggNOG-mapper v2 (ref. ^71^). The EcoCyc database^72^ in Pathway Tools^73^ was additionally used to check the absence or presence of the nutrient biosynthesis pathway in the symbionts.

### Draft genome of *D. subfuscus*

*Dracogyra subfuscus* were collected from Longqi (‘Tiamat’ chimney) during Leg 3 of the COMRA R/V *Dayang Yihao* expedition 52 in April 2019, fixed in RNAlater and transferred to −80 °C freezer until use. The whole tissue of one individual of *D. subfuscus* was used for DNA extraction. DNA extraction, assessment of DNA quality, Illumina short-insert (350 and 500 bp) library construction, raw read clean, and genome binning were performed following the same methods applied to *G. aegis*. The clean Illumina reads of *D. subfuscus* were used for genome assembly by Platanus version 1.2.4 (ref. ^74^) with settings of -u 0.3. The coding region of the genome was predicted by MAKER version 3.01.03 (ref. ^68^) with the Mollusca proteins (NCBI, July 2020) and the transcripts of *G. aegis* and *C. squamiferum*. Only the genes with a protein length larger than 50 amino acids were kept for subsequent analysis. Given that the genome was fragmented, the assembled draft genome of *D. subfuscus* was only used in phylogenomic analysis and checking for microbes.

### Gene family and phylogenomic analysis

To perform the gene family analysis of *G. aegis*, Orthofinder version 2.3.3 (ref. ^75^) was used to identify the orthologous groups shared between proteins of *G. aegis* and those of other molluscan genomes (see Supplementary Note 3). For TEs, only transposase genes and DNA transposon genes were analysed in the gene family analysis. These genes in other molluscan genomes used in comparisons were found by identifying the orthologous groups shared between proteins of *G. aegis* and those of other molluscan genomes. Only single-copy orthologous groups including at least 12 species were used to construct the phylogenetic tree. The missing genes were replaced by question marks in the alignments. The protein sequences of each gene were aligned separately using MUSCLE version 3.8.31 (ref. ^76^) and concatenated for phylogenetic analysis using IQ-TREE multicore version 1.6.10 (ref. ^77^) with settings of LG+I+G4+F, 1000 ultrafast bootstraps and partitions. The software MCMCtree^78^ was used to yield the time-calibrated tree by calibrating the phylogenetic tree with seven fossil records and geographic events (see Supplementary Note 3).

Both CAFE 3 (ref. ^79^) and Fisher’s Exact Test were used for the gene family analysis. The time-calibrated tree and gene family numbers shared by these species were used for gene family analysis in CAFE 3 (ref. ^79^). The Fisher’s Exact Test was also used for gene family analysis based on the gene family numbers of *G. aegis* and the average gene family numbers of other species included. Only the gene families with an FDR-corrected *P* value smaller than 0.05 were considered expanded or contracted.

To explore the phylogenetic relationship of the symbionts with their relatives, phylogenetic trees based on the 16S rRNA gene alignment generated by MUSCLE version 3.8.31 (ref. ^76^) were constructed for both the SOB and the MOB of *G. aegis* using IQ-TREE multicore version 1.6.10 (ref. ^77^) with -m MFP and 1000 ultrafast bootstraps, respectively. To compare the essential metabolism pathway (i.e. nutrient biosynthesis and essential carbon metabolism) with their free-living relatives with available genomes, their genomes were used for phylogenetic and metabolic pathway analyses. To explore the phylogenetic relationship of the symbionts, 30 genomes of bacterial symbionts belonging to Gammaproteobacteria from deep-sea invertebrate taxa were included (for details of referred symbionts, see Supplementary Table 13). Same pipelines of the orthologue cluster of *G. aegis* were applied in the symbiont analyses. MUSCLE version 3.8.31 (ref. ^76^) was used for protein alignments. The alignments of single-copy orthologues were concatenated for subsequent phylogeny analysis. The IQ-TREE multicore version 1.6.10 (ref. ^77^) with settings of LG+I+G4+F and 1000 ultrafast bootstraps were used to perform the phylogeny analysis of the symbionts.

### Gene expression analysis of the holobiont

The coding DNA sequences of the predicted genes were used as references to assess gene expression. Kallisto version 0.45.1 (ref. ^80^) was used to calculate transcripts per million (TPM) value based on the mapped reads count for each gene. In symbionts, the TPM values were directly used for gene expression comparison. For the host, the highly expressed genes in the oesophageal gland were identified by differential expression analysis versus foot, ctenidium, and mantle (*n* = 4) using edgeR^81^ based on the reads counts. If genes had a fold change of reads counts larger than two and a significant FDR *P* value (<0.05) generated from edgeR^81^, they were designated as highly expressed genes. To compare the gene expression of *G. aegis* and *C. squamiferum*, the same analysis pipeline was applied to the transcriptome sequencing data of foot tissue, ctenidium, mantle, and oesophageal gland from *C. squamiferum*^22^. The heat maps were plotted based on the counts per million (CPM) value normalised by the STANDARDIZE function complemented in Excel.

### Synteny analysis

Pairwise synteny blocks were searched using LAST version 1080 (ref. ^82^). The hits were used to detect collinear blocks and synteny shared among *G. aegis* (15 chromosomes), *C. squamiferum* (15 chromosomes), *P. canaliculata* (14 chromosomes), and *M. yessoensis* (19 chromosomes) with chromosomal scale assemblies by MCScan (python version: https://github.com/tanghaibao/jcvi).

### Metaproteomics

The oesophageal glands from three *G. aegis* individuals were used for protein extraction using the methanol-chloroform method^83^. SDS-PAGE gel was used to separate different sizes (ranging from 10 kDa to 150 kDa) of ~30 µg of extracted protein from each sample and stained by colloidal Coomassie blue. The peptide for LC-MS/MS was obtained through protein reduction, alkylation and digestion, peptide extraction, and further drying. Dionex UltiMate 3000 RSLCnano coupled with an Orbitrap Fusion Lumos Mass Spectrometer (Thermo Fisher) was utilised to analyse each protein fraction. The search database contains the protein sequences predicted from the genome and the corresponding reversed sequences (decoy) of both *G. aegis* and its two endosymbionts. Mascot version 2.3.0 was used to identify and quantify the protein via the raw mass spectrometry data. Proteins were identified with the assigned peptides’ identification confidence level over 0.95 and false discovery rate of less than 2.5% (see Supplementary Note 5).

### Ethics declarations

As the species used (*G. aegis* and *D. subfuscus*) are invertebrate gastropod molluscs, no ethical approval or guidance was required. No cephalopod samples were used in this study. Research cruises and their collecting activities were authorised and approved by the China Ocean Mineral Resources Research and Development Association.

### Reporting summary

Further information on research design is available in the Nature Research Reporting Summary linked to this article.

## Supplementary information

Supplementary Information

Peer Review File

Reporting Summary

Description of Additional Supplementary Files

Supplementary Data 1

Supplementary Data 2

Supplementary Data 3

Supplementary Data 4

Supplementary Data 5

Supplementary Data 6

## Data Availability

All sequencing data and assembly data of *G. aegis* and its two symbionts were deposited to the National Centre for Biotechnology Information (NCBI) database under BioProject PRJNA612619. Detailed data information of *G. aegis* holobiont were provided in Supplementary Tables 8 and 11. All sequencing data and assembly data of *D. subfuscus* were also deposited to the NCBI database under BioProject PRJNA680542. Assembly data and genome annotation data of both *G. aegis* and *D. subfuscus* are available in figshare (10.6084/m9.figshare.13317932 and 10.6084/m9.figshare.13317932.v1)^84^. The genome assembly and predicted gene models of *G. aegis* can also be found at MolluscDB^85^ (http://mgbase.qnlm.ac/page/download/download). The metaproteomic data of *G. aegis* are available in PRIDE via ProteomeXchange under the identifier PXD022852. Publicly available datasets used in the study include the following: NCBI NR database (https://www.ncbi.nlm.nih.gov/refseq/), KEGG (https://www.genome.jp/kegg/), Repbase (https://www.girinst.org/repbase/), EuKaryotic Orthologous Groups (KOG) database (https://mycocosm.jgi.doe.gov/help/kogbrowser.jsf), Clusters of Orthologous Groups (COG) database (https://www.ncbi.nlm.nih.gov/COG/), Pfam database (https://pfam.xfam.org), RAST server (http://rast.theseed.org/), SwissProt database via UniProt (https://www.uniprot.org/), and the ecocyc database (https://ecocyc.org). Source data are provided with this paper.

## Source data

Source Data

## Code availability

All computer commands, codes, and intermediate files for the bioinformatics analyses are based on the available software listed in the ‘Methods’ section, which are provided at https://github.com/ylan3740/Commands-for-hologenomic-analyses under the GNU General Public License v3.0.
