# Supplementary material for: Hologenome analysis reveals dual symbiosis in the deep-sea hydrothermal vent snail Gigantopelta aegis
Source: Nat Commun. 2021 Feb 19;12:1165. doi: 10.1038/s41467-021-21450-7 (PMC7895826; doi:10.1038/s41467-021-21450-7)
Supplement: Supplementary file 1 — Supplementary Information [file 41467_2021_21450_MOESM1_ESM.pdf]

Supplementary Information for:

Hologenome analysis reveals dual symbiosis in the deep-sea hydrothermal vent snail *Gigantopelta aegis*

Yi Lan, Jin Sun, Chong Chen, Yanan Sun, Yadong Zhou, Yi Yang, Weipeng Zhang, Runsheng Li, Kun Zhou, Wai Chuen Wong, Yick Hang Kwan, Aifang Cheng, Salim Bougouffa, Cindy Lee Van Dover, Jian-Wen Qiu, Pei-Yuan Qian

Supplementary Notes

Supplementary Note 1: Host Genome Assembly 6
1.1 Genome Survey...6
1.2 Genome Assembly Pipelines 6
1.3 Mitochondria Genome Assembly and Characteristics...7
Supplementary Note 2: Host Genome Annotation 7
2.1 Repeats Annotation..8
2.2 Genes Prediction of the Host 8
2.3 Genome Assembly and Gene Prediction of Symbionts...8
Supplementary Note 3: Molecular Clock Analysis 9
Supplementary Note 4: Gene Family Expansion 9
Supplementary Note 5: Proteomic Approach 10
Supplementary Note 6: Synteny 10
Supplementary Note 7: *Dracogyra subfuscus* Genome..11
Supplementary Note 8: Abbreviation List..53
Supplementary References...56

Supplementary Figures

Supplementary Figure 1 Composition of different genomic components of *Gigantopelta aegis* and *Chrysomallon squamiferum*. Outer ring: *G. aegis*; Inner ring: *C. squamiferum*. Exon: blue; intron: orange; repeat region: grey; intergenic region (exclude repeats): yellow. Source data are provided in a Source Data file. 12
Supplementary Figure 2 Transmission electron microscopy (TEM) and fluorescence *in situ* hybridisation (FISH) images of the oesophageal gland from *Gigantopelta aegis*. The TEM images of a. an entire bacteriocyte cell housing intracellular endosymbiont (scale bar: 5 μm), b. endosymbionts showing two distinct morphological types (scale bar: 2 μm), and c. one endosymbiont showing intracellular stacked membranes (scale bar: 500 nm). white outline: bacteriocyte cell membrane; s: sulphur-oxidising symbiont; m: methane-oxidising symbiont. FISH images of d. host nuclear DNA, e. sulphur-oxidising bacteria (SOB), f. methane-oxidising bacteria (MOB), and g. the merged signal of d., e., and f. on transverse sections of

oesophageal gland from *Gigantopelta aegis* (scale bar: 50 μm). FISH experiments were performed with specific 16S rRNA probes for SOB and MOB. FISH image of h. Negative control: DNA (DAPI staining) and NON338 probe[56] The signal of NON338 is bare. i. Positive control: DNA (DAPI staining) and universal EUB338 probe[57]: green signal. Colour: DNA (DAPI staining): blue; SOB (Cy3): green; MOB (Cy5): red. TEM experiments were applied on 3 individuals with more than 3 thin sections each. FISH experiments were repeated independently for twice and at least 10 sections of the samples were used for each time. These experiments were repeated with similar results. 13
Supplementary Figure 3 A phylogenetic tree of *Gigantopelta aegis* MOB and its closest relatives based on 16S rRNA gene. 15
Supplementary Figure 4 A phylogenetic tree of *Gigantopelta aegis* sulphur-oxidising symbiont (SOB) and its closest relatives based on 16S rRNA gene.......................................16
Supplementary Figure 5 Genome binning of the initially assembled contigs from the oesophageal gland of *Gigantopelta aegis*. Two dominant bacteria had significantly higher coverage than the host. Each dot represents a contig. SOB: sulphur-oxidising bacteria; MOB: methane-oxidising bacteria. The colour labelling scheme of phylum: red: proteobacteria; blue: opisthokonta; green: viridiplantae...17
Supplementary Figure 6 The *Hox* clusters shared same gene order between *Gigantopelta aegis* and *Chrysomallon squamiferum*. The description of the protein names are provided in the Supplementary Note 8 Abbreviation List. 18
Supplementary Figure 7 The 13 protein-coding genes of mitochondrial genomes shared same gene order between *Gigantopelta aegis* and *Chrysomallon squamiferum*. The description of the gene names are provided in the Supplementary Note 8 Abbreviation List. 19
Supplementary Figure 8 Genome binning of the initially assembled contigs of *Dracogyra subfuscus*. Each dot represents a contig. The colour labelling scheme of phylum: red: unclassified organism; blue: proteobacteria; green: bacteria named after planctomycetes, verrucomicrobia, and chlamydiae (PVC) group; purple: opisthokonta; orange: viridiplantae; and yellow: terrabacteria...20
Supplementary Figure 9 Phylogenetic reconstruction of 32 symbionts in Gammaproteobacteria from invertebrate taxa living in deep-sea chemosynthetic environments. A total of 424 single-copy orthologs were used to construct the tree. The phylogenetic tree includes two major lineages of the sulphur-oxidising bacteria (green) and the methane-oxidising bacteria (red). The information of 32 symbionts is provided in Supplementary Table 13..21
Supplementary Figure 10 A heat map of shared gene family numbers of *Gigantopelta aegis* (Gae) and other references. Apl: *Aplysia californica* [GenBank No. GCA_000002075]; Bpl: *Bathymodiolus platifrons*; Afa: *Azumapecten farreri*; Cgi: *Crassotrea gigas*; Csq: *Chrysomallon squamiferum*; Eup: *Euprymna scolopes*; Hal: *Haliotis rufescens*; Lan: *Lanistes nyassanus*; Lin: *Lingula anatina*; Lot: *Lottia gigantea*; Mco: *Marisa cornuarietis*; Mph: *Modiolus philippinarum*; Oct: *Octopus bimaculoides*; Pau: *Phoronis australis*; Pca: *Pomacea canaliculata*; Pfu: *Pinctada fucata*; Mye: *Mizuhopecten yessoensis*; Rau: *Radix auricularia*. The colour bar represents the gene family numbers. Red: high gene family numbers; blue: low gene family numbers. Source data are provided in a Source Data file. 22

Supplementary Figure 11 A heat map of *Gigantopelta aegis* (*n* = 4) showing the gene expression level of genes in the Lysosome KEGG pathway. The colour represents the gene expression level (normalized CPM value). Red: high expression level. Light: low expression level. FI: internal tissue of foot; Ct: ctenidium; M: mantle; OsG: oesophageal gland. Source data are provided in a Source Data file. 23
Supplementary Figure 12 A heat map of *Chrysomallon squamiferum* (*n* = 3) showing the gene expression level of genes in the Lysosome KEGG pathway. The colour represents the gene expression level (normalized CPM value). Red: high expression level. Light: low expression level. F: internal tissue of foot; Ct: ctenidium; M: mantle; OsG: oesophageal gland. Source data are provided in a Source Data file. 24
Supplementary Figure 13 A heat map showed the gene expression level of genes for transporting oxygen and nitrate in *Gigantopelta aegis* (*n* = 4). The colour represents the gene expression level (normalized TPM value). Red: high expression level. Light: low expression level. F: foot; Ct: ctenidium; M: mantle; OsG: oesophageal gland. Source data are provided in a Source Data file. 25
Supplementary Figure 14 Genes with top 50 highest gene expression in the SOB and the MOB of *Gigantopelta aegis* (*n* = 3). Oesophageal glands from three individuals: Ga01, Ga03, and Ga04; TPM: transcripts per million. The colour represents the gene expression level (normalized TPM value). Red: high expression level. Light: low expression level. SOB: sulphur-oxidising symbionts; MOB: methane-oxidising symbionts. Source data are provided in a Source Data file. 26
Supplementary Figure 15 Two phylogenetic trees of *Gigantopelta aegis* symbionts and their free-living relatives. **a**. A phylogenetic tree of *Gigantopelta aegis* sulfur-oxidising endosymbiont (SOB) and its free-living relatives in Gammaproteobacteria. This tree includes one another symbiont *Alviniconcha kojimai*/*strummeri* Gamma-1 SOB and *G. aegis* MOB serves as outgroup. A total of 129 single-copy orthologs were used to construct the tree. **b.** A phylogenetic tree of *Gigantopelta aegis* methane-oxidising endosymbiont (MOB) and its free-living relatives in Gammaproteobacteria (including the symbionts of *Iophon methanophila* and *Hymedesmia (Stylopus) methanophila* sponge, *Bathymodiolus* mussel as well as *Ifremeria* snail; *G. aegis* SOB serves as outgroup). A total of 251 single-copy orthologs were used to construct the tree. 27
Supplementary Figure 16 Biosynthesis pathway of pantothenate (vitamin B_5 in red) and coenzyme A (in red) of *Gigantopelta aegis* holobiont. The *panE* (in red) gene can only be found in the methane-oxidising endosymbiont. The block colour shows the absence (green) and presence (orange) of the gene in the genome. Left: host; middle: sulphur-oxidising endosymbiont, right: methane-oxidising endosymbiont. The description of the gene names are provided in the Supplementary Note 8 Abbreviation List. 28
Supplementary Figure 17 The length distribution of PacBio raw sequencing subreads of *Gigantopelta aegis*. 29
Supplementary Figure 18 The Hi-C contact map of 15 pseudo-chromosomes (chr) of *Gigantopelta aegis*. 30
Supplementary Figure 19 The 17-mer distribution histogram of *Gigantopelta aegis* genome. Source data are provided in a Source Data file. 31

Supplementary Figure 20 The circos plot of the mitochondrial genome of *Gigantopelta aegis*. Inner ring: GC content; outer ring: genes order. CDS: coding DNA sequence.......................32
Supplementary Figure 21 The Clusters of Orthologous Groups (COG) annotation of the sulphur-oxidising bacteria and methane-oxidising bacteria of *Gigantopelta aegis*. One-letter abbreviation for the functional category was determined by the COG database. SOB: blue columns; MOB: orange columns. Source data are provided in a Source Data file. 33
Supplementary Figure 22 A distribution plot shows the Gene Ontology (GO) items of the *Gigantopelta aegis* host (red), the sulphur-oxidising symbionts (SOB, blue), and the methane-oxidising symbionts (MOB, grey). 34
Supplementary Figure 23 Two venn diagrams show the shared gene family numbers between the genes predicted by Prodigal and Prokka of *Gigantopelta aegis* **a.** sulphur-oxidising symbionts SOB and **b**. methane-oxidising symbionts MOB, respectively. Purple: share gene family numbers between genes predicted by Prodigal (blue) and Prokka (pink). Source data are provided in a Source Data file. 35
Supplementary Figure 24 A phylogenetic tree of Neomphalida including *Gigantopelta aegis*, *Dracogyra subfuscus* and *Chrysomallon squamiferum*, as well as other lophotrochozoan references. The references are listed as follows: *Aplysia californica* (GenBank No. GCA_000002075), *Bathymodiolus platifrons*, *Modiolus philippinarum*, *Azumapecten farreri*, *Crassotrea gigas*, *Euprymna scolopes*, *Lanistes nyassanus*, *Marisa cornuarietis*, *Pomacea canaliculata*, *Lingula anatina*, *Lottia gigantea*, *Octopus bimaculoides*, *Phoronis australis*, *Pinctada fucata*, *Mizuhopecten yessoensis*, *Radix auricularia*, and *Haliotis rufescens*. Taxa colour: Cephalopoda: orange; Bivalvia: purple; Gastropoda: red; Brachiopoda: blue; Phoronida: grey; Mollusca: green; and Neomphalida: black. 36
Supplementary Figure 25 The synteny blocks contain genes highly expressed in the oesophageal gland (OsG) of both *Gigantopelta aegis* (n = 4) and *Chrysomallon squamiferum* (n = 3). The colour represents the gene expression level (normalized CPM value). Green: high expression level. Yellow: low expression level. VDG3 (red) is highly expressed in the OsG of *G. aegis* but in the foot of *C. squamiferum*. The description of the protein names are provided in the Supplementary Note 8 Abbreviation List. Source data are provided in a Source Data file. 37
Supplementary Figure 26 The 19-mer distribution histogram of *Dracogyra subfuscus* genome. Source data are provided in a Source Data file. 38

Supplementary Tables

Supplementary Table 1 Summary of the assembly statistics and functional annotation of *Gigantopelta aegis* genome. NR: non-redundant RefSeq protein database; GO: gene ontology database; KEGG: Kyoto encyclopedia of genes and genomes database; KOG: EuKaryotic Orthologous Groups...39
Supplementary Table 2 Classification and composition of repeats content in the genome of *Gigantopelta aegis* gastropod. 40
Supplementary Table 3 Genome assembly results and functional annotation results of the sulphur-oxidising bacteria (SOB) and the methane-oxidising bacteria (MOB) housed in the oesophageal gland of *Gigantopelta aegis*. NR: non-redundant RefSeq protein database; GO:

gene ontology database; KEGG: Kyoto encyclopedia of genes and genomes database; COG: clusters of orthologous groups. 41
Supplementary Table 4 The function of immunity-related gene family that are expanded in the *Gigantopelta aegis* genome. Two-tailed Fisher's exact test and false discovery rate adjustments was applied in the statistics analysis. 42
Supplementary Table 5 Methane oxidation pathways of *Gigantopelta aegis* MOB and its free-living relatives. EMP: Embden–Meyerhof–Parnas; EDD: Entner–Doudoroff; H4MPT: 5,6,7,8-Tetrahydromethanopterin. 43
Supplementary Table 6 Hydrogen oxidation capability of the Gammaproteobacterial symbionts in deep-sea invertebrate taxa. SOB: sulphur-oxidising bacteria; MOB: methane-oxidising bacteria. 44
Supplementary Table 7 Nitrate respiration capability of the Gammaproteobacterial symbionts in deep-sea invertebrate taxa. SOB: sulphur-oxidising bacteria; MOB: methane-oxidising bacteria. 45
Supplementary Table 8 The usage information of each individual of *Gigantopelta aegis*. TEM: transmission electron microscopy; FISH: fluorescence *in situ* hybridisation. 46
Supplementary Table 9 A list of probes used in the fluorescence *in situ* hybridisation experiments. 47
Supplementary Table 10 Genome sequencing data of *Gigantopelta aegis* host and *Dracogyra subfuscus*. PE: paired-end, SE: single-end. 48
Supplementary Table 11 Transcriptome sequencing data of four individuals of *Gigantopelta aegis* (Ga01 [male], Ga02 [female], Ga03 [female], Ga04 [female]) with different dissected tissues. The RNA of oesophageal gland was used to construct both eukaryotic library and bacterial library. meta: metatranscriptome sequencing. 49
Supplementary Table 12 Genome assembly of *Gigantopelta aegis* host by different assembling pipelines. 50
Supplementary Table 13 Available genomes of symbionts belonging to Gammaproteobacteria in deep-sea invertebrate taxa. 51
Supplementary Table 14 Characterisation of *Gigantopelta aegis* mitogenome. 52

Supplementary Note 1: Host Genome Assembly

1.1 Genome Survey

A 17-mer frequency distribution was obtained using the assemble mode of Platanus version 1.2.4[1] in order to assess the genome characteristics, including genome size, heterozygosity and repetitive content.

According to 17-mer histogram of *G. aegis* (Supplementary Figure 19), the genome size was assessed to be 1.21 Gb, the heterozygosity was around 0.5%, and the repeats composition was approximately 50%.

1.2 Genome Assembly Pipelines

Both PacBio-only assembly (Canu version 1.7.1[2] correction [genomeSize = 1.27 Gb, corMhapSensitivity = normal, corMinCoverage = 0, corMaxEvidenceErate = 0.15, correctedErrorRate = 0.065, minReadLength = 8000] + SMARTdenovo [-c 1; https://github.com/ruanjue/smartdenovo]; Canu version 1.7.1[2] correction + wtdbg2 version 2.1[3] [-e 2 --tidy-reads 5000 -S 1 -k 15 -p 0 --rescue-low-cov-edges --aln-noskip]; SMARTdenovo [-c 1]; Minimap2 version 2.12 [-x ava-pb] + miniasm version 0.3-r179[4,5]; Flye version 2.3.3[6]) and hybrid assembler MaSuRCA version 3.2.6[7] pipelines were applied to the genome assembly. In hybrid assembly pipeline, the clean Illumina reads and the Canu corrected subreads were both used. And both the mean and standard deviation of the Illumina library insert size were estimated by Platanus version 1.2.4 assembling[1].

A total of around 121 Gb PacBio raw subreads with an N50 of 10k nt (Supplementary Table 10) and around 171 Gb Illumina raw reads with a length of 150 bp were obtained. Around 52 Gb PacBio corrected subreads with an N50 of 11k nt (Supplementary Table 10) generated an initial assembly result of 9,842 contigs with an N50 of 467 Kb after initial assembly followed with contigs polishing (Supplementary Table 12). MaxBin version 2.2.5[8] was further used to remove 517 contigs of bacterial contamination. Then, 9,325 remaining contigs were manually corrected into 9,479 contigs according to Hi-C maps generated by 3D *de novo* assembly (3D-DNA) pipeline[9] via Juicebox version 1.11.08[10]. A total of 1,607,962,673 pair of Hi-C raw sequencing reads of 482 Gb data generated 56,164,489 pair of valid reads for genome scaffolding of these corrected contigs (Supplementary Table 10). The Hi-C sequencing technology depends on the chromatin formation, which is highly sensitive to the freshness of the samples. For these deep-sea samples, it is very hard to culture them and keep them alive during the deep-sea cruise. Moreover, the low proportion of the valid Hi-C reads of the total reads sequenced is common in Mollusca, mainly due to the high content of mucopolysaccharide in the tissue[11]. However, we tried our best to wash, fix and store the samples. When the gastropods arrived the board, tissue from the foot was immediately dissected, cut up, washed in phosphate-buffered saline buffer for many times, and stored at –80 °C freezer. The samples were immediately transported to Hong Kong with sufficient amount of dry ice. The fixed samples were never thawed until Hi-C library preparation. We also noticed the similar ratio in our former Scaly-foot Snail genome Hi-C library preparation[11], and therefore, we aimed to sequence over 300X of the estimated genome to get enough valid reads.

A total of 5,231 scaffolds with a size of 1.15 Gb and a scaffolds N50 of 81.6 Mb included 15 pseudo-chromosomes (Supplementary Table 1). Among the contigs, 5,216 of them were not anchored into the chromosome groups due to the lack of Hi-C linkage among them or their high repetitiveness (Supplementary Table 1). In the assembled genome, there are 4,248 gaps with a fixed length of 200 bp. These gaps were generated when the assembled contigs were further scaffolded using Hi-C data, and these gaps represent gaps of the unknown size. In the host genome, 50.8 % of the genome were repeats and 74% of these repeats are unclassified (Supplementary Figure 1, Supplementary Table 2). The Hi-C contact maps of the 15 pseudo-chromosomes were showed in the Supplementary Figure 18. BUSCO (Benchmarking Universal Single-Copy Orthologs) assessment showed the assembled genome had around 94% completeness.

Supplementary Figure 5 shows the genome binning results using genome sequencing data generated from the oesophageal gland, the tissue harhouring the two symbionts. The additional bacterial contamination (except the two symbiont genomic contigs) represents the initial genome binning assembly results of the metagenome. The Illumina sequencing data, PacBio sequencing data and Hi-C sequencing data used for *G. aegis* host genome assembly were all generated from foot tissue that does not have symbionts (Supplementary Table 8). To ensure there is no bacterial contamination, CheckM version 1.0.13[12] was used to check the potential contamination of the assembly results. The initial assembly results without using MaxBin version 2.2.5[8] showed that it included one bacteria genome that has 98.28% completeness and 63.56% potential contamination. After using MaxBin version 2.2.5[8], 517 contigs were removed. The BUSCO completeness of the host genome was not reduced after removing the bacterial contamination. The CheckM results of this clean assembly showed 0% contamination of bacteria. Therefore, bacteria sequences were barely found in the scaffolds of *G. aegis*, suggesting a lack of horizontal gene transfer between the host and the symbionts.

1.3 Mitochondria Genome Assembly and Characteristics

The mitochondrial genome was assembled by MEGAHIT version 1.1.1[13] using the clean Illumina reads. MITOS Web Server[14] was used to annotate the protein-coding genes (PCGs), transfer RNA (tRNA) genes, and ribosomal RNA (rRNA) genes of the mitochondrial genome.

A near complete mitochondrial genome of *G. aegis* was assembled into one contig with a length of 16,097 nt (Supplementary Figure 20). It possesses 37 genes, including 13 protein coding genes, 22 tRNA genes, and 2 rRNA genes (Supplementary Table 14). Twenty genes are in the plus strand, and other 17 ones are in the negative strand. Among the protein coding genes, *nad4l* and *nad4* have a 3 bp overlap.

Supplementary Note 2: Host Genome Annotation

2.1 Repeats Annotation

The species-specific repeats library of the *G. aegis* was *de novo* identified and classified by RepeatModeler version 1.0.11 (http://www.repeatmasker.org/RepeatModeler/) pipeline implemented with RepeatScout version 1.0.5[15], RECON version 1.08[16], TRF version 4.09[17], and NSEG[18]. The repeats of the genome were searched against the species-specific library, RepBase library (RepeatMasker Edition released on 2018, October 26th)[19] as well as Dfam library version 2.0[20] by NCBI RMBlast version 2.6.0, and the hitted regions were further soft-masked by RepeatMasker version 4.0.8 (http://www.repeatmasker.org/RMDownload.html) with a parameter -xsmall.

2.2 Genes Prediction of the Host

To obtain EST evidence, Trinity version 2.8.3[21] was used to *de novo* assemble and genome-guided assemble the transcripts using the transcriptome sequencing data of all available organs. The *de novo* assembled and genome-guided assembled transcripts were integrated by PASA pipeline version 2.2.0[22] with standalone BLAT version 36x4[23]. The redundancy was removed from the integrated transcripts by using CD-HIT-EST version 4.6.8[24] with -c 0.95. The non-redundant transcripts served as EST evidence in the gene prediction. To train the model of *ab initio* gene prediction, first round of MAKER was performed with only EST evidence by using est2genome = 1. The predicted genes of this round of MAKER were only used for training *ab initio* gene prediction model. Only the genes with an annotation edit distance (AED) score equal to zero, with the distances of the neighbouring genes larger than 3 kb, and with more than 3 exons, were used to training Augustus version 3.2.3[25]. After that, MAKER version 2.31.10[26] was performed for a second round to predict genes of the genome with all evidences.

A total of 21,438 genes were predicted from the host genome. Among them, 19,286 (90%) genes had significant hits in the NCBI NR database, 7,478 (34.9%) in the KEGG database, 13,543 (63.2%) in the KOG database, and 19,135 (89.3%) genes had hits in the functional domains of Pfam database.

2.3 Genome Assembly and Gene Prediction of Symbionts

Besides the SOB and the MOB, other bacteria species with low abundance were also found in the sequencing data. It was either contamination from sea water or symbiont that may barely contribute to the symbiosis due to such low abundance. For genome assembly of symbionts, we obtained a total of 4,528,840 Nanopore subreads of 5.77 Gb and 493,104,042 clean Illumina short reads. In the SOB genome, 5,105 genes (92.5%) had hits in the NR database, 3,584 (65.0%) in the GO database, 1,813 (32.9%) in the KEGG database, and 4,405 (79.8%) in the COG database (Supplementary Table 3). In the MOB genome, 3,019 (97.3%) genes were annotated in the NR database, 1,969 (63.5%) in the GO database; 1,525 (49.2%) in the KEGG database, and 2,746 (88.5%) were assigned to the COG categories (Supplementary Table 3). The COG annotation and GO annotation of the SOB and the MOB were classified into different functional categories (Supplementary Figure 21, Supplementary Figure 22).

The genes prediction results mentioned above were generated from Prodigal version 2.6.3[27]. Prokka version 1.14.6[28] was also used to further check the accuracy of genes prediction of both SOB and MOB. The genes predicted by these two software are highly consistent, indicating by the Venn diagrams of their shared gene families (Supplementary Figure 23). The functional annotation results of the genes predicted from Prokka are provided in Supplementary Data 3 (SOB) and Supplementary Data 4 (MOB).

Supplementary Note 3: Molecular Clock Analysis

Dracogyra subfuscus, *Chrysomallon squamiferum*[11], *Aplysia californica* (GenBank No. GCA_000002075), *Bathymodiolus platifrons*[29], *Modiolus philippinarum*[29], *Azumapecten farreri*[30], *Crassotrea gigas*[31], *Euprymna scolopes*[32], *Lanistes nyassanus*[33], *Marisa cornuarietis*[33], *Pomacea canaliculata*[33], *Lingula anatina*[34], *Lottia gigantea*[35], *Octopus bimaculoides*[36], *Phoronis australis*[37], *Pinctada fucata*[38], *Mizuhopecten yessoensis*[39], *Radix auricularia*[40], and *Haliotis rufescens*[41] were used as genomics references in the phylogeny analysis and molecular clock analysis.

The following 7 fossil records and geographic events were used to calibrate the phylogenetic tree (Supplementary Figure 24): minimum = 465.0 Ma for the first appearance of Pteriomorpha[42]; and minimum = 168.6 Ma and soft maximum = 473.4 Ma for *A. californica* and *R. auricularia*[43]; a hard max time-point of 150 Ma for *L. nyassanus and P. canaliculata*, which correspond to the split of South America and Africa[44]; hard minimum bound = 390 Ma for Caenogastropoda and Heterobranchia[45]; minimum = 470.2 Ma and soft maximum = 531.5 Ma for *A. californica* and *L. gigantean*[43], and minimum = 532 Ma and soft maximum = 549 Ma for the first appearance of molluscs[46]; and minimum = 550.25 Ma and soft maximum = 636.1Ma for the first appearance of Lophotrochozoan[46].

Supplementary Note 4: Gene Family Expansion

Several gene families that are involved in immune recognition were expanded (Supplementary Table 4). Carcinoembryonic antigen-related cell adhesion molecule 5 (CEACAM5) and multiple epidermal growth factor-like domains protein 10 (MEGF) were reported as microbial recognition receptor in oysters[47]. The expansion of these gene families help improve the diversity of microbial recognition patterns, which may help *G. aegis* to recognise its two physiologically distinct types of endosymbionts.

Transposase genes are particularly enriched in bacterial lineages that recently transitioned to a host-associated lifestyle[48,49] and are correspondingly reduced in the ancient, host-restricted bacterial lineages[50]. Transposase enrichment is thus a sign of a recent host-associated lifestyle for the symbionts of *G. aegis*, especially compared to symbionts of *C. squamiferum*. This is corroborated by the fact that the genome size of SOBs in *G. aegis* (4.91 Mb) is much larger than that of the SOBs in *C. squamiferum* (2.59 Mb[51]). Cryptometamorphosis in *Gigantopelta*

may be interpreted as an intermediate step before acquiring full reliance on endosymbiosis immediately after settlement, as is the case in *C. squamiferum*. It is possible that *C. squamiferum* also went through a cryptometamorphosis stage sometime during the evolution of the holobiont condition. Since its discovery, evidence has appeared that cryptometamorphosis is more widespread and not unique to *Gigantopelta*. For example, the giant chemosymbiotic shipworm *Kuphus polythalamius* initially settles on wood (like other non-chemosymbiotic shipworms) and only later acquires chemosymbiosis when moving into mud[52]. Such occurrence of cryptometamorphosis in a completely independent chemosymbiotic lineage is suggestive that perhaps a cryptometamorphosis stage is a common 'stepping stone' route towards immediate formation and reliance on symbiosis upon settlement.

Supplementary Note 5: Proteomic Approach

The oesophageal glands from three *Gigantopelta aegis* were dissected, lysed in a buffer (8 M urea, 40 mM HEPES, pH = 8.0), and sonicated by QSonica (Newtown, CT, USA). The mixtures were centrifuged at 15,000 g for 15 min. Methanol-chloroform protein precipitation method[53] was used to purify the supernatant and concentrate the protein yielded. SDS-PAGE (sodium dodecyl sulphate–polyacrylamide gel electrophoresis) gel was used to separate different size of ~30 µg extracted protein, and stained by colloidal coomassie blue. Each gel was cut into 6 slices and dehydrated in an ACN buffer (100 mM NH_4HCO_3, 50 mM NH_4HCO_3 and 50% ACN, and 100% ACN). The peptide for LC-MS/MS was obtained through protein reduction (10 mM DTT for 45 min at 56 °C; alkylated by 55 mM iodoacetamide for 20 min in dark), protein digestion (sequencing grade Trypsin for 14 hours at 37 °C), peptide extraction (5% formic acid in 50% ACN and 100% ACN sequentially) and further dry (speed-vacuum and desalted with a C18 Sep-Pak column).

Dionex UltiMate 3000 RSLCnano coupled with an Orbitrap Fusion Lumos Mass Spectrometer (Thermo Fisher) was utilised to analyse each protein fraction with the settings: flow rate at 300 nL/min, positive ion mode, 400−1500 m/z scan range, 60,000 MS resolution, 1.6 m/z isolation window, 40 s dynamic exclusion duration, 4.0e5 AGC target, 30% HCD collision energy, and 110 m/z first mass.

The search database contains the protein sequences predicted from the genome and the corresponding reversed sequences (decoy) of both *Gigantopelta aegis* and its two endosymbionts. Mascot version 2.3.0 was used to identify and quantify the protein via the raw mass spectrometry data with settings: 0.6 Da for fragments, 5 ppm for precursor, fixed modification: carbamidomethyl (cysteine), variable modification: oxidation (methionine), and up to two missed trypsin cleavage. Peptide with an expectation level smaller than 0.05 was filtered with a false discovery rate of 2.5%.

Supplementary Note 6: Synteny

The distribution pattern of non-cross chromosomal synteny was presented in mollusca and in deep-sea fauna, which may also exist in other closely related groups. These synteny contains genes highly expressed in the oesophageal gland of both *G. aegis* and *C. squamiferum* except the VDG3 gene (Supplementary Figure 25) that are involved in regulating the development of the digestive system in Mollusca[54,55].

Supplementary Note 7: *Dracogyra subfuscus* Genome

The whole tissue of *Dracogyra subfuscus* was used for DNA extraction and genome sequencing. A 19-mer frequency distribution generated from the assemble mode of Platanus version 1.2.4[1] was used to assess the genome characteristics. According to 19-mer histogram of *Dracogyra subfuscus* (Supplementary Figure 26), the genome size was assessed to be approximately 1.2 Gb and repeats composition was 56%.

A draft genome of 444,004 contigs with a total length of 1.16 Gb and a N50 of 5,907 nt was assembled by using 1,584,697,209 Illumina clean reads. A total of 5,986 genes was predicted from the draft genome. The sequencing data generated from the whole tissue of *D. subfuscus* and the draft genome were used for genome binning analysis following the method applied to *G. aegis* symbionts to identify potential symbionts. The binning results were compared to that of *G. aegis*, which was relied on the sequencing data of the oesophageal gland. The genes identified from this draft genome was only used in the phylogenetic analysis and molecular clock analysis.

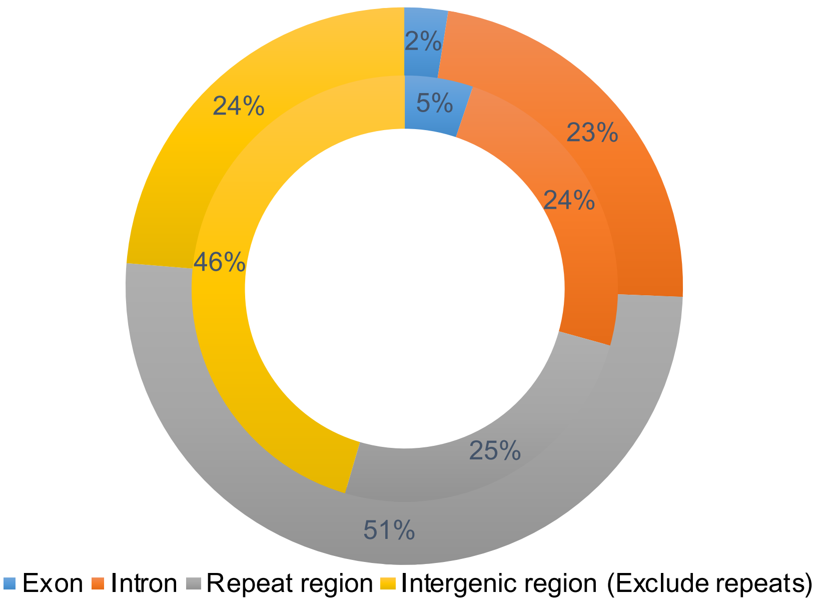

Supplementary Figure 1 Composition of different genomic components of *Gigantopelta aegis* and *Chrysomallon squamiferum*. Outer ring: *G. aegis*; Inner ring: *C. squamiferum*. Exon: blue; intron: orange; repeat region: grey; intergenic region (exclude repeats): yellow. Source data are provided in a Source Data file.

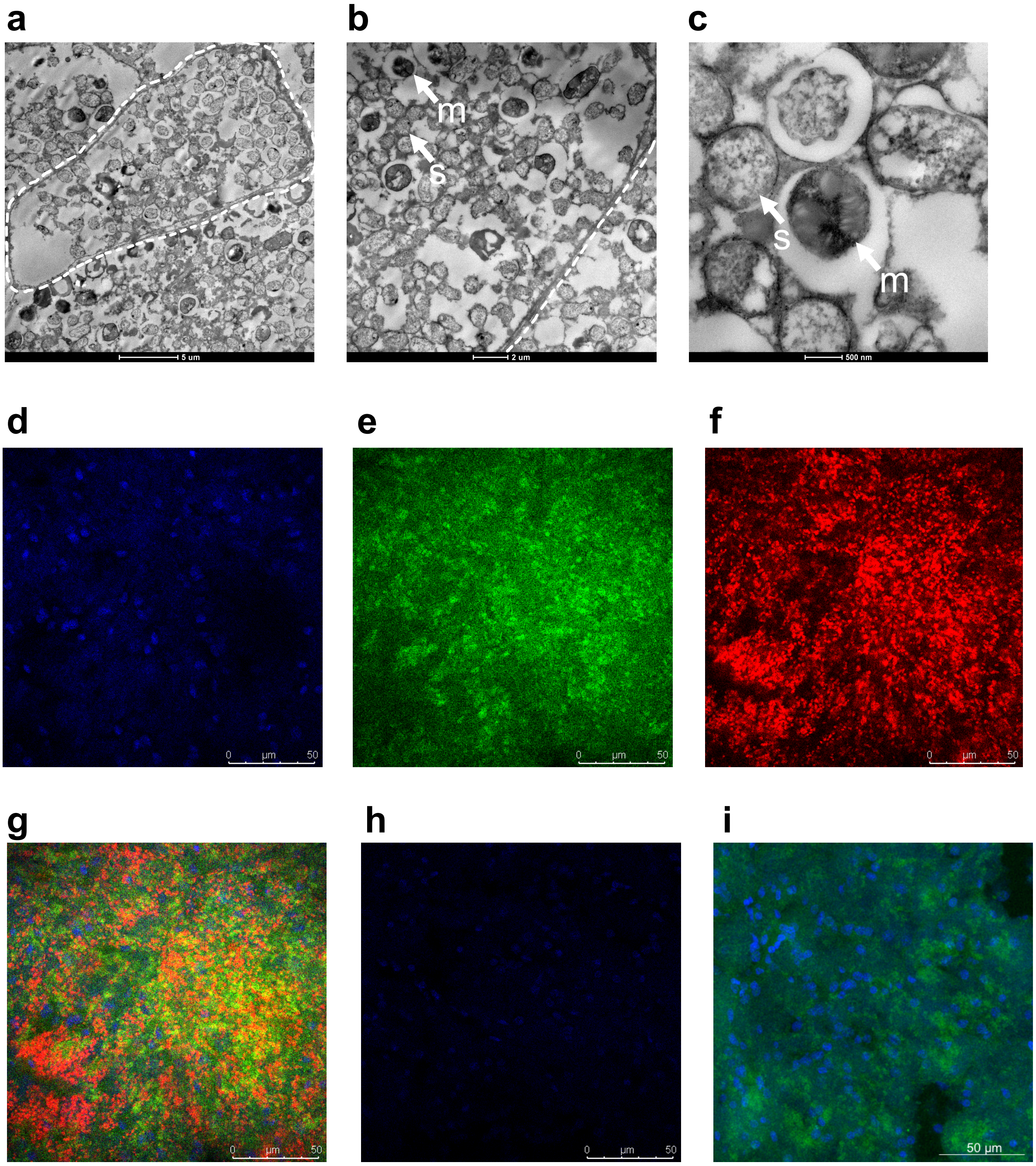

Supplementary Figure 2 Transmission electron microscopy (TEM) and fluorescence *in situ* hybridisation (FISH) images of the oesophageal gland from *Gigantopelta aegis*. The TEM images of a. an entire bacteriocyte cell housing intracellular endosymbiont (scale bar: 5 µm), b. endosymbionts showing two distinct morphological types (scale bar: 2 µm), and c. one endosymbiont showing intracellular stacked membranes (scale bar: 500 nm). white outline: bacteriocyte cell membrane; s: sulphur-oxidising symbiont; m: methane-oxidising symbiont. FISH images of d. host nuclear DNA, e. sulphur-oxidising bacteria (SOB), f. methane-oxidising bacteria (MOB), and g. the merged signal of d., e., and f. on transverse sections of oesophageal gland from *Gigantopelta aegis* (scale bar: 50 µm). FISH experiments were performed with specific 16S rRNA probes for SOB and MOB. FISH image of h. Negative control: DNA (DAPI staining) and NON338 probe[56] The signal of NON338 is bare. i. Positive

control: DNA (DAPI staining) and universal EUB338 probe[57]: green signal. Colour: DNA (DAPI staining): blue; SOB (Cy3): green; MOB (Cy5): red. TEM experiments were applied on 3 individuals with more than 3 thin sections each. FISH experiments were repeated independently for twice and at least 10 sections of the samples were used for each time. These experiments were repeated with similar results.

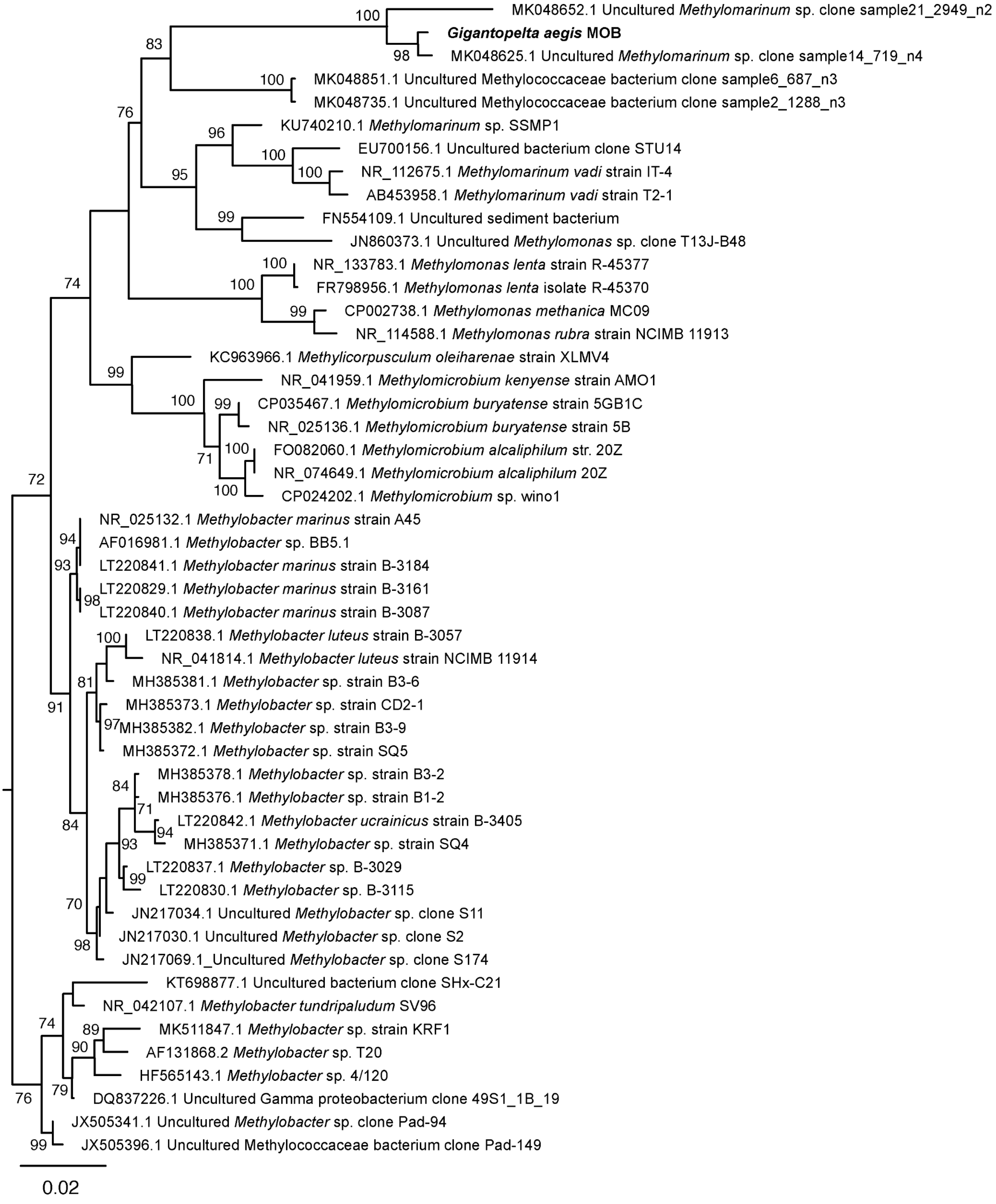

Supplementary Figure 3 A phylogenetic tree of *Gigantopelta aegis* MOB and its closest relatives based on 16S rRNA gene.

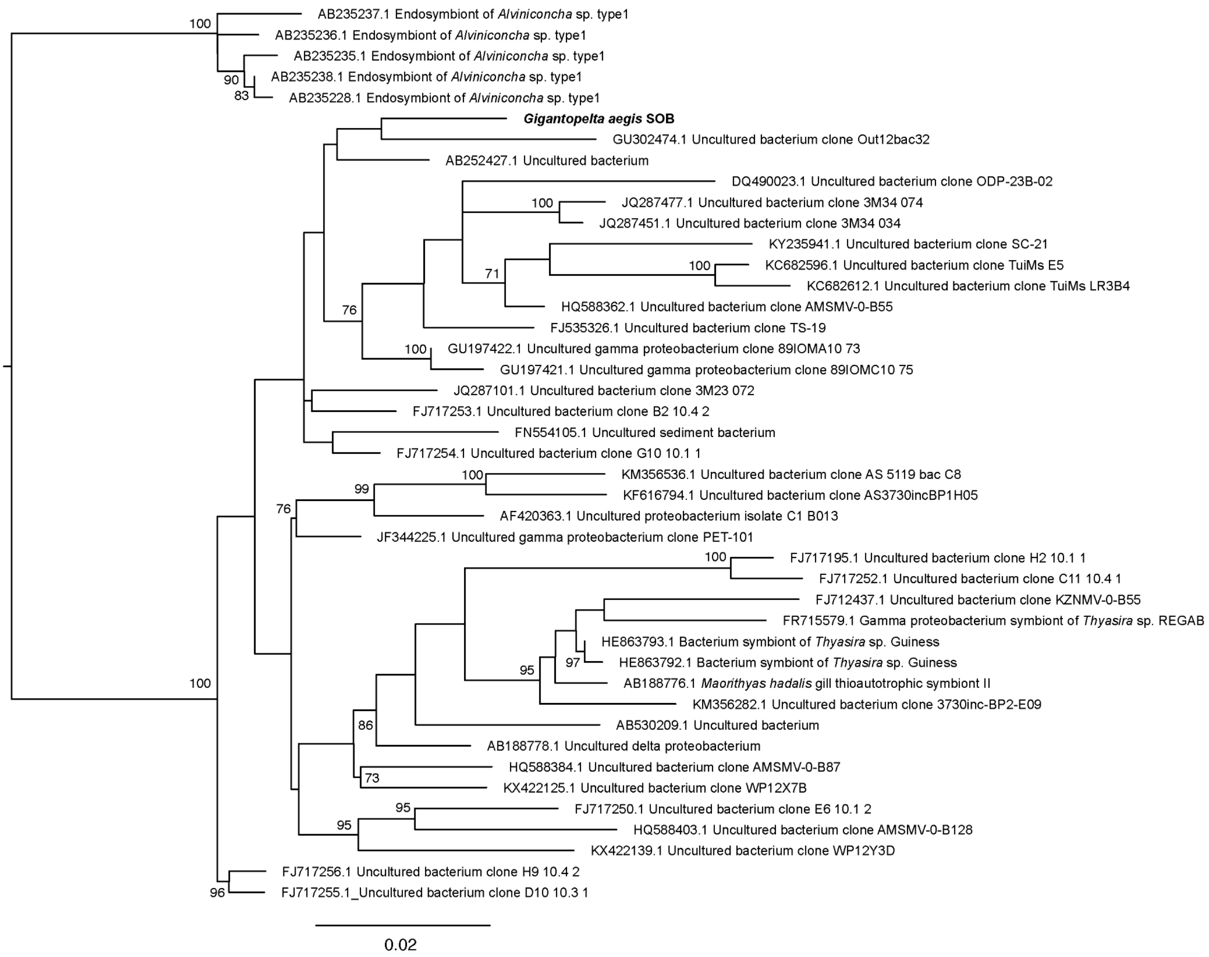

Supplementary Figure 4 A phylogenetic tree of *Gigantopelta aegis* sulphur-oxidising symbiont (SOB) and its closest relatives based on 16S rRNA gene.

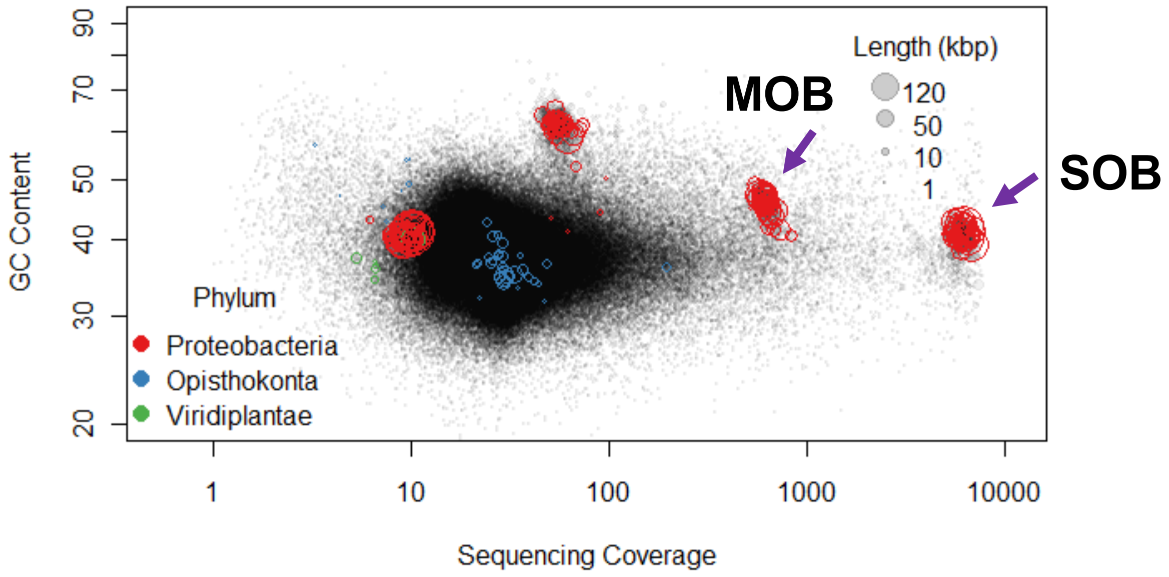

Supplementary Figure 5 Genome binning of the initially assembled contigs from the oesophageal gland of *Gigantopelta aegis*. Two dominant bacteria had significantly higher coverage than the host. Each dot represents a contig. SOB: sulphur-oxidising bacteria; MOB: methane-oxidising bacteria. The colour labelling scheme of phylum: red: proteobacteria; blue: opisthokonta; green: viridiplantae.

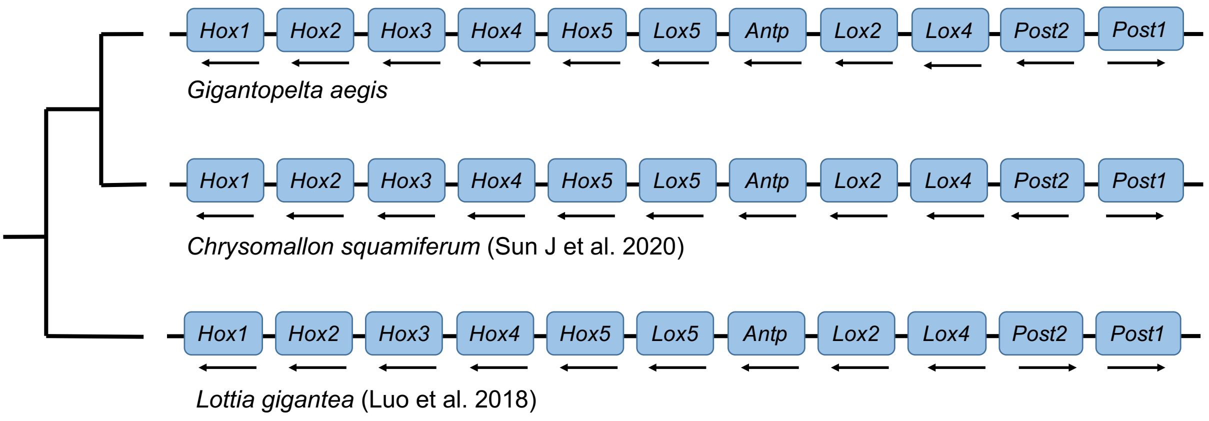

Supplementary Figure 6 The *Hox* clusters shared same gene order between *Gigantopelta aegis* and *Chrysomallon squamiferum*. The description of the protein names are provided in the Supplementary Note 8 Abbreviation List.

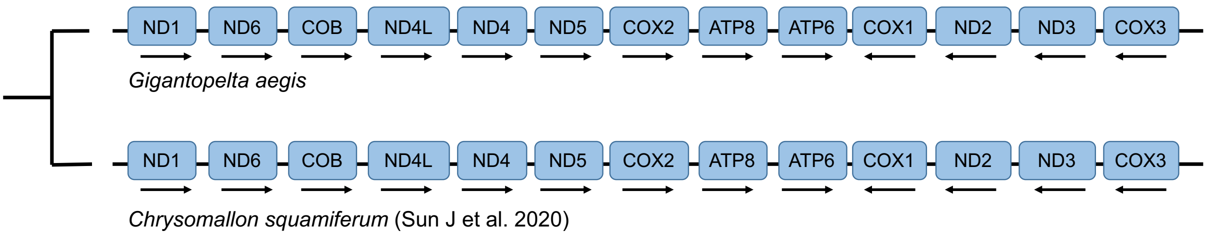

Supplementary Figure 7 The 13 protein-coding genes of mitochondrial genomes shared same gene order between *Gigantopelta aegis* and *Chrysomallon squamiferum*. The description of the gene names are provided in the Supplementary Note 8 Abbreviation List.

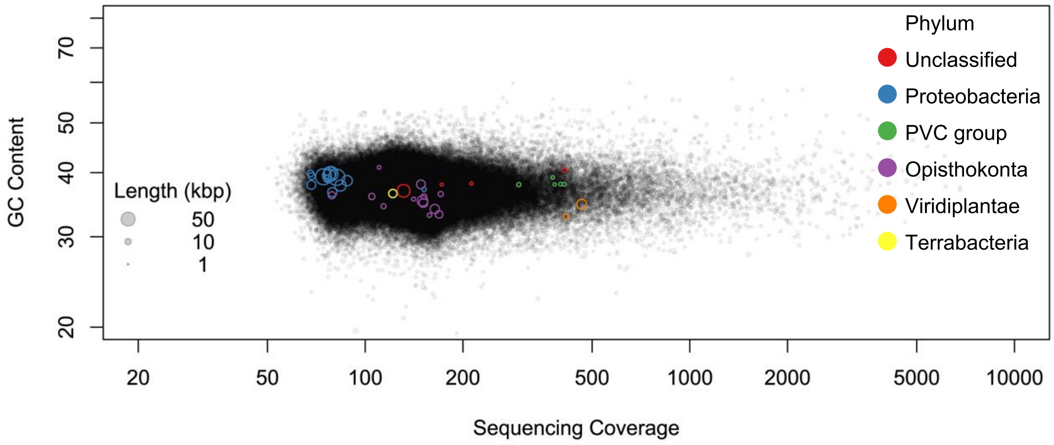

Supplementary Figure 8 Genome binning of the initially assembled contigs of *Dracogyra subfuscus*. Each dot represents a contig. The colour labelling scheme of phylum: red: unclassified organism; blue: proteobacteria; green: bacteria named after planctomycetes, verrucomicrobia, and chlamydiae (PVC) group; purple: opisthokonta; orange: viridiplantae; and yellow: terrabacteria.

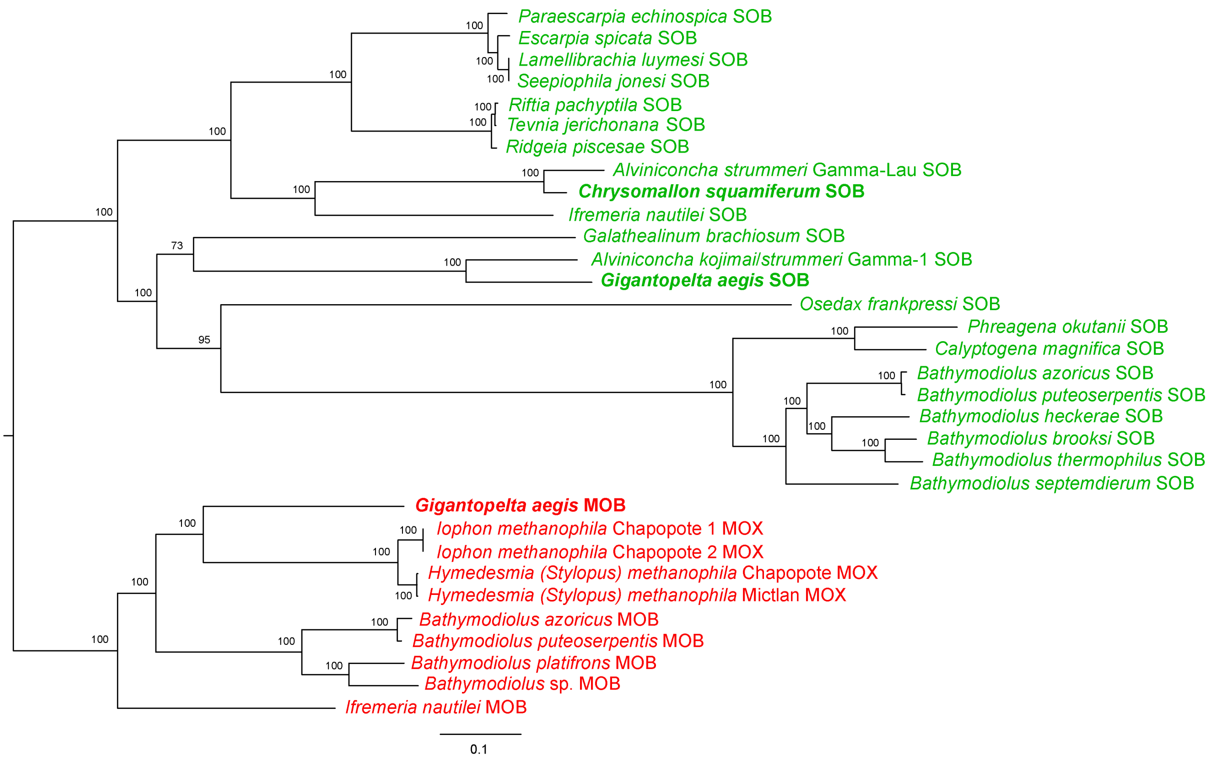

Supplementary Figure 9 Phylogenetic reconstruction of 32 symbionts in Gammaproteobacteria from invertebrate taxa living in deep-sea chemosynthetic environments. A total of 424 single-copy orthologs were used to construct the tree. The phylogenetic tree includes two major lineages of the sulphur-oxidising bacteria (green) and the methane-oxidising bacteria (red). The information of 32 symbionts is provided in Supplementary Table 13.

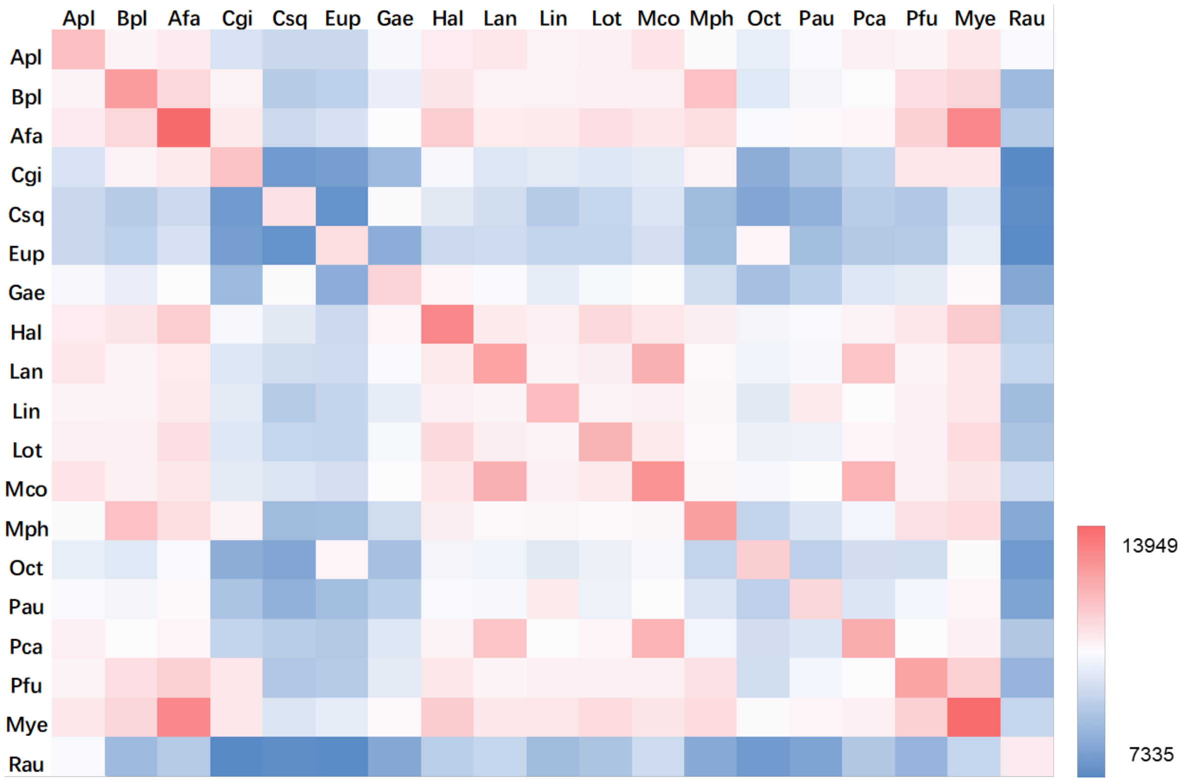

Supplementary Figure 10 A heat map of shared gene family numbers of *Gigantopelta aegis* (Gae) and other references. Apl: *Aplysia californica* [GenBank No. GCA_000002075]; Bpl: *Bathymodiolus platifrons*; Afa: *Azumapecten farreri*; Cgi: *Crassotrea gigas*; Csq: *Chrysomallon squamiferum*; Eup: *Euprymna scolopes*; Hal: *Haliotis rufescens*; Lan: *Lanistes nyassanus*; Lin: *Lingula anatina*; Lot: *Lottia gigantea*; Mco: *Marisa cornuarietis*; Mph: *Modiolus philippinarum*; Oct: *Octopus bimaculoides*; Pau: *Phoronis australis*; Pca: *Pomacea canaliculata*; Pfu: *Pinctada fucata*; Mye: *Mizuhopecten yessoensis*; Rau: *Radix auricularia*. The colour bar represents the gene family numbers. Red: high gene family numbers; blue: low gene family numbers. Source data are provided in a Source Data file.

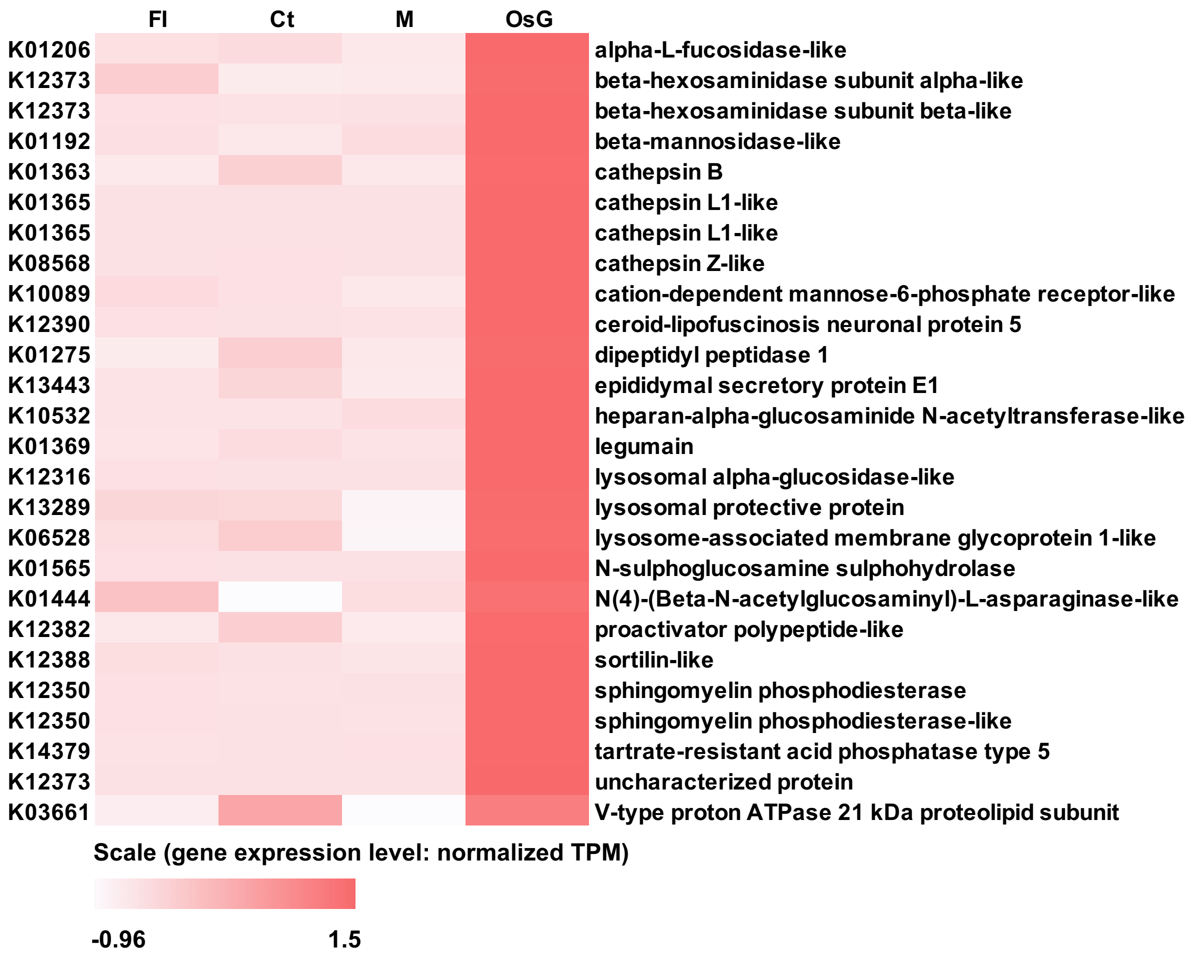

Supplementary Figure 11 A heat map of *Gigantopelta aegis* (n = 4) showing the gene expression level of genes in the Lysosome KEGG pathway. The colour represents the gene expression level (normalized CPM value). Red: high expression level. Light: low expression level. FI: internal tissue of foot; Ct: ctenidium; M: mantle; OsG: oesophageal gland. Source data are provided in a Source Data file.

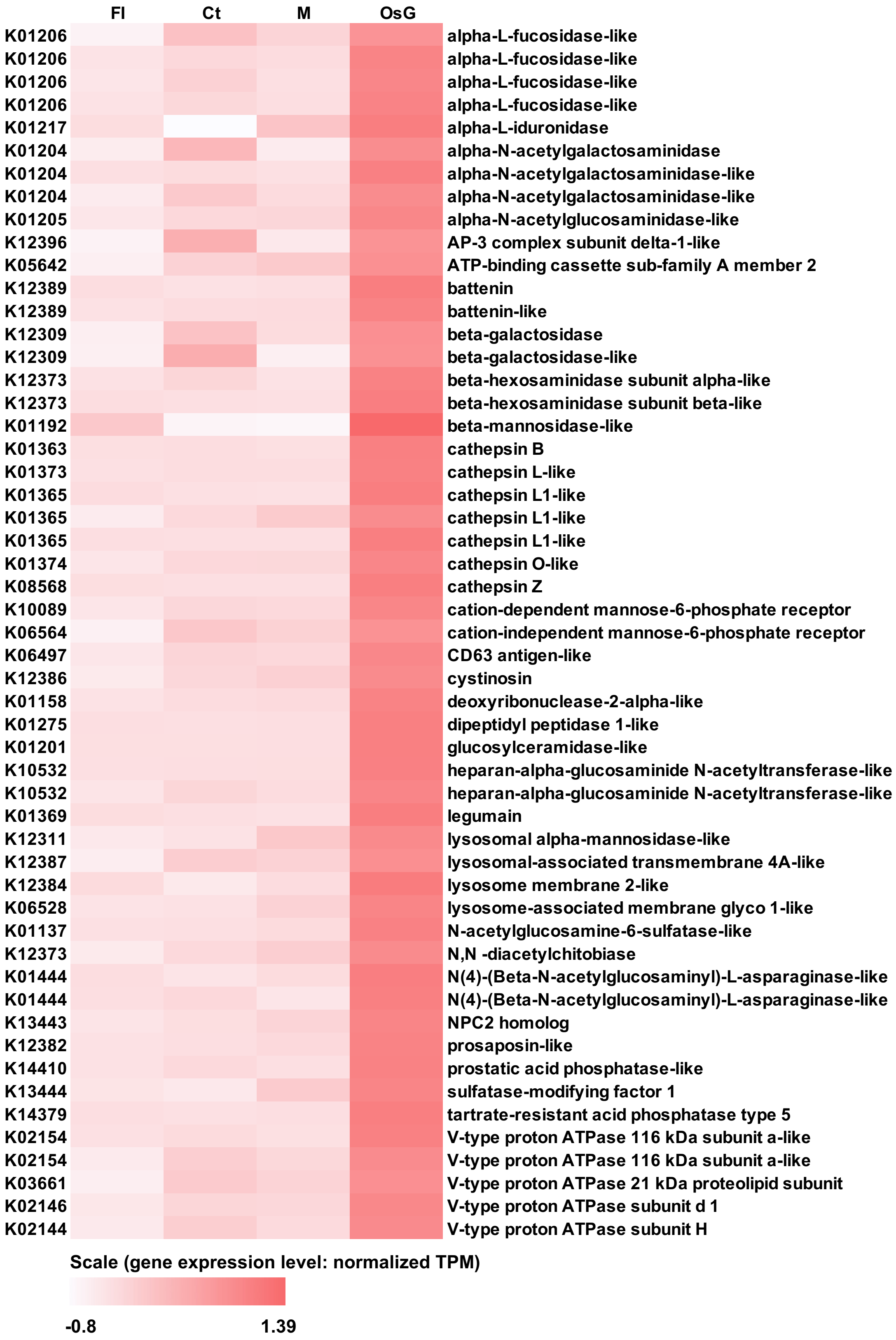

Supplementary Figure 12 A heat map of *Chrysomallon squamiferum* (*n* = 3) showing the gene expression level of genes in the Lysosome KEGG pathway. The colour represents the gene expression level (normalized CPM value). Red: high expression level. Light: low expression level. F: internal tissue of foot; Ct: ctenidium; M: mantle; OsG: oesophageal gland. Source data are provided in a Source Data file.

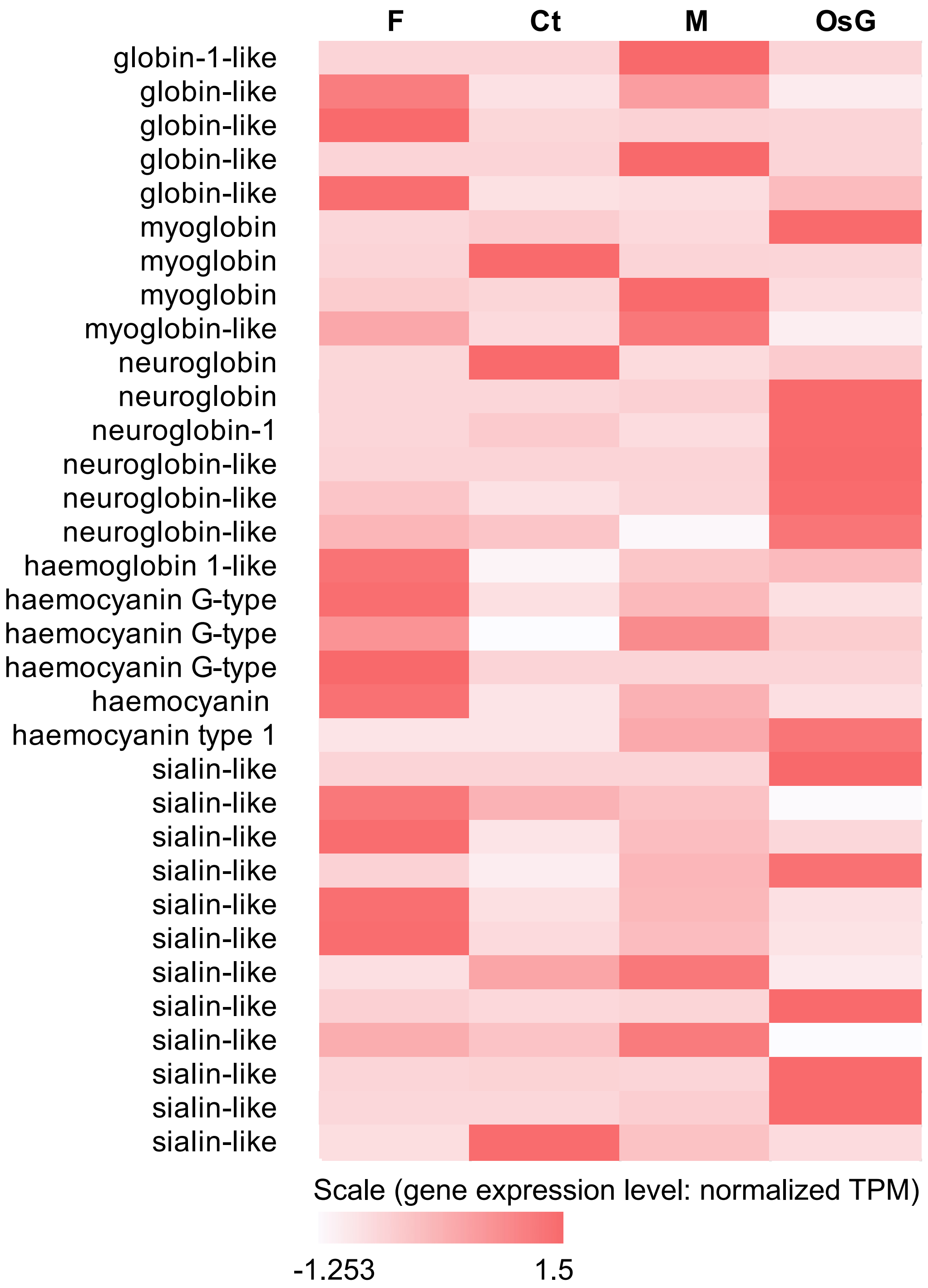

Supplementary Figure 13 A heat map showed the gene expression level of genes for transporting oxygen and nitrate in *Gigantopelta aegis* (n = 4). The colour represents the gene expression level (normalized TPM value). Red: high expression level. Light: low expression level. F: foot; Ct: ctenidium; M: mantle; OsG: oesophageal gland. Source data are provided in a Source Data file.

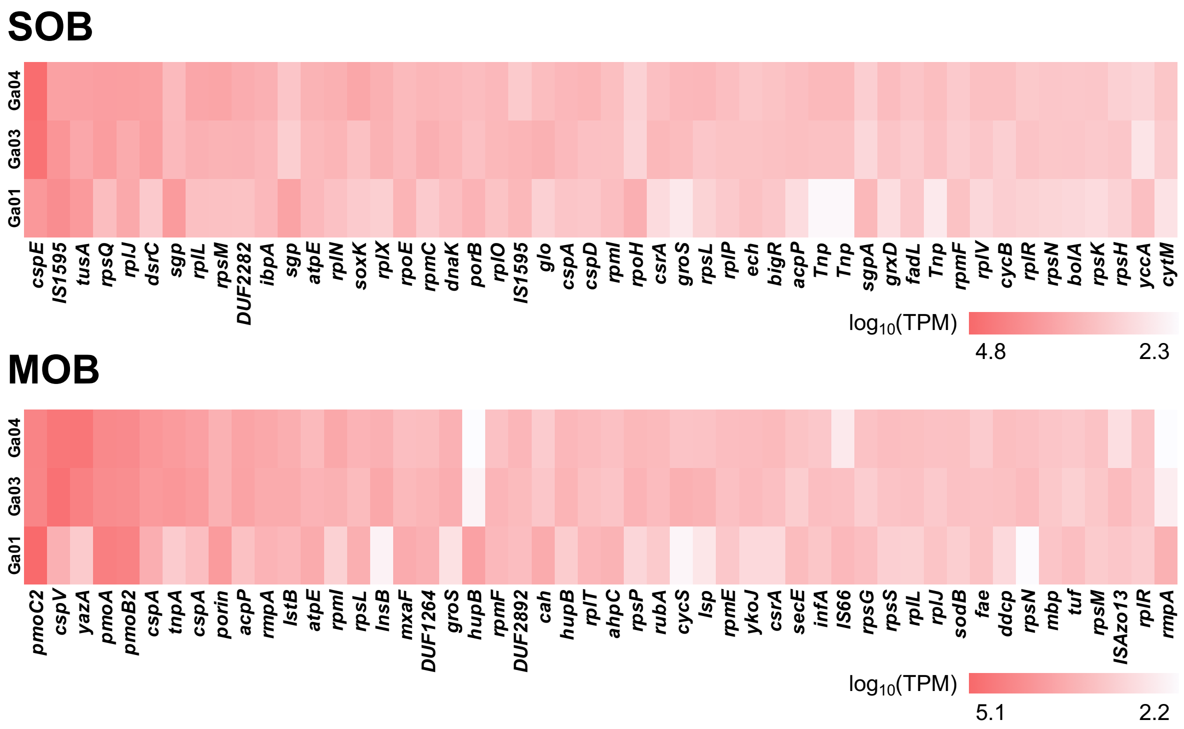

Supplementary Figure 14 Genes with top 50 highest gene expression in the SOB and the MOB of *Gigantopelta aegis* ($n = 3$). Oesophageal glands from three individuals: Ga01, Ga03, and Ga04; TPM: transcripts per million. The colour represents the gene expression level (normalized TPM value). Red: high expression level. Light: low expression level. SOB: sulphur-oxidising symbionts; MOB: methane-oxidising symbionts. Source data are provided in a Source Data file.

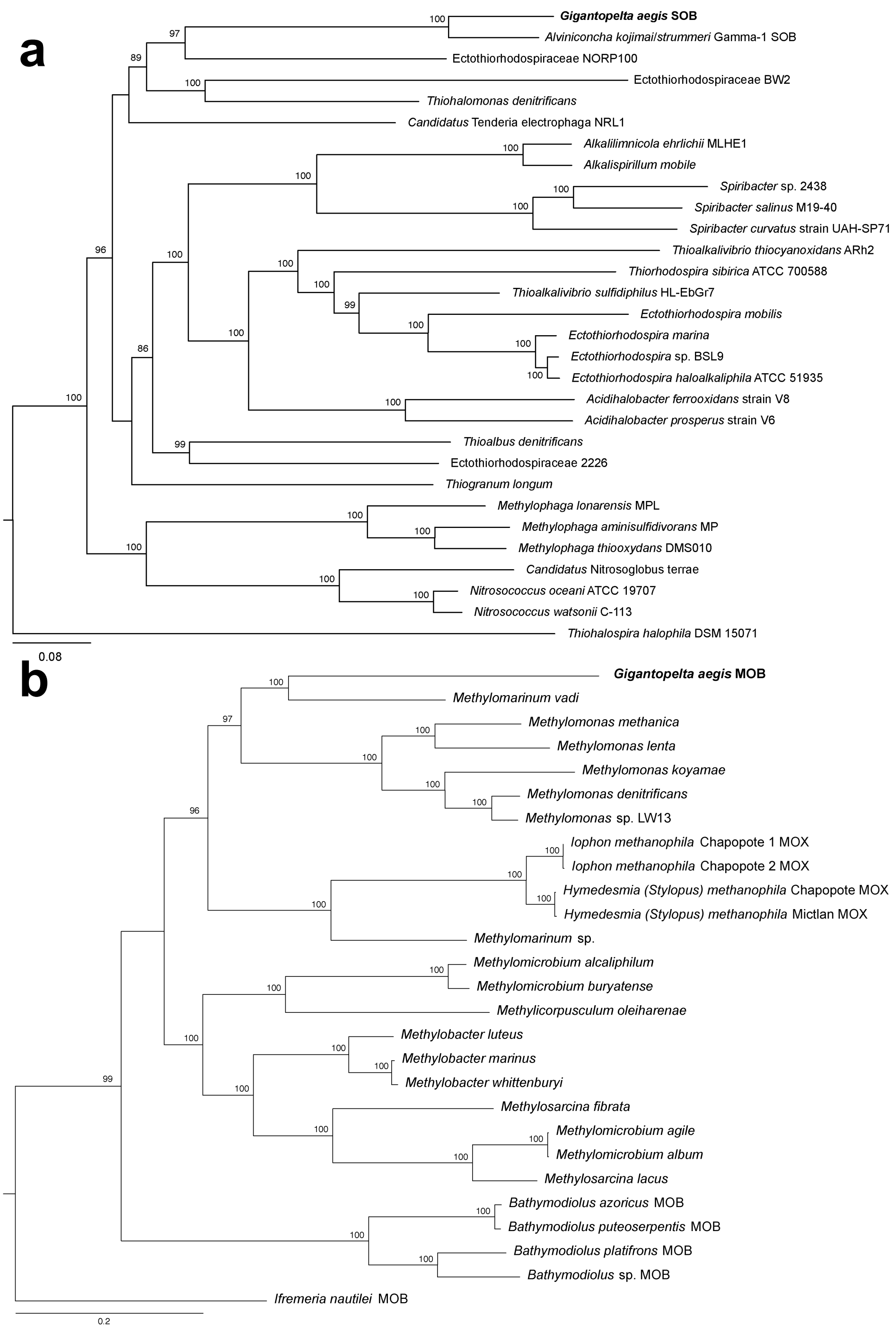

Supplementary Figure 15 Two phylogenetic trees of *Gigantopelta aegis* symbionts and their free-living relatives. **a**. A phylogenetic tree of *Gigantopelta aegis* sulfur-oxidising endosymbiont (SOB) and its free-living relatives in Gammaproteobacteria. This tree includes one another symbiont *Alviniconcha kojimai/strummeri* Gamma-1 SOB and *G. aegis* MOB serves as outgroup. A total of 129 single-copy orthologs were used to construct the tree. **b.** A phylogenetic tree of *Gigantopelta aegis* methane-oxidising endosymbiont (MOB) and its free-living relatives in Gammaproteobacteria (including the symbionts of *Iophon methanophila* and *Hymedesmia (Stylopus) methanophila* sponge, *Bathymodiolus* mussel as well as *Ifremeria* snail; *G. aegis* SOB serves as outgroup). A total of 251 single-copy orthologs were used to construct the tree.

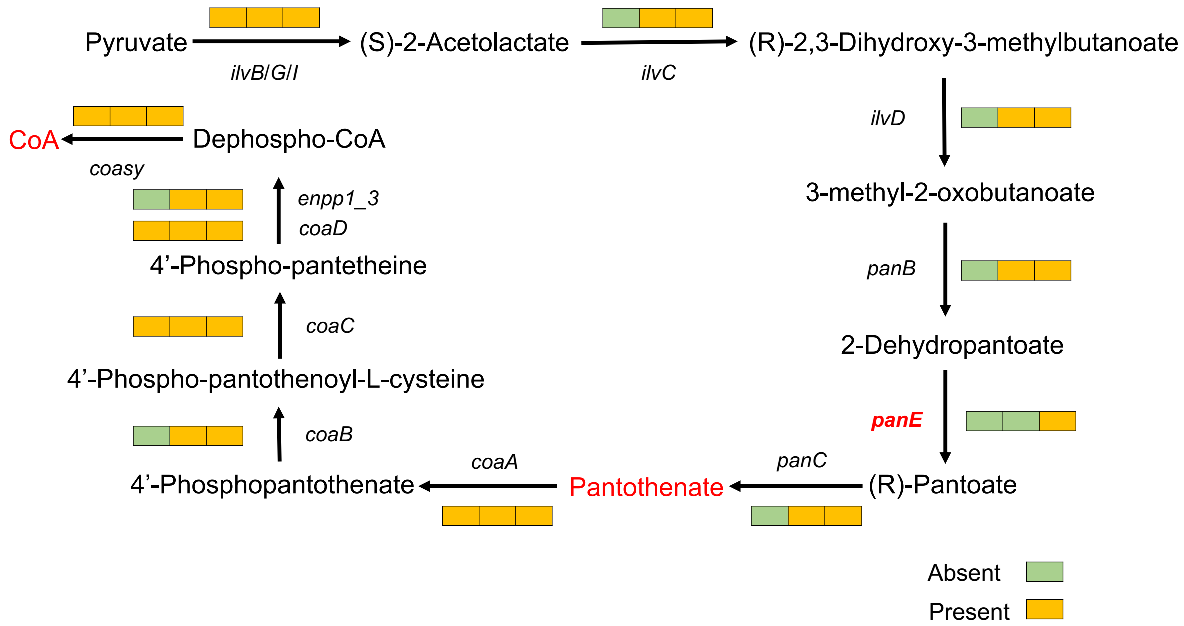

Supplementary Figure 16 Biosynthesis pathway of pantothenate (vitamin B_5 in red) and coenzyme A (in red) of *Gigantopelta aegis* holobiont. The *panE* (in red) gene can only be found in the methane-oxidising endosymbiont. The block colour shows the absence (green) and presence (orange) of the gene in the genome. Left: host; middle: sulphur-oxidising endosymbiont, right: methane-oxidising endosymbiont. The description of the gene names are provided in the Supplementary Note 8 Abbreviation List.

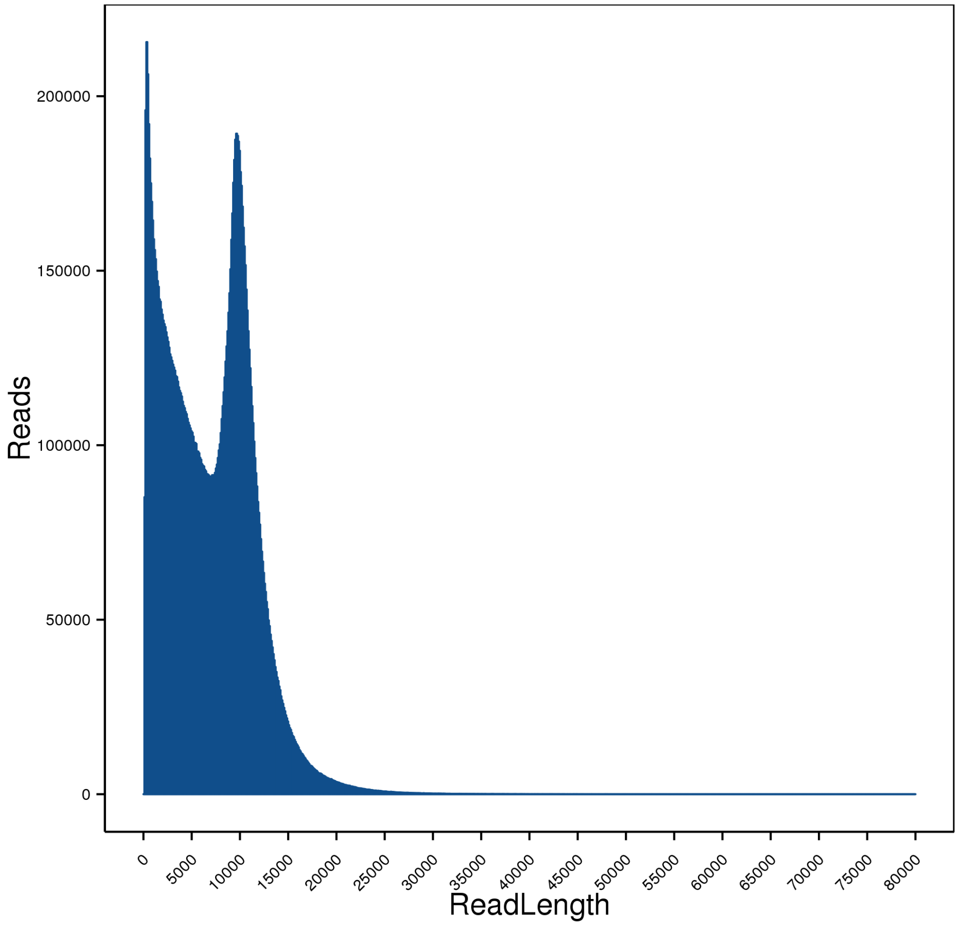

Supplementary Figure 17 The length distribution of PacBio raw sequencing subreads of *Gigantopelta aegis*.

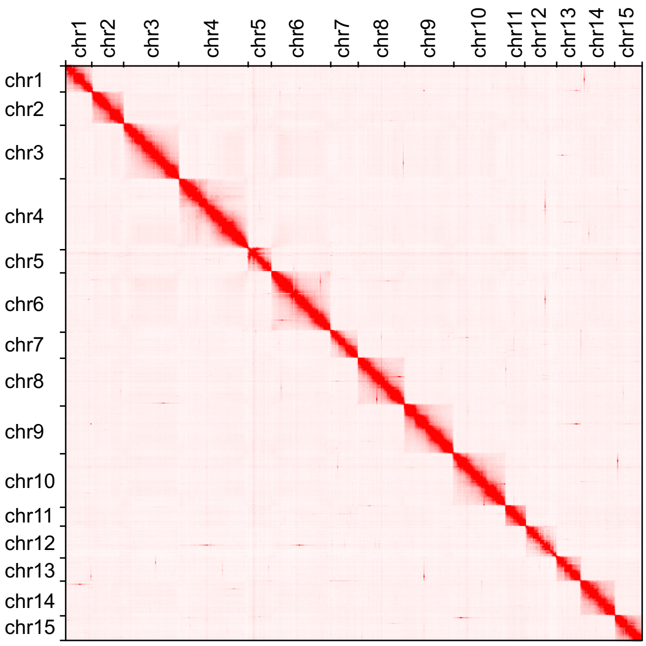

Supplementary Figure 18 The Hi-C contact map of 15 pseudo-chromosomes (chr) of *Gigantopelta aegis*.

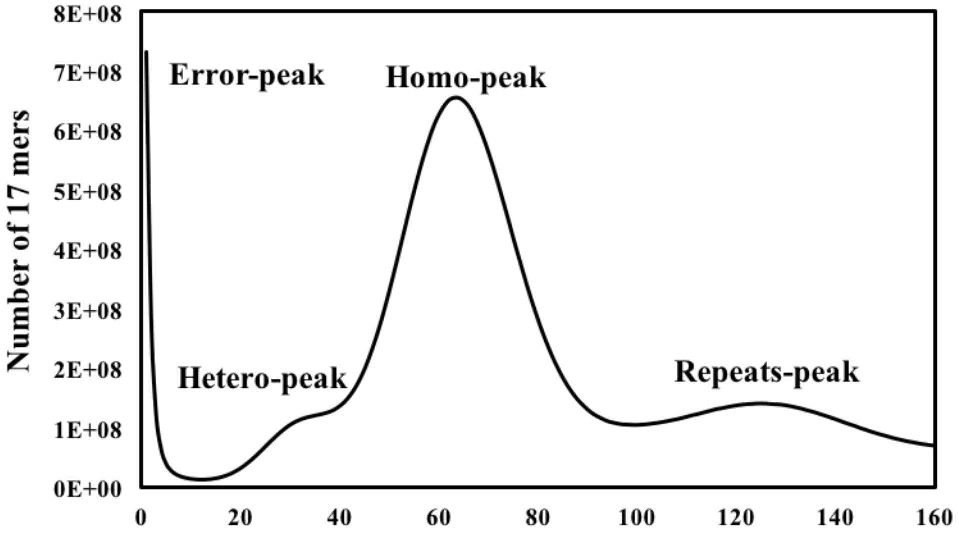

Supplementary Figure 19 The 17-mer distribution histogram of *Gigantopelta aegis* genome. Source data are provided in a Source Data file.

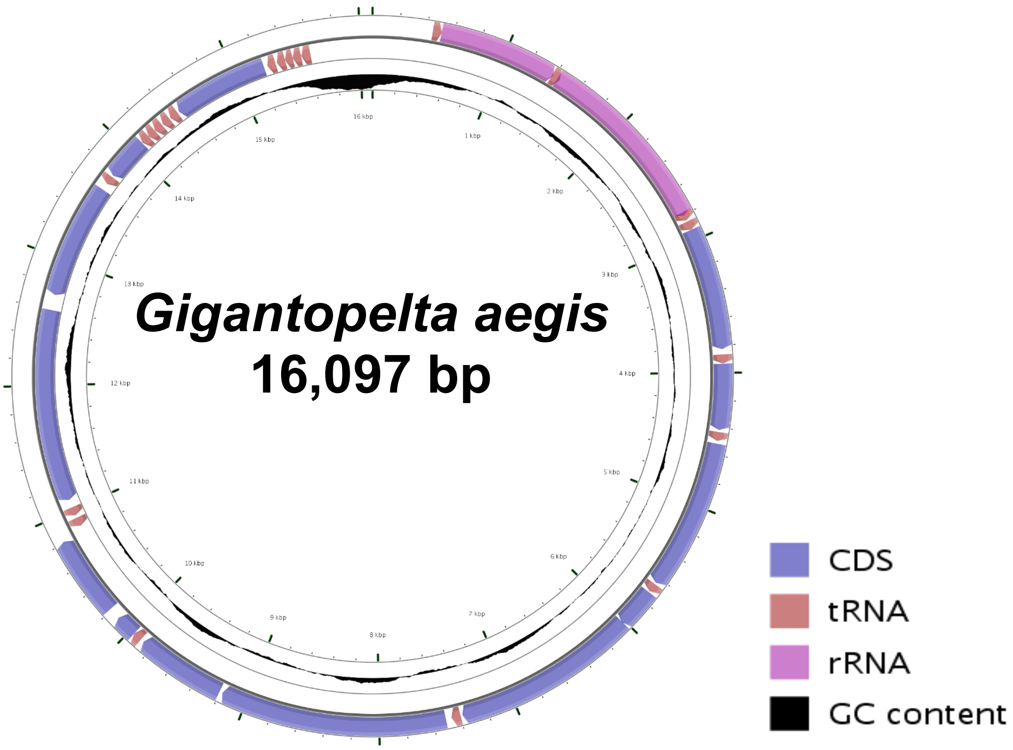

Supplementary Figure 20 The circos plot of the mitochondrial genome of *Gigantopelta aegis*. Inner ring: GC content; outer ring: genes order. CDS: coding DNA sequence.

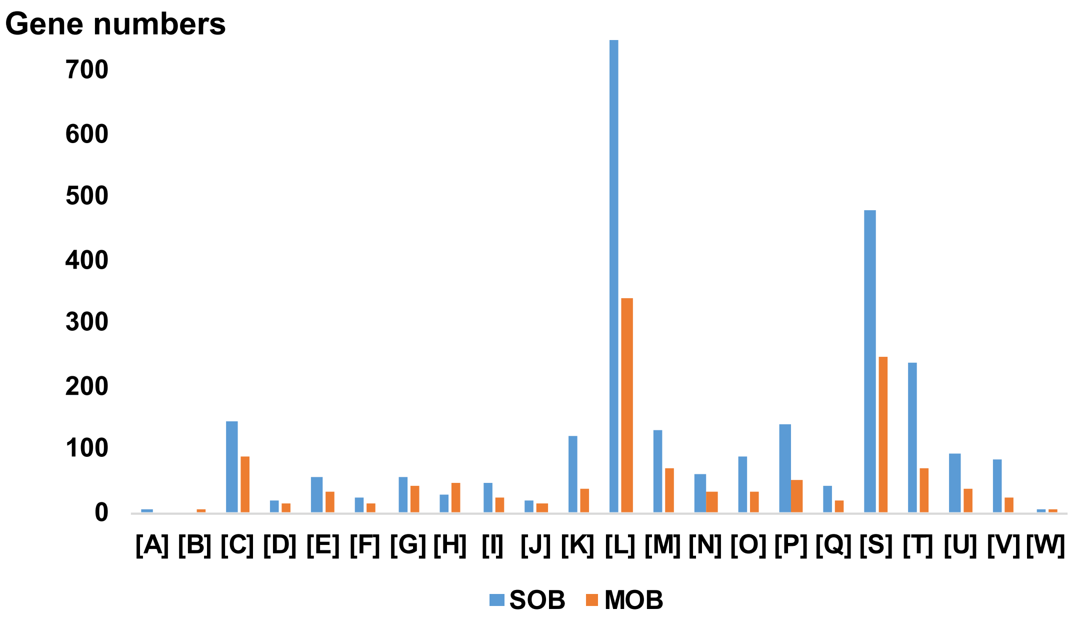

[A]	RNA processing and modification
[B]	Chromatin structure and dynamics
[C]	Energy production and conversion
[D]	Cell cycle control, cell division, chromosome partitioning
[E]	Amino acid transport and metabolism
[F]	Nucleotide transport and metabolism
[G]	Carbohydrate transport and metabolism
[H]	Coenzyme transport and metabolism
[I]	Lipid transport and metabolism
[J]	Translation, ribosomal structure and biogenesis
[K]	Transcription
[L]	Replication, recombination and repair
[M]	Cell wall/membrane/envelope biogenesis
[N]	Cell motility
[O]	Post-translational modification, protein turnover, and chaperones
[P]	Inorganic ion transport and metabolism
[Q]	Secondary metabolites biosynthesis, transport, and catabolism
[S]	Function unknown
[T]	Signal transduction mechanisms
[U]	Intracellular trafficking, secretion, and vesicular transport
[V]	Defense mechanisms
[W]	Extracellular structures

Supplementary Figure 21 The Clusters of Orthologous Groups (COG) annotation of the sulphur-oxidising bacteria and methane-oxidising bacteria of *Gigantopelta aegis*. One-letter abbreviation for the functional category was determined by the COG database. SOB: blue columns; MOB: orange columns. Source data are provided in a Source Data file.

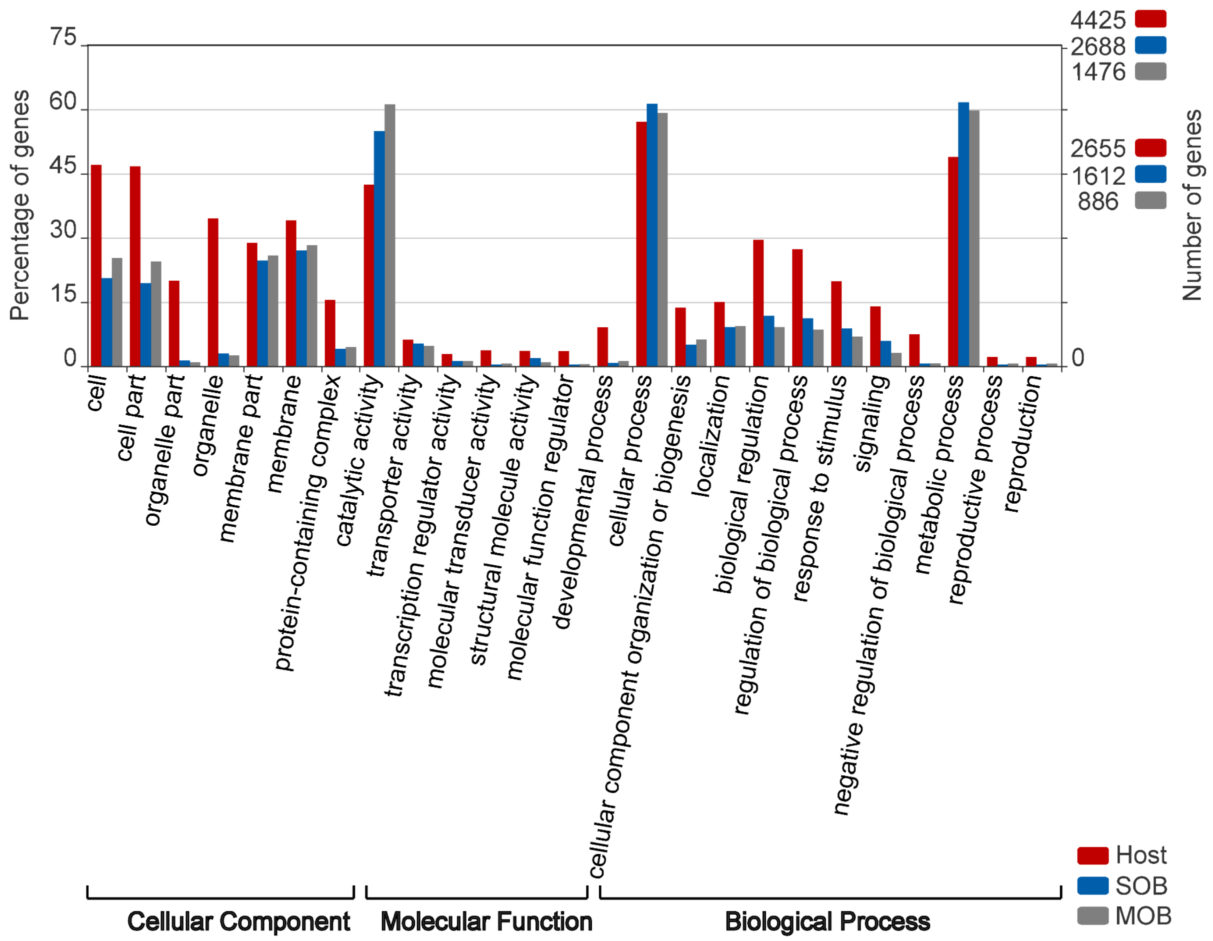

Supplementary Figure 22 A distribution plot shows the Gene Ontology (GO) items of the *Gigantopelta aegis* host (red), the sulphur-oxidising symbionts (SOB, blue), and the methane-oxidising symbionts (MOB, grey).

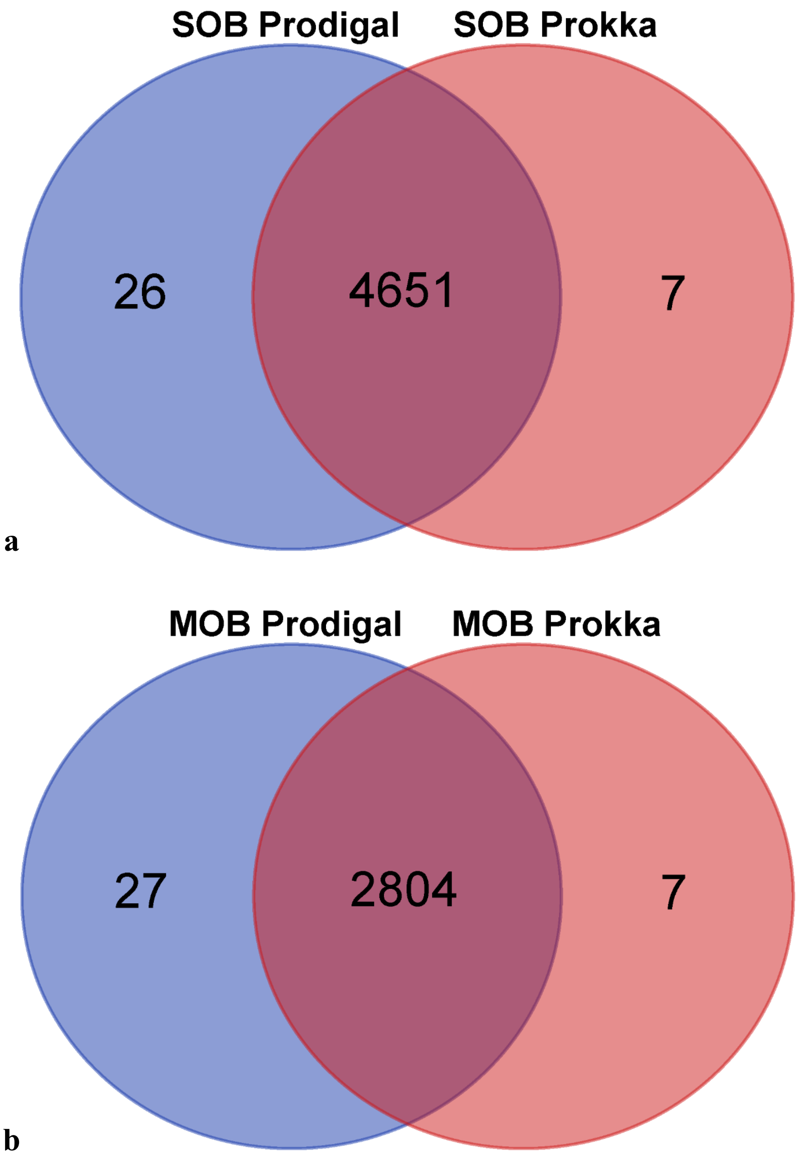

a

b

Supplementary Figure 23 Two venn diagrams show the shared gene family numbers between the genes predicted by Prodigal and Prokka of *Gigantopelta aegis* **a.** sulphur-oxidising symbionts SOB and **b**. methane-oxidising symbionts MOB, respectively. Purple: share gene family numbers between genes predicted by Prodigal (blue) and Prokka (pink). Source data are provided in a Source Data file.

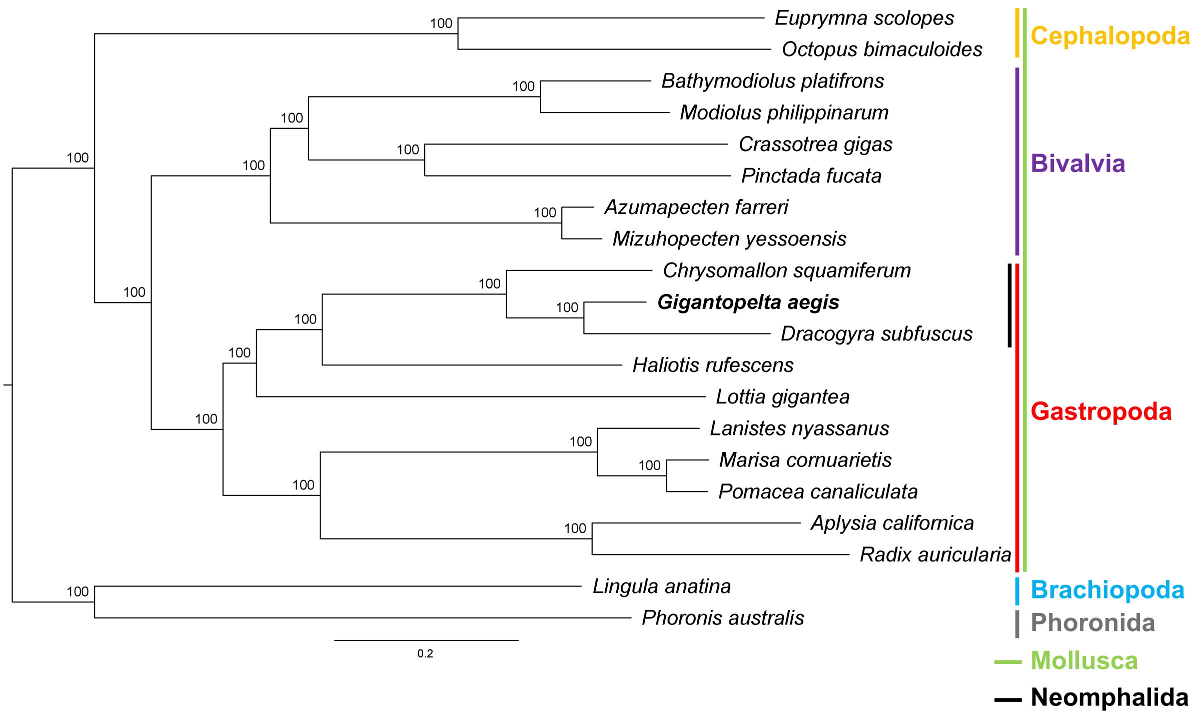

Supplementary Figure 24 A phylogenetic tree of Neomphalida including *Gigantopelta aegis*, *Dracogyra subfuscus* and *Chrysomallon squamiferum*, as well as other lophotrochozoan references. The references are listed as follows: *Aplysia californica* (GenBank No. GCA_000002075), *Bathymodiolus platifrons*, *Modiolus philippinarum*, *Azumapecten farreri*, *Crassotrea gigas*, *Euprymna scolopes*, *Lanistes nyassanus*, *Marisa cornuarietis*, *Pomacea canaliculata*, *Lingula anatina*, *Lottia gigantea*, *Octopus bimaculoides*, *Phoronis australis*, *Pinctada fucata*, *Mizuhopecten yessoensis*, *Radix auricularia*, and *Haliotis rufescens*. Taxa colour: Cephalopoda: orange; Bivalvia: purple; Gastropoda: red; Brachiopoda: blue; Phoronida: grey; Mollusca: green; and Neomphalida: black.

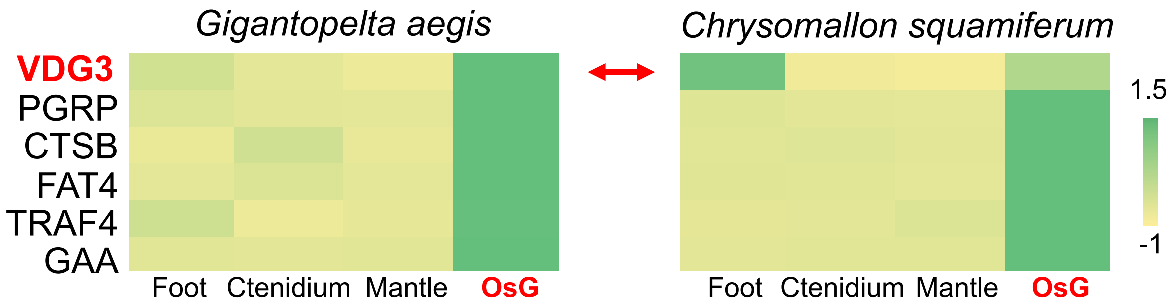

Supplementary Figure 25 The synteny blocks contain genes highly expressed in the oesophageal gland (OsG) of both *Gigantopelta aegis* (n = 4) and *Chrysomallon squamiferum* (n = 3). The colour represents the gene expression level (normalized CPM value). Green: high expression level. Yellow: low expression level. VDG3 (red) is highly expressed in the OsG of *G. aegis* but in the foot of *C. squamiferum*. The description of the protein names are provided in the Supplementary Note 8 Abbreviation List. Source data are provided in a Source Data file.

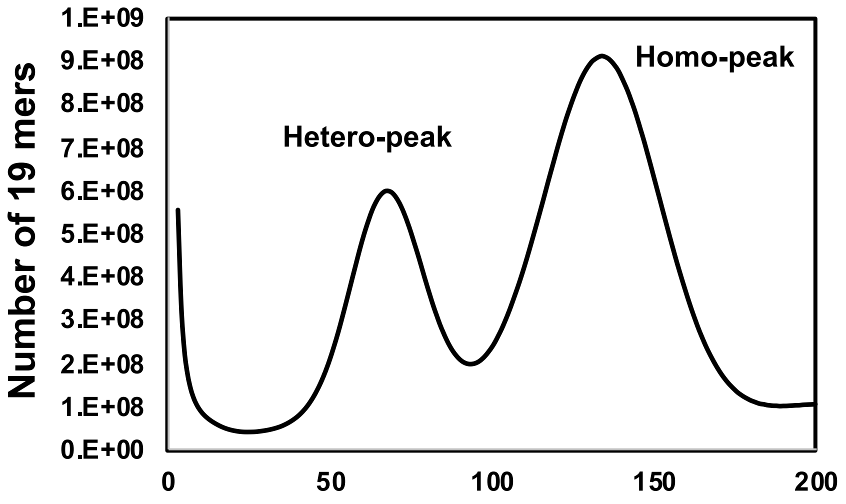

Supplementary Figure 26 The 19-mer distribution histogram of *Dracogyra subfuscus* genome. Source data are provided in a Source Data file.

Tables

Supplementary Table 1 Summary of the assembly statistics and functional annotation of *Gigantopelta aegis* genome. NR: non-redundant RefSeq protein database; GO: gene ontology database; KEGG: Kyoto encyclopedia of genes and genomes database; KOG: EuKaryotic Orthologous Groups.

Assembly Feature	Statistics
**15 pseudo-chromosomes (1.006 Gb) + 5,216 contigs (143.6 Mb)**	
Estimated genome size (17-mer analysis)	1.21 Gb
Number of scaffolds	5,231
Total assembly size (bp)	1,149,607,922
Longest scaffolds (nt)	120,622,278
N50 scaffold length (nt)	81,591,406
L50 scaffold count	6
N50 contig length (nt)	461,769
L50 contig count	671
Number of contigs	9,479
**15 pseudo-chromosomes (bp)**	1,005,966,341
Pseudo-chromosome 1 (nt)	47,317,128
Pseudo-chromosome 2 (nt)	54,566,076
Pseudo-chromosome 3 (nt)	96,789,575
Pseudo-chromosome 4 (nt)	120,622,278
Pseudo-chromosome 5 (nt)	40,821,028
Pseudo-chromosome 6 (nt)	102,884,749
Pseudo-chromosome 7 (nt)	47,477,099
Pseudo-chromosome 8 (nt)	81,591,406
Pseudo-chromosome 9 (nt)	84,917,595
Pseudo-chromosome 10 (nt)	91,665,651
Pseudo-chromosome 11 (nt)	35,397,884
Pseudo-chromosome 12 (nt)	53,390,639
Pseudo-chromosome 13 (nt)	42,253,053
Pseudo-chromosome 14 (nt)	59,883,994
Pseudo-chromosome 15 (nt)	46,388,186
**5,216 contigs (bp)**	143,641,581
Longest contig (nt)	741,762
N50 length (nt)	48,102
**Functional Annotation**	
Gene number predicted	21,438
NR	19,286 (90%)
GO	5,889 (27.5%)
KEGG	7,478 (34.9%)
KOG	13,543 (63.2%)
Pfam	19,135 (89.3%)

Supplementary Table 2 Classification and composition of repeats content in the genome of *Gigantopelta aegis* gastropod.

Repeats	Count	Length occupied	Percentage
DNA transposons	822	242470	0.02%
Academ	2581	914211	0.08%
CMC-Chapaev-3	222	153341	0.01%
Crypton	592	181221	0.02%
Ginger	751	227020	0.02%
IS	1	61	0.00%
Kolobok-Hydra	752	875613	0.08%
Maverick	5332	3187177	0.28%
MuLE-MuDR	297	142706	0.01%
P	1711	1179122	0.10%
PIF-Harbinger	823	312158	0.03%
PIF-ISL2EU	1091	461501	0.04%
Sola	4361	384141	0.03%
TcMar-Mariner	426	88052	0.01%
TcMar-Pogo	1887	753494	0.07%
TcMar-Tc1	26585	21697335	1.89%
hAT-Ac	2428	360679	0.03%
hAT-Tip100	1604	306676	0.03%
LINE	1921	793302	0.07%
CR1-Zenon	6990	1776404	0.15%
I	38938	5604071	0.49%
I-Nimb	29595	9949494	0.87%
Jockey	1618	1149642	0.10%
L1	2516	543469	0.05%
L1-Tx1	11441	6333774	0.55%
L2	1477	526978	0.05%
Penelope	21146	5362853	0.47%
Proto2	714	407527	0.04%
RTE-BovB	2185	457991	0.04%
RTE-X	22943	8511840	0.74%
**LTR**			
Copia	250	127009	0.01%
DIRS	4136	3573205	0.31%
Gypsy	14622	13102080	1.14%
Gypsy-Troyka	156	259429	0.02%
Ngaro	249	157130	0.01%
Low_complexity	49932	3156496	0.28%
Simple_repeat	653717	47311156	4.12%
Unknown	2333786	442290191	38.55%
**Total**	**3250598**	**582861019**	**50.80%**

Supplementary Table 3 Genome assembly results and functional annotation results of the sulphur-oxidising bacteria (SOB) and the methane-oxidising bacteria (MOB) housed in the oesophageal gland of *Gigantopelta aegis*. NR: non-redundant RefSeq protein database; GO: gene ontology database; KEGG: Kyoto encyclopedia of genes and genomes database; COG: clusters of orthologous groups.

	SOB	MOB
**Genome Assembly**		
Genome Size	4.91 Mb	2.93 Mb
Contigs Number	18	28
Scaffolds Number	11	10
Contamination	3.25%	1.67%
Completeness	98.55%	99.25%
Coverage	~ 3,050	~410
**Functional Annotation**		
Gene Number	5,518	3,102
NR	5,105 (92.5%)	3,019 (97.3%)
GO	3,584 (65.0%)	1,969 (63.5%)
KEGG	1,813 (32.9%)	1,525 (49.2%)
COG	4,405 (79.8%)	2,746 (88.5%)

Supplementary Table 4 The function of immunity-related gene family that are expanded in the *Gigantopelta aegis* genome. Two-tailed Fisher's exact test and false discovery rate adjustments was applied in the statistics analysis.

**Description**	**Gene Family**	**Function**	**Corrected *P* Value**
5-hydroxytryptamine receptor 4	HTR4	Immune	7.32E-04
BTB/POZ domain-containing protein 6	BTBD6	Suppress the programmed cell death in innate immune response[58]	8.10E-10
Carcinoembryonic antigen-related cell adhesion molecule 5	CEACAM5	Immune intracellular receptors[59]	7.46E-12
E3 ubiquitin-protein ligase TRIM56	TRIM	Immune signaling molecules[59]	3.17E-04
E3 ubiquitin-protein ligase rnf213-alpha	E3	Immune signaling molecules[59]	1.17E-04
Inhibitor of apoptosis-like protein	IAP	Regulation of innate immunity, inflammation and apoptosis[60]	6.64E-59
KRAB-A domain-containing protein 2	KRBA2	Epigenetic control of adaptive immune[61]	9.97E-16
F-Lectin	FUCL	Recognition (Innate immunity against microbial invasion)[62]	5.01E-07
Multiple epidermal growth factor-like domains protein 10	MEGF	Recognition[63]	3.33E-03
Ankyrin repeat protein	ANK	Symbiosis factors[64]	2.59E-02

Supplementary Table 5 Methane oxidation pathways of *Gigantopelta aegis* MOB and its free-living relatives. EMP: Embden–Meyerhof–Parnas; EDD: Entner–Doudoroff; H4MPT: 5,6,7,8-Tetrahydromethanopterin.

	Methane monooxygenase (PmoCAB/PxmCAB)	Methanol dehydrogenase (XoxF/Mxal)	EMP pathway	EDD pathway	H4MPT pathway	Serine cycle
Gigantopelta aegis MOB	+	+	+	+	+	+
Methylomarinum vadi	+	+	+	+	+	
Methylomonas methanica	+	+	+	+	+	+
Methylomonas lenta	+	+	+	+	+	
Methylomonas koyamae	+	+	+	+	+	
Methylomonas denitrificans	+	+	+	+	+	
Methylomonas sp. W13	+	+	+		+	
Methylomarinum sp.	+	+	+			
Methylomicrobium alcaliphilum	+	+	+	+	+	+
Methylomicrobium buryatense	+	+	+	+	+	+
Methylobacter luteus	+	+	+	+	+	
Methylobacter marinus	+	+	+	+	+	
Methylobacter whittenburyi	+	+				
Methylosarcina fibrata	+	+	+	+	+	
Methylomicobium agile	+	+	+	+	+	
Methylomicobium album	+	+	+	+	+	
Methylosarcina lacus	+	+	+	+		
Methylicorpusculum oleiharenae	+	+	+	+		

Supplementary Table 6 Hydrogen oxidation capability of the Gammaproteobacterial symbionts in deep-sea invertebrate taxa. SOB: sulphur-oxidising bacteria; MOB: methane-oxidising bacteria.

	respiratory hydrogenase group 1 NiFe hydrogenase (*hyaABC*) / uptake hydrogenase *hupL*	regulatory hydrogenase group 2 NiFe sensory hydrogenase (*hoxBC*/*hupUV*)
Gigantopelta aegis SOB	+	
Alviniconcha kojimai/*strummeri* Gamma-1 SOB	+	+
Alviniconcha strummeri Gamma-Lau SOB	+	+
Ifremeria nautilei Ifr1 SOB	+	+
Chrysomallon squamiferum SOB	+	+
Bathymodiolus azoricus SOB	+	
Bathymodiolus puteoserpentis SOB	+	
Bathymodiolus septemdierum SOB	+	
Bathymodiolus thermophilus SOB	+	
Bathymodiolus brooksi SOB		
Bathymodiolus heckerae SOB		
Turneroconcha magnifica SOB		
Phreagena okutanii SOB		
Escarpia spicata SOB	+	
Galathealinum brachiosum SOB	+	
Lamellibrachia luymesi SOB	+	
Ridgeia piscesae SOB	+	
Seepiophila jonesi SOB	+	
Tevnia jerichonana SOB	+	
Riftia pachyptila SOB	+	
Paraescarpia echinospica SOB	+	
Gigantopelta aegis MOB	+	
Bathymodiolus azoricus MOB		
Bathymodiolus platifrons MOB	+	
Bathymodiolus puteoserpentis MOB	+	
Bathymodiolus sp. MOB	+	
Ifremeria nautilei MOB	+	
Iophon methanophila MOB		
Hymedesmia (Stylopus) methanophila MOB		

Supplementary Table 7 Nitrate respiration capability of the Gammaproteobacterial symbionts in deep-sea invertebrate taxa. SOB: sulphur-oxidising bacteria; MOB: methane-oxidising bacteria.

	nitrate/nitrite transporter	nitrate reductase				nitrite reductase		nitric oxide reductase	nitrous-oxide reductase
	Nrt	NarGHI	NarB	NapAB	NasAB	NirBD	NirK/S	NorBC	NosZ
Gigantopelta aegis host	+								
Gigantopelta aegis SOB	+			+	+	+	+	+	+
Gigantopelta aegis MOB	+	+				+	+	+	
Chrysomallon squamiferum SOB	+		+	+	+	+	+	+	+
Alviniconcha kojimai/strummeri Gamma-1 SOB	+			+	+	+	+	+	+
Alviniconcha strummeri Gamma-Lau SOB	+			+		+	+	+	+
Ifremeria nautilei Ifr1 SOB	+			+			+	+	+
Ifremeria nautilei MOB	+	+							
Bathymodiolus azoricus SOB	+	+				+	+	+	
Bathymodiolus puteoserpentis SOB	+	+							
Bathymodiolus septemdierum SOB	+	+							
Bathymodiolus thermophilus SOB	+	+							
Bathymodiolus brooksi SOB	+	+						+	
Bathymodiolus heckerae SOB								+	
Bathymodiolus azoricus MOB						+			
Bathymodiolus platifrons MOB	+	+				+	+		
Bathymodiolus puteoserpentis MOB	+	+				+	+		
Bathymodiolus sp. MOB						+			
Turneroconcha magnifica SOB	+	+			+	+			
Phreagena okutanii SOB	+	+				+			
Escarpia spicata SOB	+			+		+		+	
Galathealinum brachiosum SOB	+			+	+	+		+	
Lamellibrachia luymesi SOB	+	+				+		+	
Ridgeia piscesae SOB	+	+				+		+	
Seepiophila jonesi SOB	+	+				+		+	
Tevnia jerichonana SOB	+	+				+		+	
Riftia pachyptila SOB	+	+				+		+	
Paraescarpia echinospica SOB	+	+		+			+	+	+
Iophon methanophila MOB	+	+				+			
Hymedesmia (Stylopus) methanophila MOB	+	+				+			

Supplementary Table 8 The usage information of each individual of *Gigantopelta aegis*. TEM: transmission electron microscopy; FISH: fluorescence *in situ* hybridisation.

Individuals ID	Collecting time	Tissue	Sequencing (NCBI accession number) / Experiments	Analysis (NCBI accession number)
Ga01	2015 January	Foot	PacBio (SRR13108175); Illumina (SRR13108173)	Host genome assembly (GCA_016097555.1)
		Oesophageal gland	Nanopore (SRR13108172); Illumina (SRR13108171)	Symbionts genome assembly and genome binning (SOB: GCA_016097415.1; MOB: GCA_016097405.1)
		Oesophageal gland	Metatranscriptome (SRR13131406)	Gene expression of symbionts
		Oesophageal gland	Metaproteome	Protein abundance of holobiont
		Different dissected tissues see **Supplementary Table 11**	Eukaryotic transcriptome (SAMN16909871)	Gene expression of host
Ga02	2015 January	Different dissected tissues see **Supplementary Table 11**	Eukaryotic transcriptome (SAMN16905113)	Gene expression of host
Ga03	2015 January	Oesophageal gland	Metatranscriptome (SRR13131416)	Gene expression of symbionts
		Oesophageal gland	Metaproteome	Protein abundance of holobiont
		Different dissected tissues see **Supplementary Table 11**	Eukaryotic transcriptome (SAMN16909869)	Gene expression of host
Ga04	2015 January	Oesophageal gland	Metatranscriptome (SAMN16909874)	Gene expression of symbionts
		Oesophageal gland	Metaproteome	Protein abundance of holobiont
		Different dissected tissues see **Supplementary Table 11**	Eukaryotic transcriptome (SAMN16909873)	Gene expression of host
Ga05	2015 January	Oesophageal gland	TEM	Confirm the existence of endosymbionts
Ga06	2019 April	Foot	Hi-C (SRR13108174)	Scaffolding of host genome
Ga07	2019 April	Oesophageal gland	FISH	Confirm the identity of endosymbionts

Supplementary Table 9 A list of probes used in the fluorescence *in situ* hybridisation experiments.

Probe Name	Sequence
SOB1	5'-AGCATATTAAACTTGTACCC-3'
MOB1	5'-CGTGTGTTTTCCTCCCTTCT-3'
EUB338	5'-GCTGCCTCCCGTAGGAGT-3'
NON338	5'-ACTCCTACGGGAGGCAGC-3'

Supplementary Table 10 Genome sequencing data of *Gigantopelta aegis* host and *Dracogyra subfuscus*. PE: paired-end, SE: single-end.

	Size (Gb)	N50 (nt)	Longest (nt)	Reads Number
Gigantopelta aegis				
Illumina reads (350 bp insert PE)	-	-	-	399,295,800
Illumina reads (500 bp insert PE)	-	-	-	740,205,720
PacBio Subreads	121	10 K	109 K	17,718,439
PacBio Corrected Subreads	52	11 K	109 K	4,701,940
Hi-C raw reads	482	-	-	3,215,925,346
Hi-C valid reads	-	-	-	112,328,978
Dracogyra subfuscus				
Illumina reads (SE)	-	-	-	502,320,019
Illumina reads (500 bp insert PE)	-	-	-	1,082,377,190

Supplementary Table 11 Transcriptome sequencing data of four individuals of *Gigantopelta aegis* (Ga01 [male], Ga02 [female], Ga03 [female], Ga04 [female]) with different dissected tissues. The RNA of oesophageal gland was used to construct both eukaryotic library and bacterial library. meta: metatranscriptome sequencing.

Tissues in different individuals	Raw reads	Clean Reads	NCBI accession
Ga01 Cephalic tentacles	39,289,210	37,854,692	SRR13131414
Ga01 Ctenidium	40,755,872	39,275,400	SRR13131413
Ga01 Digestive gland	39,928,094	38,574,096	SRR13131412
Ga01 Foot	38,047,576	36,749,336	SRR13131411
Ga01 Gonad	42,979,606	41,510,252	SRR13131410
Ga01 Mantle internal	40,992,658	39,499,540	SRR13131409
Ga01 Mantle edge	38,131,842	36,773,324	SRR13131408
Ga01 Oesophageal gland	136,226,224	133,303,450	SRR13131407
Ga01 Oesophageal gland (meta)	67,484,722	65,380,774	SRR13131406
Ga02 Cephalic tentacles	47,429,082	46,646,900	SRR13131273
Ga02 Ctenidium	114,776,966	113,018,798	SRR13131272
Ga02 Digestive gland	46,056,578	45,353,184	SRR13131271
Ga02 Epipodial tentacles	46,924,072	46,101,318	SRR13131265
Ga02 Foot	51,564,116	50,777,676	SRR13131270
Ga02 Mantle	62,871,456	61,599,812	SRR13131268
Ga02 Nephridium	43,485,296	42,859,728	SRR13131269
Ga02 Oesophageal gland	101,244,992	99,021,890	SRR13131266
Ga02 Operculum	43,453,604	42,782,788	SRR13131267
Ga02 Testis	44,804,546	44,040,958	SRR13131264
Ga03 Auricle heart	40,209,102	38,544,626	SRR13131415
Ga03 Ventricle heart	61,665,090	58,928,864	SRR13131423
Ga03 Cephalic tentacles	38,765,348	37,040,658	SRR13131425
Ga03 Ctenidium	42,849,664	40,826,230	SRR13131424
Ga03 Epipodial tentacles	40,563,874	38,752,620	SRR13131422
Ga03 Foot	38,479,932	36,842,512	SRR13131421
Ga03 Ovary	45,663,166	44,454,748	SRR13131420
Ga03 Mantle internal	35,283,212	33,810,720	SRR13131419
Ga03 Mantle edge	40,839,008	39,100,904	SRR13131418
Ga03 Oesophageal gland	263,318,982	257,059,132	SRR13131417
Ga03 Oesophageal gland (meta)	71,018,768	67,281,746	SRR13131416
Ga04 Cephalic tentacles	45,453,406	42,340,348	SRR13131435
Ga04 Ctenidium	41,484,232	39,704,082	SRR13131434
Ga04 Digestive gland	50,399,428	48,287,006	SRR13131433
Ga04 Epipodial tentacles	41,337,662	38,922,138	SRR13131430
Ga04 Foot	43,180,126	40,837,562	SRR13131432
Ga04 Ovary	40,721,278	38,950,644	SRR13131431
Ga04 Mantle edge	40,583,218	38,694,406	SRR13131429
Ga04 Oesophageal gland	158,304,352	153,983,134	SRR13131428
Ga04 Oesophageal gland (meta)	77,014,174	73,210,926	SRR13131427
Ga04 Ventricle heart	39,465,714	37,728,210	SRR13131426

Supplementary Table 12 Genome assembly of *Gigantopelta aegis* host by different assembling pipelines.

Assembler	Total size	Contig number	N50	Longest contigs
Canu correction+wtdbg2	1.16 Gb	9,842	467 kb	5.4 Mb
Canu correction+ SMARTdenovo	1.12 Gb	7,995	260 Kb	1.8 Mb
SMARTdenovo	1.31 Gb	8,520	313 Kb	2.59 Mb
Minimap2+miniasm	1.48 Gb	18,745	183 Kb	1.38 Mb
Flye	1.26 Gb	13,214	292 Kb	2.63 Mb
Hybrid assembly (MaSuRca)	1.38 Gb	18,264	202 Kb	2.76 Mb

Supplementary Table 13 Available genomes of symbionts belonging to Gammaproteobacteria in deep-sea invertebrate taxa.

Species	Host	SOB	MOB	Sampling Habitat	NCBI Accession	Reference (Ref)
Annelida						
Escarpia spicata		+		deep-sea seep	QFXE00000000	Ref 65
Lamellibrachia luymesi	+	+		deep-sea seep	SDWI00000000, QFXD00000000	Ref 65, 66
Galathealinum brachiosum		+		deep-sea muddy sediments	QFXC00000000	Ref 65
Ridgeia piscesae		+		deep-sea vent	LDXT00000000	Ref 67
Riftia pachyptila		+		deep-sea vent	AFOC00000000	Ref 68
Seepiophila jonesi		+		deep-sea seep	QFXF00000000	Ref 65
Tevnia jerichonana		+		deep-sea vent	AFZB00000000	Ref 68
Osedax frankpressi		+		deep-sea whale fall	ASZJ00000000	Ref 69
Paraescarpia echinospica		+		deep-sea seep	RZUD00000000	Ref 70
Mollusca						
Bathymodiolus azoricus		+	+	deep-sea vent	CDSC00000000, FMJP00000000	Ref 71
Bathymodiolus platifrons	+		+	deep-sea seep	MJUT00000000.1, PRJDB5337	Ref 29, 72
Bathymodiolus thermophilus		+		deep-sea vent	CP024634.1	Ref 73
Bathymodiolus sp.			+	deep-sea vent	FNVW00000000	Ref 71
Bathymodiolus puteoserpentis		+	+	deep-sea vent	FQTQ00000000, UEXF00000000	-
Bathymodiolus brooksi		+		deep-sea vent	FQTS00000000	-
Bathymodiolus heckerae		+		deep-sea vent	FXLV00000000	-
Bathymodiolus septemdierum		+		deep-sea vent	AP013042.1	Ref 74
Turneroconcha magnifica		+		deep-sea vent	JARW00000000	Ref 75
Phreagena okutanii		+		deep-sea vent	AP009247.1	Ref 76
Chrysomallon squamiferum	+	+		deep-sea vent	PRJNA523462, AP012978.1	Ref 11, 51
Alviniconcha		+		deep-sea vent	RAST: 6666666.293770, 6666666.293769	Ref 77
Ifremeria nautilei		+	+	deep-sea vent	RAST: 6666666.293767, 6666666.296237	Ref 77
Gigantopelta aegis	+	+	+	deep-sea vent	PRJNA612619	This study
Porifera						
Iophon methanophila			+	deep-sea seep	PRJNA475442	Ref 78
Hymedesmia (Stylopus) methanophila			+	deep-sea seep	PRJNA475438	

Supplementary Table 14 Characterisation of *Gigantopelta aegis* mitogenome.

Gene name	Feature	Start	End	Length	Intergenic nucleotides (nt)	Start codon	Stop codon	Strand
trnM(atg)	tRNA	444	510	67	-2			+
rrnS	rRNA	509	1386	878	-8			+
trnV(gta)	tRNA	1379	1443	65	1			+
rrnL	rRNA	1445	2803	1359	-25			+
trnL1(cta)	tRNA	2779	2843	65	10			+
trnL2(tta)	tRNA	2854	2920	67	-3			+
nad1	gene	2918	3865	948	0	ATA	TAA	+
trnP(cca)	tRNA	3866	3932	67	1			+
nad6	gene	3934	4434	501	0	ATG	TAA	+
trnE(gaa)	tRNA	4435	4500	66	8			+
cob	gene	4509	5648	1140	3	ATG	TAA	+
trnS2(tca)	tRNA	5652	5716	65	8			+
nad4l	gene	5725	6024	300	80	ATA	TAG	+
nad4	gene	6105	7391	1287	8	ATG	TAA	+
trnH(cac)	tRNA	7400	7467	68	-3			+
nad5	gene	7465	9195	1731	11	ATA	TAA	+
cox2	gene	9207	9899	693	-2	ATG	TAA	+
trnD(gac)	tRNA	9898	9963	66	0			+
atp8	gene	9964	10128	165	0	ATG	TAA	+
atp6	gene	10129	10821	693	38	ATG	TAA	+
trnT(aca)	tRNA	10860	10931	72	20			-
trnF(ttc)	tRNA	10952	11019	68	32			-
cox1	gene	11052	12593	1542	4	ATG	TAA	-
nad2	gene	12598	13734	1137	-56	ATA	TAG	-
trnS1(agc)	tRNA	13679	13745	67	4			-
nad3	gene	13750	14103	354	0	ATG	TAA	-
trnI(atc)	tRNA	14104	14172	69	0			-
trnN(aac)	tRNA	14173	14238	66	2			-
trnR(cgt)	tRNA	14241	14310	70	8			-
trnA(gca)	tRNA	14319	14385	67	10			-
trnK(aaa)	tRNA	14396	14464	69	11			-
cox3	gene	14476	15255	780	4	ATG	TAG	-
trnG(gga)	tRNA	15260	15325	66	12			-
trnY(tac)	tRNA	15338	15404	67	0			-
trnC(tgc)	tRNA	15405	15466	62	0			-
trnW(tga)	tRNA	15467	15533	67	2			-
trnQ(caa)	tRNA	15536	15608	73	932			-

Supplementary Note 8: Abbreviation List

1,3-BPG: 1,3-bisphosphoglycerate
2PG: 2-phosphoglycerate
3PG: 3-P-Glycerate
aceE: pyruvate dehydrogenase (acetyl-transferring), homodimeric type
aceF: dihydrolipoyllysine-residue acetyltransferase
acnB: bifunctional aconitate hydratase 2/2-methylisocitrate dehydratase
acsA: acetate-CoA ligase
adhA: zinc-binding alcohol dehydrogenase family protein
algC: phosphomannomutase/phosphoglucomutase
Antp: homeotic protein antennapedia
aprAB: adenylyl-sulfate reductase subunit alpha and beta
APS: adenylyl sulfate
ATP6: ATP synthase subunit 6
ATP8: ATP synthase synthase subunit 8
CBB: Calvin–Benson–Bassham
cbbML: ribulose 1,5-bisphosphate carboxylase small and large chain
coaA: type I pantothenate kinase
coaB: phosphopantothenate---cysteine ligase (CTP)
coaC: phosphopantothenoylcysteine decarboxylase
coaD: pantetheine-phosphate adenylyltransferase
coasy: phosphopantetheine adenylyltransferase / dephospho-CoA kinase
COB: cytochrome b.
COX1: Cytochrome c oxidase subunit 1
COX2: Cytochrome c oxidase subunit 2
COX3: Cytochrome c oxidase subunit 3
CTSB: cathepsin B
cysC: bifunctional sulfate adenylyltransferase/adenylylsulfate kinase
cysQ: 3'(2'),5'-bisphosphate nucleotidase
cysZ: sulfate transporter CysZ
dsrAB: dissimilatory-type sulfite reductase subunit alpha and beta
EDD: Entner–Doudoroff
EMP: Embden–Meyerhof–Parnas
eno: phosphopyruvate hydratase
enpp1_3: ectonucleotide pyrophosphatase/phosphodiesterase family member 1/3
F1,6P: fructose 1,6-bisphosphate
F6P: fructose-6-phosphate
fae: formaldehyde-activating enzyme
FAT4: protocadherin Fat 4
fba: fructose-bisphosphate aldolase class II
fbp: class 1 fructose-bisphosphatase
fccA: cytochrome c4
fdhABC: formate dehydrogenase complex
fhs: formate--tetrahydrofolate ligase
ftr: formylmethanofuran--tetrahydromethanopterin N-formyltransferase
fumA: fumarate hydratase
G3P: glyceraldehyde-3-phosphate
G6P: glucose 6-phosphate
GAA: lysosomal alpha-glucosidase

GADP: glyceraldehyde 3-phosphate
gapA: type I glyceraldehyde-3-phosphate dehydrogenase
gck: glycerate kinase
glk: glucokinase
gltA: citrate synthase
glyA: serine hydroxymethyltransferase
gpmI: 2,3-bisphosphoglycerate-independent phosphoglycerate mutase
Hox1: homeobox protein 1
Hox2: homeobox protein 2
Hox3: homeobox protein 3
Hox4: homeobox protein 4
Hox5: homeobox protein 5
hoxH: Ni/Fe hydrogenase subunit alpha
HP: Hydroxypyruvate
hxlA: 3-hexulose-6-phosphate synthase
hxlB: 6-phospho 3-hexuloisomerase
icd: isocitrate dehydrogenase
ilvBGI: acetolactate synthase I/II/III large subunit
ilvC: ketol-acid reductoisomerase
ilvD: dihydroxy-acid dehydratase
Lox2: homeobox protein *Lox2*
Lox4: homeobox protein *Lox4*
Lox5: homeobox protein *Lox5*
lpdA: dihydrolipoyl dehydrogenase
mch: methenyltetrahydromethanopterin cyclohydrolase
mcl: CoA ester lyase
mdh: malate dehydrogenase
metF: methylenetetrahydrofolate reductase
mtdA: methylenetetrahydrofolate dehydrogenase
mtdB: methylenetetrahydromethanopterin dehydrogenase
mtkB: malate-CoA ligase subunit beta
mxaFI: methanol dehydrogenase
napAB: periplasmic nitrate reductase complex
narGHI: nitrate reductase subunit alpha, beta and gamma
nasA: nitrate reductase
ND1: NADH dehydrogenase subunit 1
ND2: NADH dehydrogenase subunit 2
ND3: NADH dehydrogenase subunit 3
ND4: NADH dehydrogenase subunit 4
ND4L: NADH dehydrogenase subunit 4L
ND5: NADH dehydrogenase subunit 5
ND6: NADH dehydrogenase subunit 6
nifJ: pyruvate ferredoxin oxidoreductase
nirBD: nitrite reductase small and large subunits
nirK: nitrite reductase, copper-containing
nirS: nitrite reductase
norB: nitric-oxide reductase
norC: cytochrome c
nosZ: cytochrome C
nrt: MFS transporter

OG: oxoglutarate
panB: 3-methyl-2-oxobutanoate hydroxymethyltransferase
panC: pantoate--beta-alanine ligase
panE: 2-dehydropantoate 2-reductase
PAPS: 3'-phosphoadenylyl sulfate
pdhD: dihydrolipoyl dehydrogenase
PEP: phosphoenolpyruvate
pfk/*pfkA*: 6-phosphofructokinase
pfp: pyrophosphate--fructose-6-phosphate 1-phosphotransferase
pgi: glucose-6-phosphate isomerase
pgk: phosphoglycerate kinase
pgm: alpha-D-glucose phosphate-specific phosphoglucomutase
PGRP: peptidoglycan-recognition protein
pmoABC: methane monooxygenase/ammonia monooxygenase subunit A, B and C
Post1: homeobox protein *Post1*
Post2: homeobox protein *Post2*
ppc: phosphoenolpyruvate carboxylase
prkB: phosphoribulokinase
pyk/*pykA*: pyruvate kinase
R5P: ribose-5-P
rpe: ribulose-phosphate 3-epimerase
rpiA: ribose-5-phosphate isomerase RpiA
Ru5P: ribulose-5-P
RuBP: ribulose-1,5-bis-P
RuMP: ribulose monophosphate
sat: sulfate adenylyltransferase
sdhABCD: succinate dehydrogenase complex
sgaA: aminotransferase class V-fold PLP-dependent enzyme
soxA: sulfur oxidation c-type cytochrome SoxA
soxB: thiosulfohydrolase SoxB
soxYZ: thiosulfate oxidation carrier complex protein
sqr: pyridine nucleotide-disulfide oxidoreductase
sucAB: 2-oxoglutarate dehydrogenase complex
sucCD: succinate--CoA ligase subunit beta and alpha
TCA: tricarboxylic acid
tkt: transketolase
tpiA: triose-phosphate isomerase
TRAF4: TNF receptor-associated factor 4
tst: rhodanese-like thiosulfate sulfurtransferase
VDG3: developmentally-regulated vdg3
X5P: xylulose-5-P

Supplementary References

1. Kajitani, R. et al. Efficient *de novo* assembly of highly heterozygous genomes from whole-genome shotgun short reads. *Genome Res.* **24**, 1384–1395 (2014).
2. Koren, S. et al. Canu: scalable and accurate long-read assembly via adaptive k-mer weighting and repeat separation. *Genome Res.* **27**, 722–736 (2017).
3. Ruan, J. & Li, H. Fast and accurate long-read assembly with wtdbg2. *Nat. Methods* **17**, 155–158 (2019).
4. Li, H. Minimap and miniasm: fast mapping and *de novo* assembly for noisy long sequences. *Bioinformatics* **32**, 2103–2110 (2016).
5. Li, H. Minimap2: pairwise alignment for nucleotide sequences. *Bioinformatics* **34**, 3094–3100 (2018).
6. Kolmogorov, M., Yuan, J., Lin, Y. & Pevzner, P. A. Assembly of long, error-prone reads using repeat graphs. *Nat. Biotechnol.* **37**, 540–546 (2019).
7. Zimin, A. V. et al. The MaSuRCA genome assembler. *Bioinformatics* **29**, 2669–2677 (2013).
8. Wu, Y. W., Simmons, B. A. & Singer, S. W. MaxBin 2.0: an automated binning algorithm to recover genomes from multiple metagenomic datasets. *Bioinformatics* **32**, 605–607 (2015).
9. Dudchenko, O. et al. *De novo* assembly of the Aedes aegypti genome using Hi-C yields chromosome-length scaffolds. *Science* **356**, 92–95 (2017).
10. Durand, N. C. et al. Juicebox provides a visualization system for Hi-C contact maps with unlimited zoom. *Cell Syst.* **3**, 99–101 (2016).
11. Sun, J. et al. The scaly-foot snail genome and the ancient origins of biomineralised armour. *Nat. Commun.* **11**, 1657 (2020).
12. Parks, D. H., Imelfort, M., Skennerton, C. T., Hugenholtz, P. & Tyson, G. W. CheckM: assessing the quality of microbial genomes recovered from isolates, single cells, and metagenomes. *Genome Res.* **25**, 1043–1055 (2015).
13. Li, D., Liu, C. M., Luo, R., Sadakane, K. & Lam, T. W. MEGAHIT: an ultra-fast single-node solution for large and complex metagenomics assembly via succinct *de Bruijn* graph. *Bioinformatics* **31**, 1674–1676 (2015).
14. Bernt, M. et al. MITOS: improved *de novo* metazoan mitochondrial genome annotation. *Mol. Phylogenet. Evol.* **69**, 313–319 (2013).
15. Price, A. L., Jones, N. C. & Pevzner, P. A. *De novo* identification of repeat families in large genomes. *Bioinformatics* **21**, i351–i358 (2005).
16. Bao, Z. & Eddy, S. R. Automated *de novo* identification of repeat sequence families in sequenced genomes. *Genome Res.* **12**, 1269–1276 (2002).
17. Benson, G. Tandem repeats finder: a program to analyse DNA sequences. *Nucleic Acids Res.* **27**, 573–580 (1999).
18. Wootton, J. C. & Federhen, S. Statistics of local complexity in amino acid sequences and sequence databases. *Comput. Chem.* **17**, 149–163 (1993).
19. Kapitonov, V. V. & Jurka, J. A universal classification of eukaryotic transposable elements implemented in Repbase. *Nat. Rev. Genet.* **9**, 411–412 (2008).
20. Hubley, R. et al. The Dfam database of repetitive DNA families. *Nucleic Acids Res.* **44**, D81–D89 (2015).
21. Haas, B. J. et al. *De novo* transcript sequence reconstruction from RNA-Seq: reference

generation and analysis with Trinity. *Nat. Protoc.* **8**, 1494–1512 (2013).
22. Haas, B. J. et al. Improving the *Arabidopsis* genome annotation using maximal transcript alignment assemblies. *Nucleic Acids Res.* **31**, 5654–5666 (2003).
23. Kent, W. J. BLAT—the BLAST-like alignment tool. *Genome Res.* **12**, 656–664 (2002).
24. Li, W. & Godzik, A. Cd-hit: a fast program for clustering and comparing large sets of protein or nucleotide sequences. *Bioinformatics* **22**, 1658–1659 (2006).
25. Stanke, M., Diekhans, M., Baertsch, R. & Haussler, D. Using native and syntenically mapped cDNA alignments to improve *de novo* gene finding. *Bioinformatics* **24**, 637–644 (2008).
26. Cantarel, B. L. et al. MAKER: An easy-to-use annotation pipeline designed for emerging model organism genomes. *Genome Res.* **18**, 188–196 (2008).
27. Hyatt, D. et al. Prodigal: prokaryotic gene recognition and translation initiation site identification. *BMC Bioinformatics* **11**, 119 (2010).
28. Seemann, T. Prokka: rapid prokaryotic genome annotation. *Bioinformatics* **30**, 2068–2069 (2014).
29. Sun, J. et al. Adaptation to deep-sea chemosynthetic environments as revealed by mussel genomes. *Nat. Ecol. Evol.* **1**, 121 (2017).
30. Li, Y. et al. Scallop genome reveals molecular adaptations to semi-sessile life and neurotoxins. *Nat. Commun.* **8**, 1721 (2017).
31. Zhang, G. et al. The oyster genome reveals stress adaptation and complexity of shell formation. *Nature* **490**, 49–54 (2012).
32. Belcaid, M. et al. Symbiotic organs shaped by distinct modes of genome evolution in cephalopods. *Proc. Natl. Acad. Sci. USA* **116**, 3030–3035 (2019).
33. Sun, J. et al. Signatures of divergence, invasiveness, and terrestrialization revealed by four apple snail genomes. *Mol. Biol. Evol.* **36**, 1507–1520 (2019).
34. Gerdol, M., Luo, Y.-J., Satoh, N. & Pallavicini, A. Genetic and molecular basis of the immune system in the brachiopod *Lingula anatina*. *Dev. Comp. Immunol.* **82**, 7–30 (2018).
35. Simakov, O. et al. Insights into bilaterian evolution from three spiralian genomes. *Nature* **493**, 526–531 (2012).
36. Albertin, C. B. et al. The octopus genome and the evolution of cephalopod neural and morphological novelties. *Nature* **524**, 220–224 (2015).
37. Luo, Y. J. et al. Nemertean and phoronid genomes reveal lophotrochozoan evolution and the origin of bilaterian heads. *Nat. Ecol. Evol.* **2**, 141–151 (2018).
38. Takeuchi, T. et al. Bivalve-specific gene expansion in the pearl oyster genome: implications of adaptation to a sessile lifestyle. *Zool. Lett.* **2**, 3 (2016).
39. Wang, S. et al. Scallop genome provides insights into evolution of bilaterian karyotype and development. *Nat. Ecol. Evol.* **1**, 120 (2017).
40. Schell, T. et al. An annotated draft genome for *Radix auricularia* (Gastropoda, Mollusca). *Genome Biol. Evol.* **9**, 585–592 (2017).
41. Masonbrink, R. E. et al. An annotated genome for *Haliotis rufescens* (red abalone) and resequenced green, pink, pinto, black, and white abalone species. *Genome Biol. Evol.* **11**, 431–438 (2019).
42. Stöger, I. et al. The continuing debate on deep molluscan phylogeny: evidence for serialia (Mollusca, Monoplacophora + Polyplacophora). *Biomed. Res. Int.* **2013**, 407072 (2013).

43. Benton, M. J., Donoghue, P. C. J. & Asher, R. J. in *The Timetree of Life* (eds. S. Blair Hedges, S. & Kumar, S.) 35–86 (Oxford University Press, 2009).
44. Hayes, K. A. et al. Molluscan models in evolutionary biology: apple snails (Gastropoda: Ampullariidae) as a system for addressing fundamental questions. *Am. Malacol. Bull.* **27**, 47–59 (2009).
45. Jörger, K. M. et al. On the origin of Acochlidia and other enigmatic euthyneuran gastropods, with implications for the systematics of Heterobranchia. *BMC Evol. Biol.* **10**, 323 (2010).
46. Benton, M. J. et al. Constraints on the timescale of animal evolutionary history. *Palaeontol. Electron.* **18**, 1–106 (2015).
47. Chen, H. et al. The comprehensive immunomodulation of NeurimmiRs in haemocytes of oyster *Crassostrea gigas* after acetylcholine and norepinephrine stimulation. *BMC Genomics* **16**, 942 (2015).
48. Newton, I. L. & Bordenstein, S. R. Correlations between bacterial ecology and mobile DNA. *Curr. Microbiol.* **62**, 198–208 (2011).
49. Kleiner, M., Young, J. C., Shah, M., VerBerkmoes, N. C. & Dubilier, N. Metaproteomics reveals abundant transposase expression in mutualistic endosymbionts. *mBio* **4**, e00223-13 (2013).
50. Moran, N. A., & Plague, G. R. Genomic changes following host restriction in bacteria. *Curr. Opin. Genet. Dev.* **14**, 627–633 (2004).
51. Nakagawa, S. et al. Allying with armored snails: the complete genome of gammaproteobacterial endosymbiont. *ISME J.* **8**, 40–51 (2014).
52. Shipway, J. R. et al. Observations on the life history and geographic range of the giant chemosymbiotic shipworm *Kuphus polythalamius* (Bivalvia: Teredinidae). *Biol. Bull.* **235**, 167–177 (2018).
53. Wessel, D. M. & Flügge, U. I. A method for the quantitative recovery of protein in dilute solution in the presence of detergents and lipids. *Anal. Biochem.* **138**, 141–143 (1984).
54. Huang, Z. X. et al. Pyrosequencing of *Haliotis diversicolor* transcriptomes: insights into early developmental molluscan gene expression. *PLoS ONE* **7**, e51279 (2012).
55. He, T. F., Chen, J., Zhang, J., Ke, C. H. & You, W. W. *SARP19* and *vdg3* gene families are functionally related during abalone metamorphosis. *Dev. Genes. Evol.* **224**, 197–207 (2014).
56. Wallner, G., Amann, R. & Beisker, W. Optimizing fluorescent *in situ* hybridization with rRNA-targeted oligonucleotide probes for flow cytometric identification of microorganisms. *Cytometry* **14**, 136–143 (1993).
57. Amann, R., et al. Combination of 16S rRNA-targeted oligonucleotide probes with flow cytometry for analysing mixed microbial. *Appl. Environ. Microbiol.* **56**, 1919–1925 (1990).
58. Orosa, B. et al. BTB-BACK domain protein POB1 suppresses immune cell death by targeting ubiquitin E3 ligase PUB17 for degradation. *PLoS Genet.* **13**, e1006540 (2017).
59. Chen, H. et al. The comprehensive immunomodulation of NeurimmiRs in haemocytes of oyster *Crassostrea gigas* after acetylcholine and norepinephrine stimulation. *BMC Genomics* **16**, 942 (2015).

60. Berthelet, J. & Dubrez, L. Regulation of apoptosis by inhibitors of apoptosis (IAPs). *Cells* **2**, 163–187 (2013).
61. Santoni de Sio, F. R. Kruppel-associated box (KRAB) proteins in the adaptive immune system. *Nucleus* **5**, 138–148 (2014).
62. Chen, J., Xiao, S. & Yu, Z. F-type lectin involved in defense against bacterial infection in the pearl oyster (*Pinctada martensii*). *Fish Shellfish Immun.* **30**, 750–754 (2011).
63. Perović-Ottstadt, S. et al. A (1→ 3)-β-d-glucan recognition protein from the sponge *Suberites domuncula*: Mediated activation of fibrinogen-like protein and epidermal growth factor gene expression. *Eur. J. Biochem.* **271**, 1924–1937 (2004).
64. Thomas, T. et al. Functional genomic signatures of sponge bacteria reveal unique and shared features of symbiosis. *ISME J.* **4**, 1557–1567 (2010).
65. Li, Y., Liles, M. R. & Halanych, K. M. Endosymbiont genomes yield clues of tubeworm success. *ISME J.* **12**, 2785–2795 (2018).
66. Li, Y. et al. Genomic adaptations to chemosymbiosis in the deep-sea seep-dwelling tubeworm *Lamellibrachia luymesi*. *BMC Biol.* **17**, 91 (2019).
67. Perez, M. & Juniper, K. Insights into symbiont population structure among three vestimentiferan tubeworm host species at eastern Pacific spreading centres. *Appl. Environ. Microbiol.* **82**, 5197–5205 (2016).
68. Gardebrecht, A. et al. Physiological homogeneity among the endosymbionts of *Riftia pachyptila* and *Tevnia jerichonana* revealed by proteogenomics. *ISME J.* **6**, 766–776 (2012).
69. Goffredi, S. K. et al. Genomic versatility and functional variation between two dominant heterotrophic symbionts of deep-sea *Osedax* worms. *ISME J.* **8**, 908–924 (2014).
70. Yang, Y. et al. Genomic, transcriptomic, and proteomic insights into the symbiosis of deep-sea tubeworm holobionts. *ISME J.* **14**, 135–150 (2019).
71. Ponnudurai, R. et al. Metabolic and physiological interdependencies in the *Bathymodiolus azoricus* symbiosis. *ISME J.* **11**, 463–477 (2017).
72. Takishita, K. et al. Genomic evidence that methanotrophic endosymbionts likely provide deep-sea *Bathymodiolus* mussels with a sterol intermediate in cholesterol biosynthesis. *Genome Biol. Evol.* **9**, 1148–1160 (2017).
73. Ponnudurai, R. et al. Genome sequence of the sulphur-oxidising *Bathymodiolus thermophilus* gill endosymbiont. *Stand. Genomic Sci.* **12**, 50 (2017).
74. Ikuta, T. et al. Heterogeneous composition of key metabolic gene clusters in a vent mussel symbiont population. *ISME J.* **10**, 990–1001 (2016).
75. Newton, I. L. G. et al. The *Calyptogena magnifica* chemoautotrophic symbiont genome. *Science* **315**, 998–1000 (2007).
76. Kuwahara, H. et al. Reduced genome of the thioautotrophic intracellular symbiont in a deep-sea clam, *Calyptogena okutanii*. *Curr. Biol.* **17**, 881–886 (2007).
77. Beinart, R. A., Luo, C., Konstantinidis, K., Stewart, F. & Girguis, P. R. The bacterial symbionts of closely related hydrothermal vent snails with distinct geochemical habitats show broad similarity in chemoautotrophic gene content. *Front. Microbiol.* **10**, 1818 (2019).
78. Rubin-Blum, M. et al. Fueled by methane: deep-sea sponges from asphalt seeps gain their

nutrition from methane-oxidising symbionts. *ISME J.* **13**, 1209–1225 (2019).
