# Supplementary material for: Hologenome analysis reveals dual symbiosis in the deep-sea hydrothermal vent snail Gigantopelta aegis
Source: Nat Commun. 2021 Feb 19;12:1165. doi: 10.1038/s41467-021-21450-7 (PMC7895826; doi:10.1038/s41467-021-21450-7)
Supplement: Supplementary file 3 — Reporting Summary [file 41467_2021_21450_MOESM3_ESM.pdf]

nature research

Corresponding author(s): Pei-Yuan Qian

Last updated by author(s): Jan 7, 2021

Reporting Summary

Nature Research wishes to improve the reproducibility of the work that we publish. This form provides structure for consistency and transparency in reporting. For further information on Nature Research policies, see Authors & Referees and the Editorial Policy Checklist .

Statistics

For all statistical analyses, confirm that the following items are present in the figure legend, table legend, main text, or Methods section.

n/a	Confirmed	
☐	☒	The exact sample size (*n*) for each experimental group/condition, given as a discrete number and unit of measurement
☐	☒	A statement on whether measurements were taken from distinct samples or whether the same sample was measured repeatedly
☐	☒	The statistical test(s) used AND whether they are one- or two-sided<br>*Only common tests should be described solely by name; describe more complex techniques in the Methods section.*
☒	☐	A description of all covariates tested
☐	☒	A description of any assumptions or corrections, such as tests of normality and adjustment for multiple comparisons
☐	☒	A full description of the statistical parameters including central tendency (e.g. means) or other basic estimates (e.g. regression coefficient) AND variation (e.g. standard deviation) or associated estimates of uncertainty (e.g. confidence intervals)
☐	☒	For null hypothesis testing, the test statistic (e.g. *F*, *t*, *r*) with confidence intervals, effect sizes, degrees of freedom and *P* value noted<br>*Give P values as exact values whenever suitable.*
☒	☐	For Bayesian analysis, information on the choice of priors and Markov chain Monte Carlo settings
☒	☐	For hierarchical and complex designs, identification of the appropriate level for tests and full reporting of outcomes
☒	☐	Estimates of effect sizes (e.g. Cohen's *d*, Pearson's *r*), indicating how they were calculated

Our web collection on statistics for biologists contains articles on many of the points above.

Software and code

Policy information about availability of computer code

Data collection	Illumina Novaseq 6000 platform was used to collect reads of genome, transcriptome, metatranscriptome, and Hi-C sequencing. MinKNOW version 3.1.8 was used for the collection of Oxford Nanopore Technologies subreads. PacBio sequel platform was used to collect subreads generated by single-molecule real-time library.
Data analysis	The software tools used in the data analysis of the present study are listed as follows:<br><br>LAS X software version 3.0.13 (Leica Microsystems), MinKNOW version 3.1.8, Guppy version 2.1.3, Trimmomatic version 0.36, SPAdes version 3.13.0, Unicycler version 0.4.7, SSPACE-LongRead version 1.1, CheckM version 1.0.13, Canu version 1.7.1, wtdbg2 version 2.1, MaSuRCA version 3.2.6, Platanus version 1.2.4, Racon version 1.3.1, Pilon version 1.22, Bowtie2 version 2.3.5, SAMtools version 1.6, MaxBin version 2.2.5, HiC-Pro version 2.11.1, Juicer version 1.5, 3D de novo assembly (3D-DNA) pipeline version 180114, assemblathon_stats.pl (https://github.com/ucdavis-bioinformatics/assemblathon2-analysis/blob/master/assemblathon_stats.pl), BUSCO version 3.0.2, Prodigal version 2.6.3, Prokka version 1.14.6, RepeatModeler version 1.0.11, RepeatMasker version 4.0.8, MAKER version 2.31.10 and version 3.01.03, eggNOG-mapper v2, BLASTp version 2.2.31+, KEGG Automatic Annotation Server (KAAS), hmmscan version 3.2.1(http://hmmer.org/), Orthofinder version 2.3.3, MCMCtree in PAML version 4.9, CAFE 3, MUSCLE version 3.8.31, IQ-TREE multicore version 1.6.10, Kallisto version 0.45.1, MCScan, JCVI (https://github.com/tanghaibao/jcvi), LAST version 1080, Pathway Tools, Rscript version 3.6.0, Mascot version 2.3.0, Microsoft Excel 2016, SMARTdenovo, Minimap2 version 2.12, Flye version 2.3.3, MEGAHIT version 1.1.1, MITOS Web Server, Trinity version 2.8.3, PASA pipeline version 2.2.0, BLAT version 36x4, CD-HIT-EST version 4.6.8, miniasm version 0.3-r179, and Augustus version 3.2.3.<br><br>All computer commands, codes and intermediate files for the bioinformatics analyses are based on the available software listed in the Materials and Methods section, which are provided at https://github.com/ylan3740/Commands-for-hologenomic-analyses under the GNU General Public License v3.0.

For manuscripts utilizing custom algorithms or software that are central to the research but not yet described in published literature, software must be made available to editors/reviewers. We strongly encourage code deposition in a community repository (e.g. GitHub). See the Nature Research guidelines for submitting code & software for further information.

Data

Policy information about availability of data

All manuscripts must include a data availability statement. This statement should provide the following information, where applicable:

- Accession codes, unique identifiers, or web links for publicly available datasets
- A list of figures that have associated raw data
- A description of any restrictions on data availability

All sequencing data and assembly data of Gigantopelta aegis and its two symbionts were deposited to the National Centre for Biotechnology Information (NCBI) database under BioProject PRJNA612619 (https://www.ncbi.nlm.nih.gov/bioproject/PRJNA612619). Detailed data information of G. aegis holobiont were provided in Supplementary Table 8 and 11. All sequencing data and assembly data Dracogyra subfuscus were also deposited to the NCBI database under BioProject PRJNA680542 (https://www.ncbi.nlm.nih.gov/bioproject/PRJNA680542). Assembly data and genome annotation data of both G. aegis and D. subfuscus are available in figshare (DOI: 10.6084/m9.figshare.13317932 and https://doi.org/10.6084/m9.figshare.13317932.v1). The genome assembly and predicted gene models of G. aegis can also be found at MolluscDB (http://mgbase.qnlm.ac/page/download/download). The metaproteomic data of G. aegis are available in PRIDE via ProteomeXchange under the identifier PXD022852 (https://www.ebi.ac.uk/pride/archive/projects/PXD022852).

Publicly available datasets used in the study include the following: NCBI NR database (https://www.ncbi.nlm.nih.gov/refseq/), KEGG (https://www.genome.jp/kegg/), Repbase (https://www.girinst.org/repbase/), EuKaryotic Orthologous Groups (KOG) database (https://mycocosm.jgi.doe.gov/help/kogbrowser.jsf), Clusters of Orthologous Groups (COG) database (https://www.ncbi.nlm.nih.gov/COG/), Pfam database (https://pfam.xfam.org), RAST server (http://rast.theseed.org/), SwissProt database via UniProt (https://www.uniprot.org/), and the ecocyc database (https://ecocyc.org).

The source data underlying Figure 4, 6a, 6b, and 7, and Supplementary Figure 1, 10, 11, 12, 13, 14, 19, 21, 23, 25, and 26 are provided in a Source Data file.

Field-specific reporting

Please select the one below that is the best fit for your research. If you are not sure, read the appropriate sections before making your selection.

☐ Life sciences ☐ Behavioural & social sciences ☒ Ecological, evolutionary & environmental sciences

For a reference copy of the document with all sections, see nature.com/documents/nr-reporting-summary-flat.pdf

Ecological, evolutionary & environmental sciences study design

All studies must disclose on these points even when the disclosure is negative.

Study description	This study aimed to explore the symbiosis in a vent endemic but barely studied mollusc, Gigantopelta aegis (Peltospiridae), which can only be found in Longqi hydrothermal vent field on the Southwest Indian Ocean Ridge. For transmission electron microscopy and fluorescence in situ hybridisation experiments, one individual was used in each experiments. For genome sequencing, one individual was used for contig assembly and another one was used for Hi-C sequencing to further scaffolding the contigs into chromosomes. For transcriptome sequencing, a total of four individuals were used for gene expression analysis of the host, and three of them were used for metatranscriptome sequencing and metaproteome analysis. Another deep-sea snail Dracogyra subfuscus (Peltospiridae) was also collected from Longqi hydrothermal vent field. One individual was used for genome sequencing. The sequencing data was used for genome assembly, gene prediction, molecular clock analysis and microbes detection.
Research sample	Taxa: Gigantopelta aegis (Mollusca: Gastropoda: Peltospiridae). This species is gonochoristic. In the present study, samples with a shell length ranging from 30-40 mm were used (age estimate is not available), which are sexually matured adults. G. aegis snails were collected from Longqi hydrothermal vent field (37.7839°S, 49.6502°E; 2,761 m depth) on the South West Indian Ridge.<br>A total of 7 individuals of G. aegis snails (Ga01-Ga07) were used in the present study. Ga01 was used for host genome, symbiont genome, eukaryotic transcriptome, and metatranscriptome sequencing, as well as metaproteome analysis. Ga02 was used for eukaryotic transcriptome sequencing. Ga03 and Ga04 was used for eukaryotic transcriptome, and metatranscriptome sequencing, as well as metaproteome analysis. Ga05 was used for TEM experiments. Ga06 was used for Hi-C sequencing. Ga07 was used for FISH experiments. Details of the sample usage are provided in Supplementary Table 8 and 11.<br><br>Taxa: Dracogyra subfuscus (Mollusca: Gastropoda: Peltospiridae). This species is also gonochoristic. Samples with a shell length of around 5 mm was used (age estimate is not available). It was collected from Longqi hydrothermal vent field (37.7839°S, 49.6502°E; 2,761 m depth). Only one individual was used for genome sequencing.
Sampling strategy	Gigantopelta aegis snails were collected by the manned submersible HOV Jiaolong on-board the R/V Xiangyanghong 9 cruise 35II from Longqi hydrothermal vent field (37.7839°S, 49.6502°E; 2,761 m depth) on the South West Indian Ridge in January 2015. Snails were immediately flash-frozen in liquid nitrogen once they were recovered on the ship. They were then transferred to -80 °C until DNA and RNA extraction. Specimens of G. aegis for Hi-C sequencing and fluorescence in situ hybridization (FISH) experiments were collected also from Longqi ('Tiamat' chimney) during Leg 3 of the COMRA R/V Dayang Yihao expedition 52 in April 2019. Tissue from the foot was immediately dissected from one individual, cut up, washed in phosphate-buffered saline buffer, and stored at -80 °C until Hi-C library preparation. The oesophageal gland tissue was dissected from the same individual and fixed in 4% paraformaldehyde overnight, followed by dehydration through an ethanol series (20%, 40%, 60%, and 80%) for 15 minutes each and stored at -80 °C until fluorescence in situ hybridization (FISH) experiments. Samples for transmission electron microscopy (TEM) were

immediately fixed in 10% buffered formalin after recovery on-board the research vessel.

A single individual was used for genome sequencing and transcriptome sequencing of both host and symbionts, and another one was used for Hi-C sequencing of the host. Since these animals are wild rather than inbred, the usage of single individual for genome sequencing can help avoid the genomic heterozygosity of mix individuals. Three individuals were used for transcriptome and metatranscriptome sequencing as well as metaproteome analysis for replication purposes. One individual was used for FISH experiments, and another individual was used for TEM experiments. The sample size used was limited by the rare availability of these snails.

Dracogyra subfuscus snails were collected from Longqi hydrothermal vent field (37.7839°S, 49.6502°E; 2,761 m depth). Only one individual was used for genome sequencing. The samples were fixed in RNAlater and transferred to -80 °C freezer until use. The sample size was limited due to the rare availability of these snails.

Data collection	Gigantopelta aegis snails were collected by the manned submersible HOV Jiaolong on-board multiple deep-sea research expeditions of R/V Xiangyanghong 9 cruise 35II-DV94 and the COMRA R/V Dayang Yihao from Longqi hydrothermal vent field (37.7839°S, 49.6502°E; 2,761 m depth) on the South West Indian Ridge (Sampling details in "Sampling strategy" section above). And Dracogyra subfuscus snails were also collected by the submersible of the COMRA R/V Dayang Yihao from Longqi hydrothermal vent field (37.7839°S, 49.6502°E; 2,761 m depth). Data are recorded by Zhou Yadong, Sun Jin and Sun Yanan.
Timing and spatial scale	The R/V Xiangyanghong 9 cruise 35II was carried in January 2015. The COMRA R/V Dayang Yihao expedition 52 was in April 2019. The sampling time was determined by the assigned funding and ship time as well as the sea conditions during the cruise. Samples were collected from Longqi hydrothermal vent field (37.7839°S, 49.6502°E; 2,761 m depth) on the South West Indian Ridge.
Data exclusions	No data were excluded.
Reproducibility	We performed both transcriptome and proteome sequencing on the host and two symbionts of Gigantopelta aegis. Both results are similar and comparable.
Randomization	The samples were collected randomly in the field.
Blinding	Since the deep-sea sampling is a bit challenging and the snails population is very rare, only few samples are available to us. Thus, blinding was not possible in the present study. However, experiments and analysis were performed and cross-checked by different people in order to minimise the biases.

Did the study involve field work? ☒ Yes ☐ No

Field work, collection and transport

Field conditions	Deep-sea hydrothermal vent fields between 2,761 m in depth, dives of the manned submersible Jiaolong and ROV Sea Dragon III were carried out on days of calm sea condition.
Location	Longqi hydrothermal vent field (37.7839°S, 49.6502°E; 2,761 m depth) on the South West Indian Ridge
Access and import/export	Gigantopelta aegis snails for the genome and transcriptome sequencing were collected by the manned submersible HOV Jiaolong on-board the R/V Xiangyanghong 9 cruise 35II from Longqi hydrothermal vent field (37.7839°S, 49.6502°E; 2,761 m depth) on the Southwest Indian Ridge. Specimens of G. aegis for Hi-C sequencing and fluorescence in situ hybridisation (FISH) experiments as well as Dracogyra subfuscus snails for the genome sequencing were collected also from Longqi ('Tiamat' chimney) during Leg 3 of the COMRA R/V Dayang Yihao expedition 52nd. Research cruises and sampling were authorised and approved by the China Ocean Mineral Resources R&D Association (COMRA). Biological samples imported into Hong Kong was approved by Center for Health Protection of Hong Kong with the Ref. No of DH PHO/P5/323.
Disturbance	To minimise the disturbance of the ROV Sea Dragon III and HOV Jiaolong for sample collection as well as the disturbance of sample collection to the natural hydrothermal vent environment, the ROV and HOV was suspended in the water column besides the chimney, and only approached to the fauna when necessary. Care was also taken to not damage the natural habitat and also the chimney, Samples were only collected in one location with the largest number of individuals. No oversampling was allowed during the collection.

Reporting for specific materials, systems and methods

We require information from authors about some types of materials, experimental systems and methods used in many studies. Here, indicate whether each material, system or method listed is relevant to your study. If you are not sure if a list item applies to your research, read the appropriate section before selecting a response.

Materials & experimental systems

n/a	Involved in the study
x	Antibodies
x	Eukaryotic cell lines
x	Palaeontology
	x Animals and other organisms
x	Human research participants
x	Clinical data

Methods

n/a	Involved in the study
x	ChIP-seq
x	Flow cytometry
x	MRI-based neuroimaging

Animals and other organisms

Policy information about studies involving animals; ARRIVE guidelines recommended for reporting animal research

Laboratory animals	No laboratory animals were used in the study.
Wild animals	Gigantopelta aegis snails were collected by the manned submersible HOV Jiaolong on-board the R/V Xiangyanghong 9 cruise 35II from Longqi hydrothermal vent field (37.7839°S, 49.6502°E; 2,761 m depth) on the Southwest Indian Ridge. Snails were dissected and immediately flash-frozen in liquid nitrogen once they were recovered on the ship. They were then transferred to -80 °C until DNA and RNA extraction. Specimens of G. aegis for Hi-C sequencing and fluorescence in situ hybridisation (FISH) experiments as well as specimens of Dracogyra subfuscus for genome sequencing were collected also from Longqi ('Tiamat' chimney) during Leg 3 of the COMRA R/V Dayang Yihao expedition 52 in April 2019. Both snails were killed during the dissection. For G. aegis, tissue from the foot was immediately dissected from one individual, cut up, washed in phosphate-buffered saline buffer, and stored at -80 °C until Hi-C library preparation. The oesophageal gland tissue was dissected from the same individual and fixed in 4% paraformaldehyde overnight, followed by dehydration through an ethanol series (20%, 40%, 60%, and 80%) for 15 minutes each and stored at -80 °C until FISH experiments. Samples for transmission electron microscopy were immediately fixed in 10% buffered formalin after recovery on-board the research vessel. For D. subfuscus, the whole body tissue was fixed in RNAlater and transferred to -80 °C freezer until use.
Field-collected samples	After fixation, samples of Gigantopelta aegis and Dracogyra subfuscus were immediately placed into -80°C freezer alive or in 4% PFA, upon recovery on the ship.
Ethics oversight	As the species used (Gigantopelta aegis and Dracogyra subfuscus) are invertebrate gastropod molluscs, no ethical approval or guidance was required. No cephalopod samples were used in this study. Research cruises and their collecting activities were authorised and approved by the China Ocean Mineral Resources R&D Association (COMRA).

Note that full information on the approval of the study protocol must also be provided in the manuscript.
