# Supplementary material for: Hologenome analysis reveals dual symbiosis in the deep-sea hydrothermal vent snail Gigantopelta aegis
Source: Nat Commun. 2021 Feb 19;12:1165. doi: 10.1038/s41467-021-21450-7 (PMC7895826; doi:10.1038/s41467-021-21450-7)
Supplement: Supplementary file 4 — Description of Additional Supplementary Files [file 41467_2021_21450_MOESM4_ESM.pdf]

Description of Additional Supplementary Files

Supplementary Data 1 The functional annotation of genes in *Gigantopelta aegis* host.

Supplementary Data 2 A list of host genes highly expressed in the oesophageal gland of *Gigantopelta aegis*.

Supplementary Data 3 The functional annotation and gene expression of genes predicted by Prodigal and Prokka in the sulphur-oxidising endosymbionts of *Gigantopelta aegis*.

Supplementary Data 4 The functional annotation and gene expression of genes predicted by Prodigal and Prokka in the methane-oxidising endosymbionts of *Gigantopelta aegis*.

Supplementary Data 5 The identified proteins of *Gigantopelta aegis* holobiont in the metaproteome analysis (three individuals: Ga01, Ga03, Ga04; emPAI: normalized emPAI).

Supplementary Data 6 A summary of the transporters in *Gigantopelta aegis*.
