# Supplementary material for: Hologenome analysis reveals dual symbiosis in the deep-sea hydrothermal vent snail Gigantopelta aegis
Source: Nat Commun. 2021 Feb 19;12:1165. doi: 10.1038/s41467-021-21450-7 (PMC7895826; doi:10.1038/s41467-021-21450-7)
Supplement: Supplementary file 2 — Peer Review File [file 41467_2021_21450_MOESM2_ESM.pdf]

Reviewer #1 (Remarks to the Author):

This is a fascinating study that uses genomics, transcriptomics, and proteomics to make hypotheses about the nutritional interdependence of the hydrothermal vent snails C. squamiferum and G. aegis and their sulfur- and methane-oxidizing symbionts. The manuscript is well-written and easy to follow. I commend the authors for their excellent work.

My primary concerns are as follows:

Hydrogen oxidation may be an important driver of chemosynthesis in some hydrothermal vent symbionts that also oxidize sulfur (mussel, Alviniconcha, I. nautilei, maybe tubeworm symbionts). The authors should address this potential in their analysis of chemosynthesis in the SOB symbionts here. This includes identification of hydrogenases in the genomes, transcriptomes, and proteomes.

The combination of methane-oxidation pathways identified in the MOB here seems to be unique. But what about in free-living methane oxidizers? Has this combo of pathways been observed in any other MOB genome? Given that this is unique, further discussion outside of the symbionts is necessary.

The authors make some statements about how the symbionts may access the compounds they need for chemosynthesis. There is no known blood transporter for methane, and the only sulfide carrying blood compound that is well-characterized is found in tubeworms. Please explain in more detail how the conclusion about compounds carrying methane or sulfide through the blood is supported.

The analysis of TEs is not explained – how were TEs identified in the genomes assembled here and also in the genomes used in comparisons?

While these are high quality assemblies, they are not complete. It is important that the authors acknowledge that genes missing from their analyses may be simply missing from assemblies, not biologically missing.

Please provide more information about which individuals were used for what analysis – perhaps make a supplemental table that goes over how many individuals were collected, and which individuals and what tissues from each were used for each ‘omic analysis.

Bioproject is not available when searched on NCBI, so cannot assess whether data has been properly made available.

Specific concerns:

Line 31: "more than one symbiont" is unclear – more than one strain? Species? Phylotype? This information is not necessary here, and has the potential to lead reader astray.

Line 52: "harmony" seems to invoke intent – change to "symbiosis" or "association" or something similar.

Lines 102-103: Including the colors here reads strangely, perhaps just include this information in the figure caption only.

Line 172-175: Some Alviniconcha species appear to be highly specific to one phylotype of symbiont (for example, A. boucheti) and so this comparison is not adequate. Furthermore, I. nautilei is dominated by one monophyletic symbiont phylotype across a range of geographic locations, despite hosting its symbionts in its gill tissue. Please remove this statement.

Lines 236-237: Just saying a "gamma" SOB here may not be clear to the unfamiliar reader. Do the

authors mean gammaproteobacterial?

Line 310: Missing "of" between "lysis" and "symbiont cells" here.

Line 314-317: These symbiont genome assemblies are not closed – is it possible that this is a missing gene in the assembly but not in the real genome?

Lines 324-325: TE enrichment can also be a side-effect of a transition while becoming an obligate symbiont. This is discussed in the Supplemental but not here. Please acknowledge this possibility here.

Lines 351-355: This sentence is not clear. What is the key criterion?

Line 359: No information about how many individuals were used for these assemblies. Please include.

Lines 390-391: How were these FISH probes designed and tested?

Line 479: How were the symbiont genomes binned by coverage and GC? What software program was used to do this? I see a reference to another paper, but some broad info about what was done/used for this is necessary here.

Paragraph starting Line 487:
This information seems out of order with previous methodological info in paragraph starting Line 475. Please clarify where this fits into the pipeline of assembly and binning. Or is this in reference to the host assembly? If so, please clarify.

Figure 3B: the symbiont called "Alviniconcha kojimai SOB" is also sometimes dominant in Alviniconcha strummeri. For the Alviniconcha symbionts, it would be best to refer to their phylotype names "Gamma-1" and "Gamma-Lau" for consistency with previous literature. I would suggest something like "A. kojimai/strummeri Gamma-1 SOB" and "A. strummeri Gamma-Lau SOB" as labels instead.

Table S16: Please clarify which assembly you are comparing here – the host?

Figure S9: Why are some genes shown twice or more here – like TnP in the SOB?

Figure S10: Which tubeworm and mussel symbionts are the authors comparing their data to here? And why not comparison to Alviniconcha and Ifremeria symbionts, given that these are closer relatives to the G. aegis and C. squamiferum symbionts?

Figure S17: Please label the Y-axis here.

Reviewer #2 (Remarks to the Author):

The authors present exciting evidence that G. aegis contains a methane oxidizing symbiont (MOB), in addition to the sulphur oxidizing symbiont (SOB) that was known for this snail genus, albeit from another species (G. chessoia). The presence of the MOB has been speculated on based on preliminary 16S data, however metagenomic and imaging evidence was lacking. I particularily liked the authors aim to acquire high quality genomes for both symbionts and the host in an integrated metagenomic and metatranscriptomics approach, a feature rarely seen in symbiosis research. With all this exciting data, the authors still have quite some room for improvement in terms of quality of the analyses employed, presentation of data (figures, tables and supplement) as well as in the discussion of their finding in the context of recent advances in deep sea chemosynthetic symbiosis research. While the manuscript appears to cover a lot of metabolic details in this exciting symbiosis, some of the results

appear questionable and need better methodology to be substantiated. Even though some of the presented findings suggest possible and unique metabolic interactions between the snail and the symbionts that would be game changing, the larger, general impact of their findings on our understanding of intracellular symbiosis is not well addressed.

Major points

1)There is a discrepancy between the authors' claim of convergent evolution and their tree (Fig. 3a) that to me suggests that the two snail species have had a common, symbiotic ancestor (= no convergent evolution). The overall message of convergent evolution between G. aegis and C. squamiferum would have benefitted from a conceptual extension and comparison with other hydrothermal vent symbiosis (e.g. Bathymodiolus spp.) that are also known to harbour a highly similar dual symbiosis of methane-oxidising and sulphur-oxidising Gammaproteobacteria. The easy to do and in my opinion crucial comparison between these enigmatic snails from the Indian Ocean and the wealth of knowledge we already have from mussel hosts from the Mid-Atlantic Ridge would be a valuable addition to the manuscript. It would offer a very strong analytical background for convergent evolution compared to the two snail species alone that appear to be sister clades (Fig. 3a).

2)For my taste, the authors did not provide enough background on the symbiosis itself, e.g. the mode of transmission, which I interpreted to be horizontal, etc. There are many details about the symbiosis and the two organisms that the author would have at hands, but that the reader needs to infer from citations in the manuscript. In several cases, even that becomes frustrating because only insufficient references are provided to do not ensure that the reader has all the information they need.

3) There is a potentially very interesting aspect revealed by the genome analyses of the host and the symbionts that is not addressed in detail. From the host genome analysis, it seems that the mussel hosts can synthesise 13 essential amino acids including Methionine and Tyrosine. These two amino acids are generally considered 'essential' as they cannot be synthesised by most studied animals including many marine species, but also Homo sapiens. I suggest that the authors corroborate this point in their manuscript and possibly verify this observation. If this finding is novel, it should be highlighted in combination with the surprising lack of common amino acid synthesis pathways for the bacterial symbionts, e.g. for cysteine, but see below. 4) The symbiont genome analyses and metabolic reconstructions presented in Figure 6 are quite detailed and the incorporation of expression data is much appreciated. Strikingly, the results suggest that neither symbiont can synthetize vitamin B3 and B6 and amino acids M and Y, the SOB is additionally not capable of synthesising vitamin B5 and amino acid C (cysteine!). To my knowledge, such a pattern of biosynthesis gaps has not been observed in other chemosynthetic symbionts – not even in the reduced symbiont Ca. Ruthia magnifica, which is only lacking the synthesis of vitamin B12 (Roeselers et al. 2010; Standards in Genomic Sciences) or Ca. Riegeria (Jäckle et al. 2019; PNAS). Looking at the methodology for symbiont genome analyses, I would suppose that the authors need to get more evidence for the presence or absence of metabolic pathways in the symbionts. I would recommend using bacteria specific annotation pipelines such as Prokka in combination with e.g. Pathway Tools (http://bioinformatics.ai.sri.com/ptools/). The metabolism of the SOB and MOB should also be compared to the closest (free-living) relatives to investigate if the potential loss of genes for the missing amino acids and vitamins is unique to the symbionts or common in their respective phylogenetic grouping. This would provide reliable evidence for the nutritional dependency with the snail host. The general pattern observed in terrestrial and marine symbioses are hosts with very limited amino acid capabilities and versatile symbionts that are fully self-reliant and in addition can also serve the host. In special cases e.g. in many insects, this pattern is taken to the extreme opposite of what the authors present, where the symbionts complement the host for one or several essential amino acids, and the symbionts have lost all other amino acid synthesis pathways.

5) In my opinion, the Results and Discussion section does not give enough background information,

probably due to the authors aim to stay within the word count constraints. The arguments on functions of different proteins and the novelty of their findings do not have enough space to be comprehensible. With a short manuscript appropriate for Nature Communications, it would seem prudent to select the key findings and present confirmatory or obvious observations in a supplementary text section. The reader would benefit from having less results reported and in return more information on the individual findings. The current density of the manuscript seems crammed and at the same time superficial in parts.

6) Generally, the Supplementary Tables and Figures could be better structured to aid the reader, e.g. by streamlining them according to where they are referenced in the manuscript (e.g. line 190 ff). In the current form, the reader has to jump different Supplementary figures for some statements. I suppose there are better ways of merging these very helpful resources to make the data/information easier to find and comprehend. Please also invest in the figure legends, they are often missing critical information on abbreviations (e.g. Figure 4, where the abbreviations of the genes should be listed more clearly or figure S11 where there is not scale for the level of gene expression.

Specific comments:
Abstract:
Line 31: endosymbioses with more than one symbiont are just as common as those with only one symbiont and several marine examples are well researched - To only name three across different animal phyla: Bathymodiolus, Olavius, Trichoplax
Line 32: Dual symbiosis usually means two symbiont types and one host.
Line 41: An enhanced immune capacity that likely facilitates the possession of more than one type of symbiont is difficult to understand. In my opinion, if an animal can host multiple distinct symbionts, its immunity is at most adapted but not enhanced. It could even be interpreted as being compromised and not as restrictive as those of animal hosts that only harbor one symbiont type or no permanent microbiome at all.

Introduction:

Line 52: Not all microorganisms live in “harmony” with animals (e.g. parasites).

Line 60 and following: “Several species of annelids and molluscs, however, are capable of hosting multiple co-occurring endosymbionts” is not backed by references 7,8,9,10 as no annelids refs are included. Annelids with multiple symbiont lineages are e.g. shallow water gutless oligochaetes, but no refs for them are given

Line 61: This statements needs to be more specific to focus on chemosynthetic symbioses. Even there work on e.g. Riftia pachyptila in the deep sea or Paracatenula in the shallow water is missing, both explore the host and symbiont combined gene expression

Line 65: ‘Hologenome is key to understanding how the partners cooperate’ - This is an overstatement – in my opinion, the key is the specific expression of a small subset of genes on a cellular or organ scale

Line 69 why most extreme? Here but also elsewhere in the text - please try to avoid overstatements and self-advertisement, even of your study object. Focus rather on the interesting biology we see
Figure 1 15 pseudochromosomes are the basis of both panels b and c. The two figure panels could be combined and both shown linearly as c for easier access

Line 71: What do the autors mean by completely reliant on the host for the supply of methane and sulphide? Even though the symbionts are not in the gills, I would suppose that the gases can still diffuse in and out, right? Here, references and a schematic are missing to explain the current status of our understanding how this symbiosis functions.
Results and Discussion:

Line 107: The size difference between the two host genomes seems large – the authors report a genome difference of 1 GB which they explain with the large number of repetitive regions. Is this backed up with other data, e.g. estimates based on kmer spectral analysis using illumina short reads?

Line 115: How does the synteny between G. aegis and C. squamiferum compare with other Mollusc genomes (if available)? Is this strong chromosomal conservation and intra-chromosomal rearrangement common in gastropods or other invertebrate phyla when comparing genomes from different genera that have evolved separately for more than 100 million years?

Line 127: Not sure which symbiont is which – needs to be illustrated in figure. Also the outline of a bacteriocyte would help here. Supplement figures need more detailed labelling.

Line 130: How did the authors check symbiont abundance? Relative abundance in metagenomes?
Line 166: Related to the symbiont phylogenetic reconstruction (Figure 3b): It creates a distorted picture of 'purely symbiotic clades when presented as by the authors. I would ask to generate separate phylogenies and include as much of a diversity of the related bacteria as the taxonomic scope you are interested in. In this framework, maybe to the genus level would be a reasonable cutoff. Supplementary trees with 16S rRNA gene sequences would be helpful as well.
Line 166: I don't follow their line of argument. Based on the presented tree, how can the autors relate the "phylogeny" of the symbionts to horizontal acquisition and to host-selection and specificity?
Line 172: How do the snail symbionts compare to other chemosynthetic symbioses? There are also different host groups that live in the same habitat but acquire different gammaproteobacterial SOB, right? Do other studies also assume independent origins of endosymbiosis (= convergent evolution?)
Line 172: How is that a contrast? The symbionts in Alviniconcha are also inside the host so hosting symbionts "inside" does clearly not mean higher selectivity. I would caution against such simplifications – e.g. Zimmermann et al https://onlinelibrary.wiley.com/doi/full/10.1111/mec.13554 showed higher specificity of ectosymbionts compared to endosymbionts for the same genus of symbionts.

Line 195: The evidence for the function of galectin and fucolectin has not been studied in the reference provided by the authors. Wippler et al. (2016), reference 22 in the manuscript, only report the presence of these lectins in the transcriptome/proteome of the symbiotic worm Olavius algarvensis. To suggest a specific function as the authors do, other references and a more detailed analysis would be required. If it is not possible to provide more experimental evidence for the function of these lectins, the authors should be more cautionary with their statement.
Line 202: Highly expressed compared to what? Other snails, tissues or other genes in the transcriptomes?

Line 204: This part appears very speculative – what digestion process are the authors talking about, lysosomal digestion? Table S12 was difficult to grasp for me, as I would have expected a comparison/relation of the expression of the concerned genes to the rest of the transcriptomes. How do the authors rate "high expression"? A reference gene/pathway or normalization is missing.
Line 207: Why is it expected that environmental chemical compounds fluctuate in concentration? I

generally agree with this statement, but timeframes and environmental data would be helpful, maybe as a reference if they are already published?

Line 214-221: Interesting – I would love to know how widespread these proteins are across other environmental or symbiotic bacteria.

Line 231-233: The sentence was difficult for me. Could you please explain in simpler terms what is being utilised "respectively" by which symbiont.

Line 289: Genes for nitrate respiration are indeed found in methane-oxidising bacteria of Bathymodiolus spp. Mussels

Line 295: This is an example for my general critique about Supplementary figures and tables – I am missing information about the level of gene expression shown in the heatmap. What do the colours represent? Also, why are different "Sialin-like" genes expressed in the different tissues as shown with the heatmap. Or do the authors refer to transcripts/isoforms in this case?
Line 300-304: I was wondering how the SOB of C. squafimerum, that does not host another symbiont, obtain the lacking intermediates of the TCA cycle if not from the other symbiotic partner? Or does this host contain another symbiont other than the SOB? Or are the authors only referring to the host G. aegis? This is not clear for me as written.

Line 310: What are the authors referring to? There are only "Biological Modules" listed. I assume the authors link "host lysis symbiont cells" to the Biological Module "Lysosome". I would love to see more detailed information here. As it is written, the reader needs to infer/imagine what the authors are referring to and how they link the observed patterns to symbiont lysis. Also, there is an "of" missing.
Line 352: "not particularly closely related" is very subjective. Please provide the reader with a generally established framework as e.g. introduced in https://doi.org/10.1016/j.dsr2.2012.12.004. Compared to other hydrothermal species within the Mollusca, the two hosts are actually quite closely related. Yet the hosts are ~110 million years apart and both genera predate the current interpredation of a cenozoic radiation of extant vent fauna. Were they both symbiotic before the current radiation? Did they have a symbiotic last common ancestor? And what are their closest relatives that are non-symbiotic, could you estimate this as well and put the two symbiotic genera in perspective?

REVIEWER COMMENTS

Reviewer #1 (Remarks to the Author):

This is a fascinating study that uses genomics, transcriptomics, and proteomics to make hypotheses about the nutritional interdependence of the hydrothermal vent snails C. squamiferum and G. aegis and their sulfur- and methane-oxidizing symbionts. The manuscript is well-written and easy to follow. I commend the authors for their excellent work.

Response: We appreciate the overall encouraging comments from the reviewer.

My primary concerns are as follows:

Hydrogen oxidation may be an important driver of chemosynthesis in some hydrothermal vent symbionts that also oxidize sulfur (mussel, Alviniconcha, I. nautilei, maybe tubeworm symbionts). The authors should address this potential in their analysis of chemosynthesis in the SOB symbionts here. This includes identification of hydrogenases in the genomes, transcriptomes, and proteomes.

Response: We have followed the reviewer's suggestion to examine the capability of the symbiont genome for hydrogen utilisation. The presence and absence of hydrogenase genes in deep-sea symbiont genomes are summarised in **Supplementary Table 7**. Sulphur-oxidising symbionts (SOB) of some *Bathymodiolus* mussel species, provannid snails in genera *Alviniconcha* and *Ifremeria*, siboglinid tubeworms, as well as methane-oxidising symbionts (MOB) of some *Bathymodiolus* mussels and *Ifremeria* snails have group 1 NiFe hydrogenase (hyaABC) / uptake hydrogenase hupL, suggesting that hydrogen could be taken up and oxidised as additional energy sources in these symbionts. And thus, hydrogen oxidation may be an important driver of chemosynthesis in those deep-sea vent/seep symbionts, as you mentioned. We also identified hupL gene in the genome of both SOB and MOB of *Gigantopelta aegis* (**Fig. 8**). The hupL of both SOB and MOB were not highly expressed in the transcriptome level (**Fig. 8**). They were not found in our metaproteome data possibly due to the low abundance. However, the presence of hupL in the genome and the transcriptome indicates that the SOB and MOB of *Gigantopelta aegis* have the capability of utilising hydrogen as an additional energy resource. These results have been added in the "Energy Resources" of "Results and Discussion" in the main text (**see line 359-371 of main text**). We thank you for this important comment.

The combination of methane-oxidation pathways identified in the MOB here seems to be unique. But what about in free-living methane oxidizers? Has this combo of pathways been observed in any other MOB genome? Given that this is unique, further discussion outside of the symbionts is necessary.

Response: We have looked into this possibility by constructing a phylogenetic tree of *Gigantopelta aegis* MOB and its free-living relatives (**Supplementary Figure 15)**. The methane oxidation pathways of the free-living methane oxidisers were identified and summarised in **Supplementary Table 6**. The combination of methane-oxidation pathways found in the *G. aegis* MOB were also present in its free-living relatives within the family Methylomonadaceae, such as *Methylomonas methanica*, *Methylomicrobium alcaliphilum* and *Methylomicrobium buryatense*. As such, the versatile methane oxidation is likely to be unique among the symbionts of deep-sea vent species but is known from free-living MOB. This has been discussed and clarified in the main text (**see line 352-357 of main text**).

The authors make some statements about how the symbionts may access the compounds they need for chemosynthesis. There is no known blood transporter for methane, and the only sulfide carrying blood compound that is well-characterized is found in tubeworms. Please explain in more detail how the conclusion about compounds carrying methane or sulfide through the blood is supported.

Response: Indeed, haemoglobin in tubeworms and Lucinidae clams are known as carriers of sulphide and oxygen (Kraus & Wittenberg 1990; Minic & Hervé 2004), and these haemoglobins are closely related to some myoglobins that have oxygen binding capacity. Slight changes in the structures of myoglobin can enable them to have sulphide binding and transport capacities. Nevertheless, there is no known blood transporter for methane and sulphide in *Gigantopelta aegis*. The main evidence is a previous study on the anatomy of *G. aegis* which found a hypertrophied blood vascular system, which has been suggested to be an anatomical adaptation in order to transport essential dissolved compounds to the symbionts (Chen et al. 2017). According to our gene expression results for genes transporting oxygen (**Supplementary Figure 13**), one myoglobin, five neuroglobin, and one haemocyanin are highly expressed in the oesophageal gland. We therefore suspect that some these proteins may help transport sulphide. It is possible that methane is transported to the bacteriocytes through diffusion. However, this is speculative and needs physiological experiments to

verify, which is outside the scope of the present study. Therefore, we have toned this part down (**see line 83-89 of main text**).

Minic, Z. & Hervé, G. Biochemical and enzymological aspects of the symbiosis between the deep-sea tubeworm *Riftia pachyptila* and its bacterial endosymbiont. *Eur. J. Biochem*. **271**, 3093–3102 (2004).

Kraus, D. W. & Wittenberg, J. B. Hemoglobins of the *Lucina pectinata*/bacteria symbiosis. I. Molecular properties, kinetics and equilibria of reactions with ligands. *J. Biol. Chem*. **265**,16043–16053 (1990).

Chen, C. et al. By more ways than one: rapid convergence at hydrothermal vents shown by 3D anatomical reconstruction of *Gigantopelta* (Mollusca: Neomphalina). *BMC Evol. Biol.* **17**, 62 (2017).

The analysis of TEs is not explained – how were TEs identified in the genomes assembled here and also in the genomes used in comparisons?

Response: Among the TEs, only transposase genes and DNA transposon genes were analysed in the gene family analysis of the present study. The transposase genes and DNA transposon genes were identified in the gene prediction step using MAKER pipeline and functional annotation by searching NCBI Non-Redundant (NR) database via BLASTp. These genes in other molluscan genomes used in comparisons were found though identifying the orthologous groups shared between proteins of *Gigantopelta aegis* and those of molluscan genomes. The comparison results were generated from the gene family analysis. We have added a statement in the Methods “Gene Family and Phylogenomic analysis” section to clarify this procedure (**see line 677-680 of main text**).

While these are high quality assemblies, they are not complete. It is important that the authors acknowledge that genes missing from their analyses may be simply missing from assemblies, not biologically missing.

Response: We agree that there is a chance that the gene missing from our results may be caused by the incompleteness of genome assembly. However, the host genome and symbiont genomes from *G. aegis* and *C. squamiferum* were assembled using different sequencing platforms and different assembly pipelines respectively, which helped reduce technical bias of sequencing and assembly in the comparison analysis of nutrients biosynthesis capability. The lack of capability of biosynthesis of all nutrients (except for vitamin B_5 and coenzyme A)

are more likely to be biologically missing due to lacking more than one gene in the associated biosynthesis pathway. However, for the missing of one single gene *panE* missing in the SOB of two peltospirids, we acknowledged that the *panE* gene might be missing due to assembly error, although this was unlikely as these were missing from the genomes assembled using different sequencing platforms and assembly pipelines (*G. aegis* SOB: Nanopore sequencing and Illumina sequencing, Unicycler & SSPACE-LongRead; *C. squamiferum* SOB: 454 Roche sequencing and Sanger sequencing, GS De Novo Assembler; Nakagawa et al. 2014). The main text has been revised accordingly (**see line 414-421 of main text**).

Nakagawa, S. et al. Allying with armored snails: the complete genome of gammaproteobacterial endosymbiont. *ISME J.* **8**, 40–51 (2014).

Please provide more information about which individuals were used for what analysis – perhaps make a supplemental table that goes over how many individuals were collected, and which individuals and what tissues from each were used for each ‘omic’ analysis.

Response: A total of 7 individuals were used in the present study. This information has been provided in **Supplementary Table 9** and **Supplementary Table 11**.

Bioproject is not available when searched on NCBI, so cannot assess whether data has been properly made available.

Response: The data have already been deposited in the NCBI database. They will be released immediately once the manuscript is ready to be accepted.

Specific concerns:

Line 31: “more than one symbiont” is unclear – more than one strain? Species? Phylotype? This information is not necessary here, and has the potential to lead reader astray.

Response: It means “more than one symbiont phylotype”. This information is removed according to the reviewer’s suggestion (**see line 35-37 of main text**).

Line 52: “harmony” seems to invoke intent – change to “symbiosis” or “association” or something similar.

Response: It has been revised accordingly (**see line 56 of main text**).

Lines 102-103: Including the colors here reads strangely, perhaps just include this information in the figure caption only.
Response: The colour information has been removed from the sentence (**see line 132-134 of main text**). The colour information are kept in the figure caption (**Fig. 1a caption**).

Line 172-175: Some Alviniconcha species appear to be highly specific to one phylotype of symbiont (for example, A. boucheti) and so this comparison is not adequate. Furthermore, I. nautilei is dominated by one monophyletic symbiont phylotype across a range of geographic locations, despite hosting its symbionts in its gill tissue. Please remove this statement.
Response: This statement has been removed from the main text according to the reviewer's suggestion.

Lines 236-237: Just saying a "gamma" SOB here may not be clear to the unfamiliar reader. Do the authors mean gammaproteobacterial?
Response: Yes, "gamma" means gammaproteobacterial. The main text has been revised accordingly (**see line 308-310 of main text**).

Line 310: Missing "of" between "lysis" and "symbiont cells" here.
Response: It has been revised accordingly (**see line 409 of main text**).

Line 314-317: These symbiont genome assemblies are not closed – is it possible that this is a missing gene in the assembly but not in the real genome?
Response: Yes. It is possible, but the likelihood is small, given our high genome sequencing depth and the use of mainly long reads. Nevertheless, we acknowledge that the possibility that *panE* gene may be missing from the assembly, not the real genome, in the main text (**see line 414-421 of main text**).

Lines 324-325: TE enrichment can also be a side-effect of a transition while becoming an obligate symbiont. This is discussed in the Supplemental but not here. Please acknowledge this possibility here.
Response: We have moved some text from the Supplementary Material to acknowledge the possibility in the main text (**see line 435-438 of main text**).

Lines 351-355: This sentence is not clear. What is the key criterion?

Response: Biosynthesis capabilities of nutrients may be the key criterion for the selection of symbionts by the snail hosts. The main text has been revised accordingly (**see line 459-463 of main text**).

Line 359: No information about how many individuals were used for these assemblies. Please include.

Response: The usage information of each individual has been provided in **Supplementary Table 9**. For host genome assembly, the foot tissues of two individuals “Ga01” and “Ga06” were used for contig assembly and HiC scaffolding, respectively. For symbiont genome assembly, the oesophageal gland of the individual “Ga01” was used for genome assembly and genome binning.

Lines 390-391: How were these FISH probes designed and tested?

Response: The design of FISH probes for SOB and MOB was based on their 16S rRNA gene sequences, respectively. To make sure the 20 bp probe is unique and specific, the 16S rRNA gene sequence was searched against online standard databases of nucleotide collection and rRNA databases in NCBI. After confirming the specificity and uniqueness of the 20 bp sequence regions of SOB and MOB, 20 bp oligonucleotide probes that can 100% match the regions were synthesised, and their 3’ end were labeled with fluorescent dye Cy3 and Cy5, respectively. These details have been added in the main text Methods (**see line 497-504 of main text**).

A positive control probe and a negative control probe were used to test the specificity of the probes of symbionts in the same FISH protocol. The universal eubacterial probe EUB338 (5’-GCTGCCTCCCGTAGGAGT-3’; Amann et al. 1990) was used as the positive control for binding all bacteria and NON338 (5’-ACTCCTACGGGAGGCAGC-3’; Wallner et al. 1993) that is oligonucleotide complementary to EUB338 was used as the negative control for nonspecific binding. When applied the specific probes, EUB338 and NON338 in the same FISH experiment, the signal of NON338 is not detected (**Supplementary Figure 2h**) while the signal of EUB338 was significant (**Supplementary Figure 2i**). These methods and results together show the specificity and sensitivity of the designed probes. These details are included in the Supplementary Materials.

Amann, R. et al. Combination of 16S rRNA-targeted oligonucleotide probes with flow cytometry for analysing mixed microbial. *Appl. Environ. Microbiol.* **56**, 1919–1925 (1990).
Wallner, G., Amann, R. & Beisker, W. Optimizing fluorescent *in situ* hybridisation with rRNA-targeted oligonucleotide probes for flow cytometric identification of microorganisms. *Cytometry* **14**, 136–143 (1993).

Line 479: How were the symbiont genomes binned by coverage and GC? What software program was used to do this? I see a reference to another paper, but some broad info about what was done/used for this is necessary here.

Response: We followed the codes and commands used in the genome binning method reported in previous studies (Albertsen et al. 2013; Tian et al. 2016). All the commands and codes have been included in **Supplementary Data 8**. Briefly, the symbiont genome was assembled into contigs by SPAdes by using clean Illumina reads from oesophageal gland. These reads were mapped to the contigs by using Bowtie2 and SAMtools. The coverage of each contig was calculated by using “coverage_sum.pl” and “coverage_length.pl” (see “**Supplementary Data 8**”) based on the mapping depth. GC content was calculated by using “calc.gc.pl” (see “**Supplementary Data 8**”). These details and references have been added in the main text (**see line 596-600 of main text**).

Albertsen, M. et al. Genome sequences of rare, uncultured bacteria obtained by differential coverage binning of multiple metagenomes. *Nat. Biotechnol.* **31**, 533–538 (2013).
Tian, R. et al. The deep-sea glass sponge *Lophophysema eversa* harbours potential symbionts responsible for the nutrient conversions of carbon, nitrogen and sulphur. *Environ. Microbiol.* **18**, 2481–2494 (2016).

Paragraph starting Line 487:
This information seems out of order with previous methodological info in paragraph starting Line 475. Please clarify where this fits into the pipeline of assembly and binning. Or is this in reference to the host assembly? If so, please clarify.

Response: To address the reviewer’s concern, we have divided this section into two parts “Genome Assembly and Binning of Symbionts” and “Genome Assembly and Scaffolding of Host”. The pipelines used in the symbiont genome assembly and host genome assembly are different (**see line 589-630 of main text**).

Figure 3B: the symbiont called "Alviniconcha kojimai SOB" is also sometimes dominant in Alviniconcha strummeri. For the Alviniconcha symbionts, it would be best to refer to their phylotype names "Gamma-1" and "Gamma-Lau" for consistency with previous literature. I would suggest something like "A. kojimai/strummeri Gamma-1 SOB" and "A. strummeri Gamma-Lau SOB" as labels instead.

Response: We have followed the reviewer's suggestion regarding the use of these symbiont names throughout the whole manuscript.

Table S16: Please clarify which assembly you are comparing here – the host?

Response: **Supplementary Table 12** (formerly **Table S16**) shows the genome assembly results of the *Gigantopelta aegis* host obtained by using different assembly pipelines. We compared the results of different assemblies. The Table caption has been revised accordingly.

Figure S9: Why are some genes shown twice or more here – like TnP in the SOB?

Response: Because these genes, like TnP, have multiple copies (paralogs) in the genome.

Figure S10: Which tubeworm and mussel symbionts are the authors comparing their data to here? And why not comparison to Alviniconcha and Ifremeria symbionts, given that these are closer relatives to the G. aegis and C. squamiferum symbionts?

Response: Figure S10 has been removed from the manuscript. Instead, we summarised the nitrate respiration genes of almost all deep-sea vent/seep symbionts with a sequenced genomes (**Supplementary Table 8**). The symbionts of the deep-sea tubeworms (*Escarpia spicata*, *Galathealinum brachiosum*, *Lamellibrachia luymesi*, *Ridgeia piscesae*, *Seepiophila jonesi*, *Tevnia jerichonana*, *Riftia pachyptila*, *Paraescarpia echinospica*), mussels (*Bathymodiolus azoricus*, *Bathymodiolus puteoserpentis*, *Bathymodiolus septemdierum*, *Bathymodiolus thermophilus*, *Bathymodiolus brooksi*, *Bathymodiolus heckerae*, *Bathymodiolus platifrons*, *Bathymodiolus* sp.), gastropods (*Alviniconcha kojimai*, *Alviniconcha strummeri*, *Ifremeria nautilei*, *Chrysomallon squamiferum*), vesicomyid clams (*Calyptogena magnifica*, *Phreagena okutanii*) and sponges (*Iophon methanophila*, *Hymedesmia (Stylopus) methanophila*) are used in the nitrate respiration analysis. These symbionts also have the capability of nitrate respiration (**Supplementary Table 8**). The main text has been revised accordingly (**see line 378-380 of main text**).

Figure S17: Please label the Y-axis here.

Response: The Y-axis is "gene numbers". The label has been included in the **Supplementary Figure 21** (formerly Figure S17).

Reviewer #2 (Remarks to the Author):

The authors present exciting evidence that G. aegis contains a methane oxidizing symbiont (MOB), in addition to the sulphur oxidizing symbiont (SOB) that was known for this snail genus, albeit from another species (G. chessoia). The presence of the MOB has been speculated on based on preliminary 16S data, however metagenomic and imaging evidence was lacking. I particularily liked the authors aim to acquire high quality genomes for both symbionts and the host in an integrated metagenomic and metatranscriptomics approach, a feature rarely seen in symbiosis research. With all this exciting data, the authors still have quite some room for improvement in terms of quality of the analyses employed, presentation of data (figures, tables and supplement) as well as in the discussion of their finding in the context of recent advances in deep sea chemosynthetic symbiosis research. While the manuscript appears to cover a lot of metabolic details in this exciting symbiosis, some of the results appear questionable and need better methodology to be substantiated. Even though some of the presented findings suggest possible and unique metabolic interactions between the snail and the symbionts that would be game changing, the larger, general impact of their findings on our understanding of intracellular symbiosis is not well addressed.

Response: We appreciate the overall encouraging comments from the reviewer. To address the reviewer's concerns, 1) we have added a draft genome of a non-symbiotic peltospirid snail *Dracogyra subfuscus* also collected from Longqi, which is the sister genus of *Gigantopelta*. These data further support the finding that *D. subfuscus* doesn't rely on symbionts for nutrients. Together with previous published phylogenies with a larger taxon sampling, our results are in line with that *G. aegis* and *C. squamiferum* are unlikely to have a common symbiotic ancestor but have acquired the symbionts independently and convergently evolved. Early fossils of peltospirids exist from Early Cretaceous seeps already. and they were small-sized and unlikely to have been symbiotic. Therefore, the fossil evidence supports the two genera evolved symbiosis separately, after the current radiation of peltospirids began. 2) We have provided the nutrients biosynthesis pathways of both the free-living relatives of

symbionts and all the available mollusc genome in the molecular clock. 3) We have provided more background information in Introduction, Results and Discussion to make it clearer and ensure the readers get enough background information. 4) We have reorganised the supplementary materials to make it easier to follow. Details are provided in the below.

Major points

1)There is a discrepancy between the authors' claim of convergent evolution and their tree (Fig. 3a) that to me suggests that the two snail species have had a common, symbiotic ancestor (= no convergent evolution). The overall message of convergent evolution between G. aegis and C. squamiferum would have benefitted from a conceptual extension and comparison with other hydrothermal vent symbiosis (e.g. Bathymodiolus spp.) that are also known to harbour a highly similar dual symbiosis of methane-oxidising and sulphur-oxidising Gammaproteobacteria. The easy to do and in my opinion crucial comparison between these enigmatic snails from the Indian Ocean and the wealth of knowledge we already have from mussel hosts from the Mid-Atlantic Ridge would be a valuable addition to the manuscript. It would offer a very strong analytical background for convergent evolution compared to the two snail species alone that appear to be sister clades (Fig. 3a).

Response: The convergent evolution claim is based on previously published trees and anatomical data (Chen et al. 2017a; Chen et al. 2017b), which also include phylogeny with higher taxon sampling in Peltospiridae. We have now clarified this in the main text. Furthermore, to provide more support for this, we assembled a draft genome of *Dracogyra subfuscus* (**Supplementary Note 7**), a small peltospirid gastropod (in the same family with *G. aegis* and *C. squamiferum*). This gastropod also co-occurs with *G. aegis* and *C. squamiferum* in the Longqi field, and previous phylogeny (Chen et al. 2017b) suggested that it is the sister genus of *Gigantopelta*, but anatomy has suggested it does not host endosymbiotic bacteria and is instead a grazer. Early fossils of peltospirids exist from Early Cretaceous seeps (Kaim et al. 2014). and they were small-sized and unlikely to have been symbiotic. Therefore, the fossil evidence supports the two genera evolved symbiosis separately, after the current radiation of peltospirids began.

Although the newly assembled *Dracogyra* genome is very fragmented, it contains 5,986 predicted genes, and share 529 single-copy orthologs (each orthologous group including at least 12 species) with other lophotrochozoan genomes used in current phylogenetic and molecular clock analysis. These single-copy orthologs are used in the phylogenomic analysis

and molecular clock analysis (**Fig. 3**, **Supplementary Figure 24**). In line with previous morphology study (Chen et al. 2017b), our genome binning results (**Supplementary Figure 8**) of *D. subfuscus* shows that it indeed does not possess endosymbionts. Our phylogenomic analysis confirmed that *D. subfuscus* is much more closely related to *G. aegis* than *C. squamiferum* (**Fig. 3**, **Supplementary Figure 24**). These results further support the finding that *G. aegis* and *C. squamiferum* are unlikely to have a common symbiotic ancestor but acquire the symbiont independently and convergently evolved. The alternative hypothesis that *D. subfuscus* has lost its symbiont is unlikely supported, because most known genera of Neomphalina do not bear endosymbionts (Chen et al. 2017b).

Although a few *Bathymodiolus* mussels also can house dual symbionts of sulphur-oxidising symbionts and methane-oxidising symbionts, *Bathymodiolus* mussels are more likely to have a common symbiotic ancestor (Lorion et al. 2013; Miyazaki et al. 2010).

These are added and clarified in the main text (**see line 105-107, line 189-192 and line 212-225 of main text**).

Chen, C. et al. By more ways than one: rapid convergence at hydrothermal vents shown by 3D anatomical reconstruction of *Gigantopelta* (Mollusca: Neomphalina). *BMC Evol. Biol.* **17**, 62 (2017a).

Chen, C., Zhou, Y., Wang, C. & Copley, J. T. Two new hot-vent peltospirid snails (Gastropoda: Neomphalina) from Longqi hydrothermal field, Southwest Indian Ridge. *Front. Mar. Sci.* **4**, 392 (2017b).

Kaim, A., Jenkins, R. G., Tanabe, K. & Kiel, S. Mollusks from late Mesozoic seep deposits, chiefly in California. *Zootaxa* **3861**, 401–440 (2014).

Lorion, J. et al. Adaptive radiation of chemosymbiotic deep-sea mussels. *P. Roy. Soc. B-Biol. Sci.* **280**, 20131243 (2013).

Miyazaki, J. I., de Oliveira Martins, L., Fujita, Y., Matsumoto, H. & Fujiwara, Y. Evolutionary process of deep-sea *Bathymodiolus* mussels. *PLoS ONE* **5**, e10363 (2010).

2)For my taste, the authors did not provide enough background on the symbiosis itself, e.g. the mode of transmission, which I interpreted to be horizontal, etc. There are many details about the symbiosis and the two organisms that the author would have at hands, but that the reader needs to infer from citations in the manuscript. In several cases, even that becomes

frustrating because only insufficient references are provided to do not ensure that the reader has all the information they need.

Response: We have followed the reviewer's suggestion to include more background information about the transmission mode, symbiosis of *C. squamiferum* from symbiont side, the biology of the two snails, and associated references in the Introduction section of the main text.

3) There is a potentially very interesting aspect revealed by the genome analyses of the host and the symbionts that is not addressed in detail. From the host genome analysis, it seems that the mussel hosts can synthesise 13 essential amino acids including Methionine and Tyrosine. These two amino acids are generally considered 'essential' as they cannot be synthesised by most studied animals including many marine species, but also Homo sapiens. I suggest that the authors corroborate this point in their manuscript and possibly verify this observation. If this finding is novel, it should be highlighted in combination with the surprising lack of common amino acid synthesis pathways for the bacterial symbionts, e.g. for cysteine, but see below.

Response: To address the reviewer's concern, we have summarised the biosynthesis capability of amino acids of another 15 molluscs as well as two non-mollusc lophotrochozoans *Phoronis australis* (Phoronida) and *Lingula anatina* (Brachiopoda) (**Fig. 5**). Their capability of nutrients biosynthesis are highly conserved. All these species can synthesise methionine while except *Modiolus philippinarum* and *Phoronis australis*, the rest species can synthesise Tyrosine. These results have been added in the main text (**see line 402-403 of main text**).

4) The symbiont genome analyses and metabolic reconstructions presented in Figure 6 are quite detailed and the incorporation of expression data is much appreciated. Strikingly, the results suggest that neither symbiont can synthetize vitamin B3 and B6 and amino acids M and Y, the SOB is additionally not capable of synthesising vitamin B5 and amino acid C (cysteine!). To my knowledge, such a pattern of biosynthesis gaps has not been observed in other chemosynthetic symbionts – not even in the reduced symbiont Ca. Ruthia magnifica, which is only lacking the synthesis of vitamin B12 (Roeselers et al. 2010; Standards in Genomic Sciences) or Ca. Riegeria (Jäckle et al. 2019; PNAS). Looking at the methodology for symbiont genome analyses, I would suppose that the authors need to get more evidence for the presence or absence of metabolic pathways in the symbionts. I would recommend

using bacteria specific annotation pipelines such as Prokka in combination with e.g. Pathway Tools (http://bioinformatics.ai.sri.com/ptools/). The metabolism of the SOB and MOB should also be compared to the closest (free-living) relatives to investigate if the potential loss of genes for the missing amino acids and vitamins is unique to the symbionts or common in their respective phylogenetic grouping. This would provide reliable evidence for the nutritional dependency with the snail host. The general pattern observed in terrestrial and marine symbioses are hosts with very limited amino acid capabilities and versatile symbionts that are fully self-reliant and in addition can also serve the host. In special cases e.g. in many insects, this pattern is taken to the extreme opposite of what the authors present, where the symbionts complement the host for one or several essential amino acids, and the symbionts have lost all other amino acid synthesis pathways.

Response: We have followed the advice to predict the genes of both SOB and MOB using both Prodigal and Prokka, and results are highly consistent as indicated by Venn diagrams showing their shared gene families (**Supplementary Figure 23**). The functional annotation results of the genes predicted from Prokka are provided in **Supplementary Data 3** (**SOB**) and **Supplementary Data 4** (**MOB**). Prokka combined with Pathway Tools, the annotation of NCBI Non-Redundant database and KEGG database were all used to further check the capability of nutrient biosynthesis of the SOB and the MOB as well as their closest free-living relatives with available genomes (**Fig. 5**).

Previously, we mainly relied on the KEGG Mapper to reconstruct the nutrient pathways, which showed incomplete biosynthesis pathway of cysteine and methionine in the symbionts of both *Gigantopelta aegis* and *Chrysomallon squamiferum*. However, after further checking the annotation results of Pathway tools as well as using the annotated genes of the symbionts of *Phreagena okutanii* and *Calyptogena magnifica* (now *Turneroconcha magnifica*) (Kuwahara et al. 2007, Newton et al. 2007) as positive controls to further confirm the absence or presence of the biosynthesis pathways, we found that both the SOB and the MOB of *G. aegis* as well as the SOB of *Chrysomallon squamiferum* have complete pathways to synthesise all amino acids, including cysteine and methionine. But the SOB and the MOB do lack the complete pathways to synthesise vitamin B3 and vitamin B6. The SOB additionally lacks a complete pathway to synthesise vitamin B5 and coenzyme A. **Table 1** has been replaced and extended with a new **Fig. 5,** text have been corrected accordingly (**see line 403-406 of main text**).

We did compare the capability of nutrient biosynthesis of the SOB and MOB with their free-living relatives with sequenced genomes (**Fig. 5, Supplementary Figure 3, Supplementary Figure 4**). However, due to a lack of genome resources of its close relatives, we are not sure whether the nutrients metabolism pathways of both SOB and MOB are unique (**Fig. 5**, **Supplementary Figure 3, Supplementary Figure 4**). When the genomes of their closer relatives are available in the future, we can further check to see whether the *Gigantopelta aegis* symbionts are indeed unique with respect to their nutrients metabolism pathways in a phylogenetic context.

Kuwahara, H. et al. Reduced genome of the thioautotrophic intracellular symbiont in a deep-sea clam, *Calyptogena okutanii*. *Curr. Biol.* **17**, 881–886 (2007).
Newton, I. L. G. et al. The *Calyptogena magnifica* chemoautotrophic symbiont genome. *Science* **315**, 998–1000 (2007).

5) In my opinion, the Results and Discussion section does not give enough background information, probably due to the authors aim to stay within the word count constraints. The arguments on functions of different proteins and the novelty of their findings do not have enough space to be comprehensible. With a short manuscript appropriate for Nature Communications, it would seem prudent to select the key findings and present confirmatory or obvious observations in a supplementary text section. The reader would benefit from having less results reported and in return more information on the individual findings. The current density of the manuscript seems crammed and at the same time superficial in parts.
Response: We agree that some parts of the Results and Discussion in the previous version were not clearly presented. To address the reviewer's concern, we have elaborated more about the functions of proteins, especially for "Genetic Control of Symbiont Acquisition". We have also provided more details on the key findings regarding as "Evolution of Symbiosis in Deep-sea Peltospirid Snails", "Genetic Control of Symbiont Acquisition", "Energy Resources" and "Holobiont Nutritional Interdependence". We have also provided more explanation and background information to improve the readability of the manuscript.

6) Generally, the Supplementary Tables and Figures could be better structured to aid the reader, e.g. by streamlining them according to where they are referenced in the manuscript (e.g. line 190 ff). In the current form, the reader has to jump different Supplementary figures for some statements. I suppose there are better ways of merging these very helpful resources

to make the data/information easier to find and comprehend. Please also invest in the figure legends, they are often missing critical information on abbreviations (e.g. Figure 4, where the abbreviations of the genes should be listed more clearly or figure S11 where there is not scale for the level of gene expression.

Response: We have followed the suggestions to reorganise the supplementary materials, which included Supplementary Figures, Supplementary Tables, Supplementary Notes, and Supplementary Data. For the Supplementary Figures, Supplementary Tables, and Supplementary Notes, we have provided a table of contents with page numbers to make sure they are easy to read and follow. Supplementary Data that contain too many data to show as tables are in included in Excel files as per the journal's requirement. All the computer commands and codes used in the study are included in a Text file as Supplementary Data. The order of all these supplementary materials are followed in the same citation order in the main text. They have been all clearly cited in the main text.

All figure legends have been further checked and revised accordingly to make sure they contain sufficient information for easy reading. The abbreviations of the gene families have been added in the figure legends of **Fig. 6** (formerly **Fig. 4**). The scale of gene expression has already been added into the **Supplementary Figure 13** (formerly Figure S11).

Specific comments:

Abstract:

Line 31: endosymbioses with more than one symbiont are just as common as those with only one symbiont and several marine examples are well researched - To only name three across different animal phyla: Bathymodiolus, Olavius, Trichoplax

Response: We agree with this comment and therefore have removed "endosymbiosis with more than one symbiont is uncommon" accordingly (**see line 35-37 of main text**).

Line 32: Dual symbiosis usually means two symbiont types and one host.

Response: We have removed "Dual" accordingly (**see line 36 of main text**).

Line 41: An enhanced immune capacity that likely facilitates the possession of more than one type of symbiont is difficult to understand. In my opinion, if an animal can host multiple distinct symbionts, its immunity is at most adapted but not enhanced. It could even be interpreted as being compromised and not as restrictive as those of animal hosts that only

harbor one symbiont type or no permanent microbiome at all.

Response: We agree with reviewer's comment. According to the results of gene abundances and expression level of pattern recognition receptors (**Fig. 7**), many of these genes are expanded in the *G. aegis* genome and highly expressed in the oesophageal gland comparing to *Chrysomallon squamiferum* with only one symbiont. And thus, *G. aegis* likely has remodelled its immune system to facilitate the possession of more than one type of symbiont. This sentence has been revised accordingly in the main text (**see line 43-45 of main text**).

Introduction:

Line 52: Not all microorganisms live in "harmony" with animals (e.g. parasites).

Response: We have revised this sentence accordingly (**see line 56 of main text**).

Line 60 and following: "Several species of annelids and molluscs, however, are capable of hosting multiple co-occurring endosymbionts" is not backed by references 7,8,9,10 as no annelids refs are included. Annelids with multiple symbiont lineages are e.g. shallow water gutless oligochaetes, but no refs for them are given

Response: "Dubilier, N. et al. Endosymbiotic sulphate-reducing and sulphide-oxidising bacteria in an oligochaete worm. *Nature* **411**, 298–302 (2001)." has been added here as a reference (**see line 70 and ref 11 of main text**).

Line 61: This statements needs to be more specific to focus on chemosynthetic symbioses. Even there work on e.g. Riftia pachyptila in the deep sea or Paracatenula in the shallow water is missing, both explore the host and symbiont combined gene expression

Response: We have revised this sentence and included the suggested papers on the tubeworms *Riftia pachyptila* (Hinzke et al. 2019), *Paraescarpia echinospica* (Yang et al. 2019), and the flatworm *Paracatenula* (Jäckle et al. 2019). **Please see line 72-73 of main text**.

Hinzke, T. et al. Host-microbe interactions in the chemosynthetic *Riftia pachyptila* symbiosis. *Mbio* **10**, e02243-19 (2019).

Yang, Y. et al. Genomic, transcriptomic, and proteomic insights into the symbiosis of deep-sea tubeworm holobionts. *ISME J.* **14**, 135–150 (2019).

Jäckle, O. et al. Chemosynthetic symbiont with a drastically reduced genome serves as primary energy storage in the marine flatworm *Paracatenula*. *Proc. Natl. Acad. Sci.* **116**, 8505–8514 (2019).

Line 65: 'Hologenome is key to understanding how the partners cooperate' - This is an overstatement – in my opinion, the key is the specific expression of a small subset of genes on a cellular or organ scale

Response: We agree with the reviewer on this point and have revised the sentence as "Adopting a total 'hologenome' approach sequencing the host and the symbiont genomes as well as a transcriptome approach examining organ specific gene expression enables us to disentangle the complex metabolic relationships of the partners allowing them to flourish in extreme environments" (**see line 74-77 of main text**).

Line 69 why most extreme? Here but also elsewhere in the text - please try to avoid overstatements and self-advertisement, even of your study object. Focus rather on the interesting biology we see

Response: "Most extreme" has been removed from this sentence (**see line 79-83 of main text**). The main text is checked throughout for such overstatements and revised accordingly.

Figure 1 15 pseudochromosomes are the basis of both panels b and c. The two figure panels could be combined and both shown linearly as c for easier access

Response: We tried but found it difficult to combine the two figures, because the scale of figure 1b is the chromosome length while the scale of figure 1c is based on the genes number. The former Figure 1c is removed from the main text. And we create a new expanded Figure 4 for synteny to replace the former Figure 1c. In the response to the related comment on "Line 115", we have presented the chromosome-scale synteny in dot plots to compare among the genomes of *Gigantopelta aegis* (15 chromosomes), *Chrysomallon squamiferum* (15 chromosomes), *Pomacea canaliculate* (14 chromosomes), and *Mizuhopecten yessoensis* (19 chromosomes) (**Fig. 4**).

Line 71: What do the autors mean by completely reliant on the host for the supply of methane and sulphide? Even though the symbionts are not in the gills, I would suppose that the gases can still diffuse in and out, right? Here, references and a schematic are missing to explain the current status of our understanding how this symbiosis functions.

Response: The endosymbionts housed in internal organs without direct contact with the vent fluids are likely to be reliant on the host for transporting reducing substances. For example, the endosymbionts housed in an internal trophosome of the tube worm *Riftia pachyptila* rely on the host haemoglobin to transport oxygen and sulphide (Minic, Z. & Hervé, G. 2004). Since the symbionts of *Gigantopelta aegis* and *Chrysomallon squamiferum* are housed in an internal oesophageal gland, the symbionts may also rely the host for supplies of the gases. We also agree that the gases may diffuse in and out, and this possibility cannot be excluded entirely, but the situation in this symbiosis is much more similar to *Riftia* which mostly rely on the host to transport sulphide and oxygen (see Chen et al. 2015 and Chen et al. 2017). The related main text has been revised accordingly (**see line 83-89 of main text**).

We have added key references to this section on the current understanding of this symbiosis. We refrain from drawing a schematic at this point, since we are still uncertain about how the hydrogen sulphide, methane etc. are transported. Most of the information available are from anatomy of the host which require future study to validate.

Chen, C. et al. The heart of a dragon: 3D anatomical reconstruction of the 'scaly-foot gastropod' (Mollusca: Gastropoda: Neomphalina) reveals its extraordinary circulatory system. *Front. Zool.* **12**, 13 (2015).

Chen, C. et al. By more ways than one: rapid convergence at hydrothermal vents shown by 3D anatomical reconstruction of *Gigantopelta* (Mollusca: Neomphalina). *BMC Evol. Biol.* **17**, 62 (2017).

Minic, Z. & Hervé, G. Biochemical and enzymological aspects of the symbiosis between the deep-sea tubeworm *Riftia pachyptila* and its bacterial endosymbiont. *Eur. J. Biochem*. **271**, 3093–3102 (2004).

Results and Discussion:

Line 107: The size difference between the two host genomes seems large – the authors report a genome difference of 1 GB which they explain with the large number of repetitive regions. Is this backed up with other data, e.g. estimates based on kmer spectral analysis using illumina short reads?

Response: Yes. This conclusion is backed up with kmer spectral analysis using Illumina short reads of *Gigantopelta aegis*. In the present study, a 17 mer frequency (**Supplementary Figure 19**) was used to predict repeats proportion of *G. aegis* host genome. It is predicted

that the repeats occupy 50% of the host genome, which supports the results that the repeats annotated from the genome is around 51% of the genome. *Chrysomallon squamiferum* genome is suggested to be compact, which is not very common in Mollusca (Sun et al. 2020). The repeat content of *C. squamiferum* is only predicted to be 25.2% (Sun et al. 2020). Therefore, the major difference of the genome size between the two hosts is due to the repeat content being much less in *C. squamiferum*.

Sun, J. et al. The scaly-foot snail genome and the ancient origins of biomineralised armour. *Nat. Commun.* **11**, 1657 (2020).

Line 115: How does the synteny between G. aegis and C. squamiferum compare with other Mollusc genomes (if available)? Is this strong chromosomal conservation and intra-chromosomal rearrangement common in gastropods or other invertebrate phyla when comparing genomes from different genera that have evolved separately for more than 100 million years?

Response: Due to the small number of chromosome-level genome assemblies within gastropods and molluscs in general, at this time we are unable to test if such a high macro-synteny conservation is the norm within the group. No other pairs of chromosomal-level assemblies for molluscan genera separated by about 100 mya are currently available, and we have noted this in the main text. According to macro-synteny analyses in Wang et al. 2017, different phyla have very different synteny rearrangement patterns compared to the presumed ancestral bilaterian pattern, and as such we do not consider comparing a pair from another phylum to be useful in understanding evolutionary patterns in molluscan karyotypes.

Due to the small number of chromosome-level genome assemblies within molluscs, at this time we are unable to test if such a high macro-synteny conservation is the norm within the group for a similar divergence time. Nevertheless, the macro-synteny patterns seen in these two species are also notably conserved compared the caenogastropod *Pomacea canaliculata* and the bivalve *Mizuhopecten yessoensis* (**Fig. 4**), distantly related to peltospirid snails within Mollusca, also had rather conserved chromosome-scale synteny (Wang et al. 2017; Sun et al. 2019). This is suggestive that in most molluscan lineages, intra-chromosomal rearrangements likely underpin the evolution of genetic regulatory networks and related functions, but how exactly warrants further studies. Among other molluscs with chromosomal-level assembly, the oyster *Crassostrea gigas* (CI: 0.27) uniquely has a much reduced genome size and highly

derived karyotype (Wang et al. 2017), but this is likely an exception rather than the norm among molluscs. We revised the relevant section of the main text to make these points clear (**see line 194-210 of main text**).

Wang, S. et al. Scallop genome provides insights into evolution of bilaterian karyotype and development. *Nat. Ecol. Evol.* **1**, 0120 (2017).
Sun, J. et al. Signatures of divergence, invasiveness, and terrestrialization revealed by four apple snail genomes. *Mol. Biol. Evol.* **36**, 1507–1520 (2019).

Line 127: Not sure which symbiont is which – needs to be illustrated in figure. Also the outline of a bacteriocyte would help here. Supplement figures need more detailed labelling.
Response: We have added labels for the SOB and MOB in the TEM images in main text and supplementary figures (**Fig. 2a**, **Supplementary Figure 2b, Supplementary Figure 2c**). We also outlined the bacteriocytes in the supplementary figures (**Supplementary Figure 2a, Supplementary Figure 2b**).

Line 130: How did the authors check symbiont abundance? Relative abundance in metagenomes?
Response: Both TEM results and sequencing coverage of the symbiont genome were checked to understand the symbiont abundance. From the TEM results, the symbionts with stacked membranes (MOB) were much fewer than the symbionts without stacked membranes (SOB) (**Supplementary Figure 2**). In the genome binning results, the average sequencing coverage of the SOB (~ 3,050 X) is much higher than that of the MOB (~410 X) (**Supplementary Figure 5**, **Supplementary Table 3**). The TEM results and sequencing results are consistent showing that the SOB is much more abundant than the MOB.

Line 166: Related to the symbiont phylogenetic reconstruction (Figure 3b): It creates a distorted picture of ‘purely symbiotic clades when presented as by the authors. I would ask to generate separate phylogenies and include as much of a diversity of the related bacteria as the taxonomic scope you are interested in. In this framework, maybe to the genus level would be a reasonable cutoff. Supplementary trees with 16S rRNA gene sequences would be helpful as well.
Response: To address the reviewer’s concern, we have moved the former **Fig. 3b** to **Supplementary Figure 9**. We have constructed the separate phylogenetic tree of

Gigantopelta aegis SOB and MOB as well as their relatives with genomes based on their single-copy orthologs (**Supplementary Figure 15**). Their closest relatives with genomes are used for metabolic comparative analysis. Corresponding phylogenetic trees based on16S rRNA genes, with more terminals for both *G. aegis* SOB and MOB, have also been constructed and placed in the supplementary materials (**Supplementary Figure 3, Supplementary Figure 4**).

Line 166: I don't follow their line of argument. Based on the presented tree, how can the autors relate the "phylogeny" of the symbionts to horizontal acquisition and to host-selection and specificity?

Response: We have removed the conclusions about horizontal acquisition and host selection from the main text.

Line 172: How do the snail symbionts compare to other chemosynthetic symbioses? There are also different host groups that live in the same habitat but acquire different gammaproteobacterial SOB, right? Do other studies also assume independent origins of endosymbiosis (= convergent evolution?)

Response: There are different host co-occurring in the same habitat housing different gammaproteobacterial SOB. Besides *G. aegis* and *C. squamiferum* co-occurring in the Longqi field host different gammabacterial SOB, *Alviniconcha strummeri* and *A. kojimai* snails co-occurring in the Lau Basin host different gammabacterial SOB (Beinart et al. 2019). And the tube worms *Escarpia spicata*, *Lamellibrachia luymesi*, *Seepiophila jonesi* living in the seep of Gulf of Mexico also house different gammaproteobacterial SOB (Li et al. 2019).

Referring to the response of Major comment #1, peltospirids have the evidence showing two independent origins within the same family for a convergent evolution of endosymbiosis. However, several studies suggest a single origin of the endosymbiosis. For example, (1) provannid snails clearly have a single origin of endosymbiosis (Breusing et al. 2020); (2) Vesicomyidae has a single origin of endosymbiosis in Pliocardiinae (Johnson et al. 2017); (3) Both Lucinidae and Solemyidae clams have one single origin because all known members are chemosymbiotic (Taylor & Glover 2010). Thyasiridae is not sure. It likely has one origin and many genera losses, but it could also be separate origins (Taylor & Glover 2010); (4) In the response to Major comment #1, we have discussed that *Bathymodiolus* mussels are more likely to have a common symbiotic ancestor (Lorion et al. 2013; Miyazaki et al. 2010); (5)

We don't really know siboglinids, but since they all rely on endosymbiosis, they likely have one origin and then shifted to different symbiosis types. At least, the "Vestimentifera" tubeworms do (Li et al. 2017).

Beinart, R. A., Luo, C., Konstantinidis, K., Stewart, F. & Girguis, P. R. The bacterial symbionts of closely related hydrothermal vent snails with distinct geochemical habitats show broad similarity in chemoautotrophic gene content. *Front. Microbiol.* **10**, 1818 (2019).

Breusing, C. et al. Allopatric and sympatric drivers of speciation in *Alviniconcha* hydrothermal vent snails. *Mol. Biol. Evol.* **msaa177** (2020).

Johnson, S. B., Krylova, E. M., Audzijonyte, A., Sahling, H. & Vrijenhoek, R. C. Phylogeny and origins of chemosynthetic vesicomyid clams. *System. Biodivers.* **15**, 346–360 (2017).

Li, Y. et al. Phylogenomics of tubeworms (Siboglinidae, Annelida) and comparative performance of different reconstruction methods. *Zool. Scr.* **46**, 200–213 (2017).

Taylor, J. D. & Glover, E. A. (2010). Chemosymbiotic bivalves. In *The vent and seep biota* (pp. 107–135). Springer, Dordrecht.

Lorion, J. et al. Adaptive radiation of chemosymbiotic deep-sea mussels. *P. Roy. Soc. B-Biol. Sci.* **280**, 20131243 (2013).

Miyazaki, J. I., de Oliveira Martins, L., Fujita, Y., Matsumoto, H. & Fujiwara, Y. Evolutionary process of deep-sea *Bathymodiolus* mussels. *PLoS ONE* **5**, e10363 (2010).

Li, Y., Liles, M. R. & Halanych, K. M. Endosymbiont genomes yield clues of tubeworm success. *ISME J.* **12**, 2785–2795 (2018).

Yang, Y. et al. Genomic, transcriptomic, and proteomic insights into the symbiosis of deep-sea tubeworm holobionts. *ISME J.* **14**, 135–150 (2019).

Line 172: How is that a contrast? The symbionts in Alviniconcha are also inside the host so hosting symbionts "inside" does clearly not mean higher selectivity. I would caution against such simplifications – e.g. Zimmermann et al https://onlinelibrary.wiley.com/doi/full/10.1111/mec.13554 showed higher specificity of ectosymbionts compared to endosymbionts for the same genus of symbionts.

Response: We agree with the reviewer's comment. The sentence is not accurate. We have removed this sentence from the main text.

Line 195: The evidence for the function of galectin and fucolectin has not been studied in the reference provided by the authors. Wippler et al. (2016), reference 22 in the manuscript, only

report the presence of these lectins in the transcriptome/proteome of the symbiotic worm Olavius algarvensis. To suggest a specific function as the authors do, other references and a more detailed analysis would be required. If it is not possible to provide more experimental evidence for the function of these lectins, the authors should be more cautionary with their statement.

Response: We have followed the suggestions to provide proper references for function of lectins, and toned down their potential roles in the deep-sea snails (**see line 242-245 of main text**).

Line 202: Highly expressed compared to what? Other snails, tissues or other genes in the transcriptomes?

Response: We compared the gene expression in the oesophageal gland against gene expression in other tissues (e.g. foot, ctenidium, mantle) in the same snail. If genes had a fold change of reads counts larger than two and a significant FDR *P*-value (< 0.05) generated from edgeR, they were designated as highly expressed genes. We have revised the sentence (**see line 254-257 of main text**) and provided our criterion of high expression (**see line 715-719 of main text**).

Line 204: This part appears very speculative – what digestion process are the authors talking about, lysosomal digestion? Table S12 was difficult to grasp for me, as I would have expected a comparison/relation of the expression of the concerned genes to the rest of the transcriptomes. How do the authors rate "high expression"? A reference gene/pathway or normalization is missing.

Response: Yes. We meant to say lysosomal digestion is important (**Supplementary Table 5,** formerly Table S12) because lysosome KEGG pathway was enriched with 22 genes highly expressed in the oesophageal gland of *Gigantopelta aegis*. We have included two heat maps of *Gigantopelta aegis* and *Chrysomallon squamiferum* showing the gene expression level of genes involved in the lysosome process (**Supplementary Figure 11, Supplementary Figure 12**).

The differential expression analysis was performed though compare the reads count of oesophageal gland to the sum reads count of foot, ctenidium, and mantle (*Gigantopelta aegis*: $n = 4$, *Chrysomallon squamiferum*: $n = 3$) using edgeR based on the reads counts. edgeR normalizes the sequencing depth in its basic modeling procedure when calculating fold-

change and *P* value. The gene with a fold change larger than two and a significant FDR *P* value is considered as a gene highly expressed in the oesophageal gland.

Before plotting the heat map of host gene expression, we normalized gene expression in different organs by CPM (counts per million) values by using STANDARDIZE function implemented in Excel. The scale of the heat map is the normalized CPM value. The data for plotting the heat maps were provided in the source data (**Supplementary Data 7**).

Line 207: Why is it expected that environmental chemical compounds fluctuate in concentration? I generally agree with this statement, but timeframes and environmental data would be helpful, maybe as a reference if they are already published?

Response: The timeframes data is lacking for our study area, but the concentrations of environmental chemical compounds, such as methane, hydrogen sulphide and hydrogen are different in different zones of Longqi field showed in the Supplementary Table 1 of Tao et al. 2020. This kind of pattern may be expected for Kairei field in Central Indian Ridge and all Mid-Ocean Ridge vents, although at different levels and rates of fluctuations (Desbruyères et al. 2001; Miyazaki et al. 2020). The sentence is revised accordingly (**see line 167-169 and line 290-292 of main text**).

Tao, C. et al. Deep high-temperature hydrothermal circulation in a detachment faulting system on the ultra-slow spreading ridge. *Nat. Commun.* **11**, 1300 (2020).

Miyazaki, J. et al. Dual energy metabolism of the Campylobacterota endosymbiont in the chemosynthetic snail *Alviniconcha marisindica*. *ISME J.* **14**, 1273–1289 (2020).

Desbruyères, D. et al. Variations in deep-sea hydrothermal vent communities on the Mid-Atlantic Ridge near the Azores plateau. *Deep Sea Res. PT. I* **48**, 1325–1346 (2001).

Line 214-221: Interesting – I would love to know how widespread these proteins are across other environmental or symbiotic bacteria.

Response: Peptidoglycan-associated lipoprotein (Pal), OmpA family proteins and OmpH family proteins are widespread in environmental and symbiotic bacteria living in both marine and non-marine environments. Pal is a ubiquitous outer-membrane protein in Gram-negative bacteria, such as *Escherichia coli* and the pathogens *Haemophilus influenzae*, that are

widespread in various environments (Liang et al. 2005). Based on the searching results in NCBI protein databases, Pal can be also found in free-living bacteria in marine water and sediments, such as *Sulfitobacter* sp. SK025 living in marine sediment, and *Erythrobacter jejuensis* isolated from seawater. Furthermore, many symbionts, such as the symbionts of insects living on land (i.e. stinkbug *Plautia stali* and mosquito *Aedes aegypti*), symbionts of land plants (partridge pea *Chamaecrista fasciculata* and soybean), symbionts of shallow-water marine animals (i.e. *Solemya* clams and gutless oligochaete worms *Olavius*) and symbionts of animals living in deep-sea chemosynthetic environments (*Calyptogena* clams, *Bathymodiolus* mussels, *Iophon* sponges, and siboglinid tubeworms) have Pal. The horizontal transmission mode of the animals (i.e. *Bathymodiolus* mussels and siboglinid tubeworms) indicates that the free-living status of the symbionts in the ambient environments also have Pal proteins.

The OmpA family proteins are also widespread in mainly Gram-negative bacteria, such as *Escherichia coli* and pathogenic bacteria including *Pseudomonas aeruginosa*, *Haemophilus influenzae* and *Klebsiella pneumoniae* (Confer & Ayalew 2013). Based on the searching results in NCBI protein databases, OmpA family proteins can be found in free-living marine bacteria, such as *Vibrio diabolicus*, *Jejuia pallidilutea*, and *Shewanella piezotolerans*. The OmpA can be also found in symbionts of insects (i.e. stinkbug *Plautia stali* and *Halyomorpha halys,* psyllid bug *Bactericera*, bumble bee, and aphid *Cinara cedri*), symbionts of shallow-water animals (i.e. *Solemya* clams, gutless oligochaete worms *Olavius*, ponyfish, and freshwater hydroid *Hydra*), and symbionts of animals living in deep-sea chemosynthetic environments (i.e. siboglinid tubeworms, *Bathymodiolus* mussels, and vesicomyid clams). The horizontal transmission mode of the animals (i.e. *Bathymodiolus* mussels and siboglinid tubeworms) indicates that the free-living status of the symbionts in the deep-sea ambient environments also have OmpA proteins.

The OmpH family proteins are also widespread in Gram-negative bacteria from marine and non-marine environments. They can be found in *Escherichia coli*, deep-sea free-living bacteria *Photobacterium profundum*, ubiquitous marine bacterium *Alcanivorax borkumensis*, marine pathogens *Vibrio anguillarum* and *Tenacibaculum maritimum*, water and soil bacteria *Burkholderia cepacian*, as well as plant pathogen *Pantoea*. The OmpH family proteins are found in the symbiont of insects (i.e. stink bug *Plautia stali*), symbionts of shallow-water animals (i.e. clam *Solemya*) and symbionts of animals living in deep-sea chemosynthetic

environments (i.e. *Iophon* sponge, *Bathymodiolus* mussels). The free-living status of those symbionts also have the OmpH family.

Liang, M. D. et al. Bacterial peptidoglycan-associated lipoprotein: a naturally occurring toll-like receptor 2 agonist that is shed into serum and has synergy with lipopolysaccharide. *J. Infect. Dis.* **191**, 939–948 (2005).

Confer, A. W. & Ayalew, S. The OmpA family of proteins: roles in bacterial pathogenesis and immunity. *Vet. Microbiol.* **163**, 207–222 (2013).

Line 231-233: The sentence was difficult for me. Could you please explain in simpler terms what is being utilised "respectively" by which symbiont.

Response: To simplify the sentence, we have revised it as "The SOB has the capability to utilise thiosulphate, sulphite, sulphide, nitrate, nitrite, carbon oxide as well as hydrogen, and the MOB can utilise methane, nitrate, nitrite as well as hydrogen. Together, these two symbionts form a dual symbiosis system highly versatile in utilising chemical substances (**Fig. 8a**)." (**see line 302-305 of main text**).

Line 289: Genes for nitrate respiration are indeed found in methane-oxidising bacteria of Bathymodiolus spp. mussels

Response: We agree with the reviewer's comment. We revised the sentence accordingly (**see line 378-380 of main text**). The nitrate respiration of deep-sea symbionts have been summarised in the **Supplementary Table 8**.

Line 295: This is an example for my general critique about Supplementary figures and tables – I am missing information about the level of gene expression shown in the heatmap. What do the colours represent? Also, why are different "Sialin-like" genes expressed in the different tissues as shown with the heatmap. Or do the authors refer to transcripts/isoforms in this case?

Response: The colour represents the gene expression level (normalized CPM value). The red colour represents high expression level and the light colour represents low expression level. The different "Sialin-like" genes represent for multiple copies (paralogs) of sialin-like genes in the genome, rather than the transcripts/isoforms generated in the transcription process. We have provided a better explanation of the colour codes in the figure legends of all heat maps to make them easier to read.

Line 300-304: I was wondering how the SOB of C. squafimerum, that does not host another symbiont, obtain the lacking intermediates of the TCA cycle if not from the other symbiotic partner? Or does this host contain another symbiont other than the SOB? Or are the authors only referring to the host G. aegis? This is not clear for me as written.

Response: This sentence only refers to the host *G. aegis*. The SOB of *C. squamiferum* has a complete TCA cycle (Nakagawa et al. 2014), but the SOB of *G. aegis* lacks a complete TCA cycle and needs to get the replenishment from other symbiont partners. We have revised this sentence to avoid confusion (**see line 392-394 of main text**).

To further confirm the number of symbiont phylotypes in *C. squamiferum*, we also obtained bacterial 16S rRNA gene amplicon sequencing data from the oesophageal gland of three individuals of *C. squamiferum*. The data show that *C. squamiferum* only have one dominant symbiont phylotype, which is identical to the SOB reported by Nakagawa et al. 2014. This new 16S rRNA amplicon sequencing data will also be released in the NCBI database when the manuscript is ready to be accepted by the journal.

Nakagawa et al. Allying with armored snails: the complete genome of gammaproteobacterial endosymbiont. *ISME J.* **8**, 40–51 (2014).

Line 310: What are the authors referring to? There are only “Biological Modules” listed. I assume the authors link “host lysis symbiont cells” to the Biological Module “Lysosome”. I would love to see more detailed information here. As it is written, the reader needs to infer/imagine what the authors are referring to and how they link the observed patterns to symbiont lysis. Also, there is an “of” missing.

Response: Indeed we intended to say that the Biological Module “Lysosome” was enriched with highly expressed genes in the oesophageal glands. To show the high expression of the many lysozyme genes, we have constructed tissue specific expression heat maps for *Gigantopelta aegis* and *Chrysomallon squamiferum* (**Supplementary Figure 11, Supplementary Figure 12**). We have revised this sentence to avoid confusion (**see line 408-411 of main text**).

Line 352: “not particularly closely related” is very subjective. Please provide the reader with a generally established framework as e.g. introduced

in https://doi.org/10.1016/j.dsr2.2012.12.004. Compared to other hydrothermal species within the Mollusca, the two hosts are actually quite closely related. Yet the hosts are ~110 million years apart and both genera predate the current interpredation of a cenozoic radiation of extant vent fauna. Were they both symbiotic before the current radiation? Did they have a symbiotic last common ancestor? And what are their closest relatives that are non-symbiotic, could you estimate this as well and put the two symbiotic genera in perspective?

Response: Please see the response to your Major Point #1 on the background of Peltospiridae and that all other genera are not symbiotic. The phylogenetic evidence for this comes from previously published multi-gene phylogenies with higher taxon sampling and we cite this (Chen et al. 2017), and we have added more information about this including genera names in the main text. Early fossils of peltospirids exist from Early Cretaceous seeps already (Kaim et al. 2014), and they were small-sized and unlikely to have been symbiotic (this is now added to the main text). Therefore, the fossil evidence supports the two genera evolved symbiosis separately, after the current radiation of peltospirids began. *Dracogyra subfuscus* is the closest relative (sister genus) of *Gigantopelta* as far as best we know (Chen et al. 2017). From our new data, it really does not rely on symbionts, which is supported by our genome binning results (**Supplementary Figure 8**) and anatomical investigations (Chen et al. 2017). Referring to your first Major point, our phylogenomic analysis shows that *D. subfuscus* is more closely related to *G. aegis* than *C. squamiferum* (**Fig. 3**, **Supplementary Figure 24**). These results are in line with previous published phylogenies (Chen et al. 2017) that *G. aegis* and *C. squamiferum* have independently acquired their symbiosis.

Chen, C., Zhou, Y., Wang, C. & Copley, J. T. Two new hot-vent peltospirid snails (Gastropoda: Neomphalina) from Longqi hydrothermal field, Southwest Indian Ridge. *Front. Mar. Sci.* **4**, 392 (2017).

Kaim, A., Jenkins, R. G., Tanabe, K. & Kiel, S. Mollusks from late Mesozoic seep deposits, chiefly in California. *Zootaxa* **3861**, 401–440 (2014).

Reviewer #1 (Remarks to the Author):

The responses to my previous comments are more than sufficient. I have no further criticisms. Well done.

Reviewer #2 (Remarks to the Author):

I congratulate the authors to the substantially improved manuscript. The massive effort the author have invested has paid off, all my major concerns are addressed. I am particularily impressed by the authors ability to provide a draft genome of a closely related peltospirid that is not symbiotic to corroborate their phylogenetic reconstructions and evolutionary interpretations.

I have noted several minor things that the authors might want to fix. As a side note, and please do not see this as scientific critique, the manuscript is much easier to read and follow in this revision, but maybe the authors could get a nice finishing touch if the manuscript was polished by a professional language editor.

One issue that needs some work on the text is the use and transport of oxygen in the holobiont. L302 for example does not list oxygen for the SOB, but I would guess that they can use both oxygen and nitrate as terminal electron acceptors. L339 correctly calls the MOB aerobic, yet again oxygen use is not discussed. MOB have high oxygen demands, and the ability to switch to nitrate for respiration does not cover all the MOBs oxygen demands. The host is an aerobe as well, and the authors then appropriately summarize that within an aerobic host and between two aerobic symbionts, mechanisms to alleviate competition are beneficial (L387ff) but that is not apparent from the paragraphs before and also mechanistically not well explored.

Could the authors add the information on the dating directly into Fig 3, e.g. right with the time axis and with links to the respective nodes?
Fig. 4 could use some more information – e.g. by labelling the comparisons according to the taxon indicators used in Fig. 3. This way the reader could more easily connect the dots.
Figure 5 is very helpful, could you add some kind of indicators for the columns that are important and discussed in the text?

Nitty gritty details:
L49 genomes, not genome
L67 In contrast, - instead of while
L69 Olavius is the only shallow water taxon listed here, please add shallow water
L95 there is an 'in' missing – in each generation
L102 delete 'while'
L139 … more than twice as large as that of … instead of double of
L158 you are using specific probes, please mention this here
L204 compared to …
L209 what does CI: 0.27 mean?
L214 relies
L227 I would suggest to rephrase to Despite the phylogenetic divergence between …
L229 nutritional
L230 replace genomic with phylogenetic
L233ff is repetitive, you could give the details of the aa and vitamins/co-factors
L247 … G. aegis much more abundantly harbours …
L397 the C4 compounds could also come from the host, so I would suggest to use … one of the other symbiotic partners.
L447/448 replace dominant with abundant
L480 The additional TEM images are sufficient, but they show strong fixation artifacts. Nothing the

authors could do about, but it would be great if they used glutaraldehyde based fixation the next time they wanted to fix for TEM.
L497-504 I checked with RDP probe match and the probes have no non-target hits and appear highly specific. However, the newly added section for FISH probe design description misses the step that identified the specific probe sequence. It would be good to share how you detected these highly specific sequence parts in the symbionts 16S rRNA genes. Were they identified using a program or manually? If manually, you would need an alignment of related sequences, but all these details are missing.

Reviewer #3 (Remarks to the Author):

Please note: I have been asked to comment specifically on metazoan genome assembly, and have concentrated my review on this aspect of the work.

This work by Lan et al presents interesting results on the genomic consequences of a symbiotic relationship between a deep sea snail and its endosymbiotic bacteria. In general this work is well performed. However, there are some issues that I would ask to be addressed before publication.

-I am unable to assess the veracity of the assembly and sequencing metrics as this data does not seem to be available. Even if not made fully public, this should be provided for review, e.g. through the GEO (where review links can be given). Please ensure Dracogyra subfuscus information is also provided.

-The numerous data on short Illumina genomic reads, which is presently scattered in text, should be presented in a table alongside that on PacBio/HiC sequencing. This should also be provided separately for Dracogyra subfuscus (broken down by library).

-note number of Ns present in the genome, particularly as a result of scaffolding - please give details and information on what these represent (e.g. are blocks of 1000 the results of software X)

-There are 9,842 contigs in initial assembly, 9,479 in final assembly. Please give information about where this trimming occurred, and why. Are these bacterial contaminants?

-The "HiC valid reads" are only 3% of the total number of reads sequenced (112,328,978 of 3,215,925,346=3.4%). This is a low percentage. Can the authors note this in text and speculate as to why this is the case?

-Presentation of the results of the bacterial contamination scan in general should be improved. Supplementary Figure 5 seems to indicate additional bacterial contamination may have been present. How much was removed by MaxBin or manually?
"Bacteria nucleotides were barely found in the scaffolds of G. aegis, suggesting a lack of horizontal gene transfer between the host and the symbionts." (line 207/208 supplement). Nucleotides are nucleotides and do not differ- do you mean sequences? And please give more definite information than "barely found"

-Statistics are given about the N50 etc, and the 15 pseudo-chromosomes (1.006 Gb). However, N50 starts to lose its usefulness at this level of assembly. Could an additional table be given/or added to the end of Supplementary Table 1, showing the size of each of the 15 pseudochromosomes, and the N50 results etc for the remaining 5,216 chromosomes?

- Whole Dracogyra subfuscus were used for histogram analysis and identification of bacterial sequence, vs oesophageal gland tissue for Gigantopelta aegis. Please note this in Supplementary Note 7, as it somewhat invalidates the intended comparison.

-The HiC contact map in Supplementary Figure 18 seems to show something interesting in the scaffold at bottom right. Note the off-centre areas in red, and the possible break in the centre of the sequence (region of low cross-coverage). This could indicate a mis-assembly, a region where two heterozygous regions of the genome have been placed sequentially rather than merged, or potentially a region with interesting genomic architecture. Have the authors considered these possibilities, and checked these loci?

Minor comments:
Scale missing, Supplementary figure 18

BluePippen mis-spelled, line 550 main text

Line 212, server mis-spelled

Line 424 supplement - anatina mis-spelled

REVIEWER COMMENTS

Reviewer #1 (Remarks to the Author):

The responses to my previous comments are more than sufficient. I have no further criticisms. Well done.

Response: We appreciate this encouraging comment from the reviewer.

Reviewer #2 (Remarks to the Author):

I congratulate the authors to the substantially improved manuscript. The massive effort the author have invested has paid off, all my major concerns are addressed. I am particularly impressed by the authors ability to provide a draft genome of a closely related peltospirid that is not symbiotic to corroborate their phylogenetic reconstructions and evolutionary interpretations.

Response: We appreciate these encouraging comments from the reviewer.

I have noted several minor things that the authors might want to fix. As a side note, and please do not see this as scientific critique, the manuscript is much easier to read and follow in this revision, but maybe the authors could get a nice finishing touch if the manuscript was polished by a professional language editor.

Response: We have followed the reviewer's suggestions to fix these minor issues mentioned below and the manuscript language has been checked by a native speaker.

One issue that needs some work on the text is the use and transport of oxygen in the holobiont. L302 for example does not list oxygen for the SOB, but I would guess that they can use both oxygen and nitrate as terminal electron acceptors. L339 correctly calls the MOB aerobic, yet again oxygen use is not discussed. MOB have high oxygen demands, and the ability to switch to nitrate for respiration does not cover all the MOBs oxygen demands. The host is an aerobe as well, and the authors then appropriately summarize that within an aerobic host and between two aerobic symbionts, mechanisms to alleviate competition are beneficial (L387ff) but that is not apparent from the paragraphs before and also mechanistically not well explored.

Response: We have clarified in the main text by adding the sentence: 'They are both aerobic and possess succinate dehydrogenase, cytochrome c reductase and cytochrome oxidase to utilise oxygen (**Supplementary Data 3-4**).'

Could the authors add the information on the dating directly into Fig 3, e.g. right with the time axis and with links to the respective nodes?

Response: Since many fossil records were ranges and some are only either maximum or minimum bounds, we consider that including these ranges on a main figure will be confusing with the resulting confidence intervals of divergence date estimates. Nevertheless, we have indicated the nodes calibrated using fossils and geographic events using red stars on this figure, and the full dating information is available in **Supplementary Note 3** for the interested readers.

Fig. 4 could use some more information – e.g. by labelling the comparisons according to the taxon indicators used in Fig. 3. This way the reader could more easily connect the dots.
Response: The species names in each plot have been labelled in different colors to represent the taxon information. The plots in the Fig. 4 include the comparison **a.** between *G. aegis* and *C. squamiferum* (comparison within Neomphalida: black), **b.** between *G. aegis* and *P. canaliculata* (comparison within Gastropoda: red), **c.** between *G. aegis* and *M. yessoensis* (comparison within Mollusca: green); **d.** between *C. squamiferum* and *P. canaliculata* (comparison within Gastropoda: red), **e.** between *C. squamiferum* and *M. yessoensis* (comparison within Mollusca: green), and **f.** between *P. canaliculata* and *M. yessoensis* (comparison within Mollusca: green). This colour labelling scheme follows the taxon indicator colours in Fig. 3.

Figure 5 is very helpful, could you add some kind of indicators for the columns that are important and discussed in the text?
Response: We have followed the suggestion to clarified this issue. The nutrient names in Figure 5 are labelled by different colors to represent the biosynthesis capabilities of the host and symbiont. Blue (H, I, K, L, V, W, B1, B2, B7, B9, and B12) represents nutrients that can be synthesised by both the sulphur-oxidising endosymbionts (SOB) and the methane-oxidising endosymbionts (MOB) but the host lacks this ability. Orange (B3 and B6) represents nutrients that can only be synthesised by the host. Red (B5 and CoA) represents that the nutrients that can only be synthesised by the MOB. Grey represents the nutrients can be synthesised by the host, SOB and MOB.

Nitty gritty details:
L49 genomes, not genome
Response: Revised accordingly.

L67 In contrast, - instead of while
Response: Revised accordingly.

L69 Olavius is the only shallow water taxon listed here, please add shallow water
Response: Revised accordingly.

L95 there is an ‘in’ missing – in each generation
Response: Revised accordingly.

L102 delete ‘while’
Response: Revised accordingly.

L139 … more than twice as large as that of … instead of double of
Response: Revised accordingly.

L158 you are using specific probes, please mention this here
Response: Revised accordingly.

L204 compared to …

Response: Revised accordingly.

L209 what does CI: 0.27 mean?

Response: CI represents coefficient index. Since there is no actual discussion of this value in this sentence, “CI: 0.27” has been removed.

L214 relies

Response: Revised accordingly.

L227 I would suggest to rephrase to Despite the phylogenetic divergence between …

Response: Revised accordingly.

L229 nutritional

Response: Revised accordingly.

L230 replace genomic with phylogenetic

Response: Revised accordingly.

L233ff is repetitive, you could give the details of the aa and vitamins/co-factors

Response: The details of amino acids, vitamins and co-factors are provided and discussed in the “Holobiont Nutritional Interdependence” part, and thus, we didn’t provide the details here. However, to avoid the repetitiveness of the sentence, we have rephrased the sentence to “Selection of the SOB symbionts appears to have favoured the retention of biosynthesis of specific nutrients that the hosts are unable to synthesise.”

L247 … G. aegis much more abundantly harbours …

Response: Revised accordingly.

L397 the C4 compounds could also come from the host, so I would suggest to use … one of the other symbiotic partners.

Response: Revised accordingly.

L447/448 replace dominant with abundant

Response: Revised accordingly.

L480 The additional TEM images are sufficient, but they show strong fixation artifacts. Nothing the authors could do about, but it would be great if they used glutaraldehyde based fixation the next time they wanted to fix for TEM.

Response: We thank the reviewer’s suggestions. We will use glutaraldehyde method to fix samples for TEM in the future.

L497-504 I checked with RDP probe match and the probes have no non-target hits and appear

highly specific. However, the newly added section for FISH probe design description misses the step that identified the specific probe sequence. It would be good to share how you detected these highly specific sequence parts in the symbionts 16S rRNA genes. Were they identified using a program or manually? If manually, you would need an alignment of related sequences, but all these details are missing.

Response: The sequences for designing probes were manually identified. Specifically, the 16S rRNA gene sequence was searched against online standard databases of nucleotide collection in NCBI. The alignments among the 16S rRNA genes of symbionts and their 5 best hit sequences with highest sequence similarity were checked manually. The aligned region with largest difference was manually chosen for designing probes. The probe sequences were further confirmed for their uniqueness by searching against in the NCBI nt database. This information has been added in the main text.

Reviewer #3 (Remarks to the Author):

Please note: I have been asked to comment specifically on metazoan genome assembly, and have concentrated my review on this aspect of the work.

Response: We thank the reviewer's time and effort in reviewing our manuscript, especially the metazoan genome assembly part.

This work by Lan et al presents interesting results on the genomic consequences of a symbiotic relationship between a deep sea snail and its endosymbiotic bacteria. In general this work is well performed. However, there are some issues that I would ask to be addressed before publication.

Response: We thank the reviewer's comments and suggestions. We have followed the reviewer's suggestions and addressed the reviewer's concerns as follows.

-I am unable to assess the veracity of the assembly and sequencing metrics as this data does not seem to be available. Even if not made fully public, this should be provided for review, e.g. through the GEO (where review links can be given). Please ensure Dracogyra subfuscus information is also provided.

Response: We have deposited all the sequencing data and assembled data of the *Gigantopelta aegis* holobiont under BioProject PRJNA612619 (reviewer link: https://dataview.ncbi.nlm.nih.gov/object/PRJNA612619?reviewer=l6n7qvtho4vcdmc465mhcavjgg) and *Dracogyra subfuscus* under BioProject PRJNA680542 (reviewer link: https://dataview.ncbi.nlm.nih.gov/object/PRJNA680542?reviewer=8739du4rktd85aetkl2luoa1i3). Detailed data information of *G. aegis* holobiont have been provided in **Supplementary Table 8** and **10**. We have also deposited their assembled data and annotated genome information in Figshare (https://figshare.com/s/cd78462721ba9faa02e0). The metaproteome data are available via ProteomeXchange with identifier PXD022852 (reviewer account: username: reviewer_pxd022852@ebi.ac.uk, password: ENoFXaSt). These data are now accessible using the links provided. Once the manuscript is accepted, we will request NCBI to release them to the public.

-The numerous data on short Illumina genomic reads, which is presently scattered in text, should be presented in a table alongside that on PacBio/HiC sequencing. This should also be provided separately for Dracogyra subfuscus (broken down by library).

Response: The numerous data of Illumina genomic reads of both *Gigantopelta aegis* host and *Dracogyra subfuscus* have been added in **Supplementary Table 9**.

-note number of Ns present in the genome, particularly as a result of scaffolding - please give details and information on what these represent (e.g. are blocks of 1000 the results of software X)

Response: In the assembled genome of *Gigantopelta aegis*, there are 4,248 gaps with a fixed length of 200 bp. These gaps were generated when the assembled contigs were further scaffolded using Hi-C data, and these gaps represent gaps of the unknown size. This information has been added to **Supplementary Information line 214-216**.

-There are 9,842 contigs in initial assembly, 9,479 in final assembly. Please give information about where this trimming occurred, and why. Are these bacterial contaminants?

Response: The initial assembly result of 9,842 contigs are the result of wtdbg2 assembly followed with two rounds of racon and pilon polishing. MaxBin was further used to remove 517 contigs of bacterial contamination. Then, 9,325 clean contigs were manually corrected into 9,479 contigs according to Hi-C maps generated by 3D *de novo* assembly (3D-DNA) pipeline (Dudchenko et al. 2017) via Juicebox version 1.11.08 (Durand et al. 2016). This information has been added to **Supplementary Information line 193-196**.

Ref:

Dudchenko, O. et al. *De novo* assembly of the *Aedes aegypti* genome using Hi-C yields chromosome-length scaffolds. *Science* 356, 92–95 (2017).

Durand, N. C. et al. Juicebox provides a visualization system for Hi-C contact maps with unlimited zoom. *Cell Syst.* 3, 99–101 (2016).

-The "HiC valid reads" are only 3% of the total number of reads sequenced (112,328,978 of 3,215,925,346=3.4%). This is a low percentage. Can the authors note this in text and speculate as to why this is the case?

Response: The Hi-C sequencing technology depends on the chromatin formation, which is highly sensitive to the freshness of the samples. For these deep-sea animals, it is extremely difficult to keep them alive during the deep-sea cruise. Moreover, the low proportion of the valid Hi-C reads of the total reads sequenced is common in Mollusca, mainly due to the high content of mucopolysaccharide in the tissue (Sun et al. 2020). However, we tried our best to wash, fix and store the samples. When the gastropods arrived the board, tissue from the foot was immediately dissected, cut up, washed in phosphate-buffered saline buffer for many times, and stored at -80 °C freezer. The samples were immediately transported to Hong Kong with sufficient amount of dry ice. The fixed samples were never thawed until Hi-C library preparation.

We also noticed the similar ratio in our former Scaly-foot Snail genome Hi-C library preparation, and therefore, we aimed to sequence over 300X of the estimated genome size to

get enough valid reads. This information has been added in the **Supplementary Information line 198-209**.

Ref:

Sun, J. et al. The scaly-foot snail genome and the ancient origins of biomineralised armour. *Nat. Commun.* **11**, 1657 (2020).

-Presentation of the results of the bacterial contamination scan in general should be improved. Supplementary Figure 5 seems to indicate additional bacterial contamination may have been present. How much was removed by MaxBin or manually?
"Bacteria nucleotides were barely found in the scaffolds of G. aegis, suggesting a lack of horizontal gene transfer between the host and the symbionts." (line 207/208 supplement). Nucleotides are nucleotides and do not differ- do you mean sequences? And please give more definite information than "barely found"
Response: **Supplementary Figure 5** shows the genome binning results using genome sequencing data generated from the oesophageal gland (OG), the tissue harhouring the two symbionts. The additional bacterial contamination (except the two symbiont genomic contigs) in this figure represents the initial genome binning assembly results of the OG metagenome.

For *Gigantopelta aegis* host genome, the Illumina sequencing data, PacBio sequencing data and Hi-C sequencing data used for assembly were all generated from foot tissue that does not have symbionts (**Supplementary Table 8**). To ensure there is no bacterial contamination, the potential contamination of the host assembly results was checked by CheckM. The initial assembly results without using MaxBin showed that it included one bacteria genome that has 98.28% completeness and 63.56% potential contamination. After using MaxBin, 517 contigs with a total length of 10.4 Mb and a N50 of 25.2 Kb were removed. The BUSCO completeness of the host genome was not reduced after removing the bacterial contamination. The CheckM results of this clean assembly showed 0% contamination of bacteria.

These details have been added in the **Supplementary Information line 223-236**. The sentence mentioned in the comments has been revised to "bacterial sequences were barely found in the scaffolds of *G. aegis*, suggesting a lack of horizontal gene transfer between the host and the symbionts".

-Statistics are given about the N50 etc, and the 15 pseudo-chromosomes (1.006 Gb). However, N50 starts to lose its usefulness at this level of assembly. Could an additional table be given/or added to the end of Supplementary Table 1, showing the size of each of the 15 pseudochromosomes, and the N50 results etc for the remaining 5,216 chromosomes?
Response: **Supplementary Table 1** has been revised accordingly to show the data requested by the reviewer.

- Whole Dracogyra subfuscus were used for histogram analysis and identification of bacterial sequence, vs oesophageal gland tissue for Gigantopelta aegis. Please note this in

Supplementary Note 7, as it somewhat invalidates the intended comparison.
Response: This information had been added in **Supplementary Note 7**.

-The HiC contact map in Supplementary Figure 18 seems to show something interesting in the scaffold at bottom right. Note the off-centre areas in red, and the possible break in the centre of the sequence (region of low cross-coverage). This could indicate a mis-assembly, a region where two heterozygous regions of the genome have been placed sequentially rather than merged, or potentially a region with interesting genomic architecture. Have the authors considered these possibilities, and checked these loci?
Response: 3D *de novo* assembly (3D-DNA) pipeline was used for genome scaffolding with Hi-C data. This pipeline includes three steps, including 1) misassemblies detection and correction by using misjoin correction algorithm, 2) polishing the corrected contigs, and 3) scaffolding with the contact information (http://aidenlab.org/assembly/manual_180322.pdf). The former Supplementary Figure 18 is from step 1, with a series of mis-assembled contigs without correction. Contigs with detected misjoins pinpointed by JuicerBox were manually split, which resulted in 9479 contigs from the original 9325 contigs, and these contigs were further scaffolded into 15 pseudo-chromosomes. The updated contact map generated from post-corrected contigs has been updated in Supplementary Figure 18. There is no apparent chromosomal region of low cross-coverage in this map.

Minor comments:
Scale missing, Supplementary figure 18
Response: Revised accordingly.

BluePippen mis-spelled, line 550 main text
Response: Revised accordingly.

Line 212, server mis-spelled
Response: This comment is for the supplementary material -- the supplementary material has been revised accordingly.

Line 424 supplement - anatina mis-spelled
Response: Corrected accordingly, and whole manuscript checked to make sure no other instances are present.

Reviewer #3 (Remarks to the Author):

I have no further comments or concerns regarding the genome assembly and analysis. The authors have dealt with my suggestions appropriately and professionally. I have no further concerns and can recommend this work for publication.
